# Engineered Living Systems With Self‐Organizing Neural Networks: From Anatomy to Behavior and Gene Expression

**DOI:** 10.1002/advs.202508967

**Published:** 2026-02-20

**Authors:** Haleh Fotowat, Laurie O'Neill, Léo Pio‐Lopez, Megan M. Sperry, Patrick Erickson, Tiffany Lin, Michael Levin

**Affiliations:** ^1^ Allen Discovery Center At Tufts University Medford Massachusetts USA; ^2^ Wyss Institute For Biologically Inspired Engineering Harvard University Boston Massachusetts USA

**Keywords:** biorobotics, neuroengineering, plasticity, self‐organizing neural nets

## Abstract

A great deal is known about the formation and architecture of biological neural networks in animal models, which have arrived at their current structure‐function relationship through evolution by natural selection. Little is known about the development of such structure–function relationships in a scenario where neurons are allowed to grow within evolutionarily‐novel, motile bodies. Previous work showed that ectodermal tissue excised from *Xenopus* embryos, develops into a three‐dimensional mucociliary epidermal organoid ex vivo and exhibits movements distinct from age‐matched tadpoles. These ‘biobots’ are autonomous, self‐powered, and able to move through aqueous environments. Here, we report a new type of biobot, the neurobot, composed of mucociliary epidermis and neural tissue. We show that neural precursor cells implanted in explanted *Xenopus* ectodermal tissue develop into mature neurons, extending processes both toward the surface and among each other. These self‐organized neurobots exhibit unique morphology, more complex movements, and different responses to neuroactive drugs compared to non‐neuronal counterparts. Calcium imaging confirms neuronal activity in neurobots. Transcriptomics reveals increased transcript variability, expression of genes related to nervous system development, a shift toward ancient genes, and up‐regulation of neuronal genes linked to visual perception.

## Graphical abstract

Ectodermal tissue excised from *Xenopus* embryos self‐organizes into a three‐dimensional mucociliary organoid. Here, we generate a neural variant, termed neurobot, by implanting neural precursor cells. Neurobots develop mature neurons, adopt distinct morphologies, exhibit more complex motility, and respond differentially to neuroactive compounds. Imaging and transcriptomic analyses reveal active neuronal signaling, increased transcriptional variability, and enrichment of ancient and visual‐associated genes.

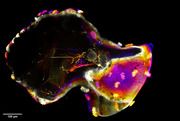

## 1 Introduction

Sensing cues from the environment and translating them into appropriate responses is the fundamental function of the nervous system in all animals. Critically, nervous systems endow animals with the ability to generate context‐ and experience‐dependent changes in their behavior. Nervous systems are also known to be plastic and adapt both structurally and functionally, on a much shorter timescale, to changes in sensory and/or motor effectors that might occur in the lifetime of an organism, for example, as a result of injury, amputation, or sensory deprivation [1, 2, 3, 4]. The capacity of an animal's nervous system to adapt to a new body plan is especially striking when the underlying sensory‐motor architecture is drastically altered. For example, ectopically induced eyes in the tails of *Xenopus* tadpoles have been shown to confer light sensitivity to otherwise eyeless hosts [5]. What are the limits of neuroplasticity in developing nervous systems? And how might wild‐type neurons establish coherent patterns and functional circuits when placed in an entirely new motile embodiment? Creating truly novel configurations of biological material allows us to probe the plasticity of evolutionary hardware to adapt on developmental (not evolutionary) timescales to truly novel circumstances and has applications for regenerative medicine, human augmentation, and biological engineering.

Two‐dimensional (2D) neuronal cell cultures grown in‐vitro provide one of the earliest demonstrations of neurons developing and functioning in a completely non‐native environment. These systems have been an extremely powerful tool for studying neural development, modeling emergent complex neural dynamics, and investigating disease mechanisms [6, 7]. Although 2D neuronal cell cultures are powerful and highly accessible systems, they lack the three‐dimensional (3D) architecture and cellular diversity of the brain, which limits their ability to model its complex circuit behaviors [8]. Recent advances in stem cell biology have enabled the creation of brain organoids, 3D self‐organizing neural tissues derived from pluripotent stem cells that can model some aspects of human neurodevelopment in vitro [8, 9]. These structures have layered neural circuits, spontaneous oscillations, early cortical‐like activity [10], have been used as a powerful tool to investigate neuropsychiatric disorders [11], and been more recently shown to have the capacity for basic forms of learning and memory [12]. Both 2D neural cultures and 3D brain organoids, however, remain non‐motile, lack the capacity to generate sensorimotor behaviors on their own, and have to be interfaced with computers or robotic systems via microelectrode arrays to perform simple tasks [13, 14, 15, 16]. Self‐contained biohybrid robots powered by muscular or neuromuscular actuation have been created by embedding muscle or combinations of neurons and muscle cells within synthetic scaffolds [17, 18, 19, 20]. These biohybrid robots, however, are not built exclusively using biological tissue, are not fully embodied, do not self‐assemble, and require external stimulation for propulsion. Another related field is that of hybrots ‐ neural structures repurposed to drive engineered bodies, such as brains and fungi operating robotic vehicles [21, 22, 23, 24]. However, engineered robotics doesn't offer the full complexity and compatibility of biological tissues.

We sought to establish a fully biological model system in which we could investigate the morphology and function of self‐organizing neural networks in novel motile embodiments, gain deeper insight into the evolutionary developmental biology of the nervous system, and inform the design of future innervated biological robots. Here, we provide the initial characterization of an inexpensive, highly‐accessible, self‐assembling biobot model to answer fundamental questions about the persistence, morphology, and functional impact of wild‐type neural cells in a non‐standard body and identify possible sensory or behavioral endpoints for future investigation.

When ectodermal tissue is excised from the animal pole of a late blastula stage *Xenopus* embryo, and allowed to develop *ex vivo*, it will develop into a self‐motile, self‐powered 3D mucociliary epithelial organoid [25, 26], which we will refer to herein as biobot. These biobots express the four cell types normally present in the embryonic skin of *Xenopus* tadpoles. These include multiciliated cells (MCCs), mucus‐secreting goblet cells, ionocytes that regulate ionic homeostasis of epidermis, and small secretory cells (SSCs) [27, 28]. MCCs function as motor effectors, generating flow through the spontaneous beating of their cilia. As a result, these biobots are capable of navigating aqueous environments, generating a suite of stereotyped movement trajectories and velocities [25]. Ciliary beating frequency is thought to be under serotonergic control, with the SSCs secreting serotonin and stimulating an increase in ciliary beating frequency through serotonergic receptors expressed on MCCs [29].

Building upon this knowledge, we set out to explore what would happen if we provided these biobots with the raw materials needed to build a nervous system. Previous theoretical work suggests that nervous systems evolved primarily to coordinate and modulate movement rather than to support complex cognition [30, 31]. From this perspective, introducing neurons into biobots offers a unique opportunity to test whether even nascent neural circuits can shape or enrich spontaneously generated behavior, providing an experimentally tractable platform for probing early principles of neural organization. Moreover, it may offer a way of testing hypotheses linking neurons, ciliary function, and movement trajectories in an accessible model, which otherwise have been limited to paleontological data [32, 33]. The availability of ciliary biobots could help test models of motion patterns inferred from the geometry and microstructure of trails left on/in sediment by Cambrian ctenophores and fossil larvae with ciliary swimming bands [34, 35].

We show that neural precursor cells harvested from *Xenopus* embryos and implanted into biobots indeed differentiate into functional neurons that extend processes both within the construct and toward its outer surface. Neurobots exhibit marked differences in morphology and behavior relative to their non‐neuronal counterparts, suggesting the emergence of neural influences on movement. Transcriptomic profiling further reveals significant upregulation of genes associated with nervous system development in neurobots compared to non‐neuronal biobots [36, 37, 38, 39], including, intriguingly, genes important for processing light stimuli. Through detailed characterization of this novel composite system, we establish a platform that reveals its key features while enabling the generation of future mechanistic hypotheses.

## 2 Results

### 2.1 Neurobots Can Be Constructed by Implanting Exogenous Neural Precursors Into *Xenopus* Ectodermal Explants

**FIGURE 1:**
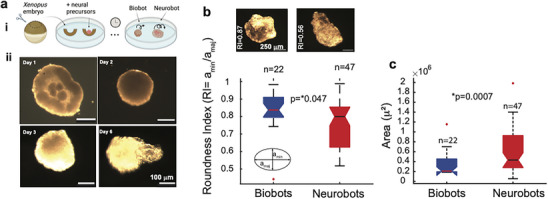
Construction and development of a neurobot. (a) Neural precursor clumps were placed in the center of an animal cap “bowl”, excised from the animal pole of a *Xenopus laevis* embryo before it fully closed during healing. The composite forms gradually into first a sphere and then a more elongated shape, which is mobile by Day 3. (b) Top panels: examples of two neurobots, one more rounded than the other. Bottom panel: Roundness Index (RI) was calculated by fitting an ellipse on the image of the bot and calculating the ratio between the minor and major axes. Neurobots tended to be less rounded than biobots (Kruskal–Wallis test, *p* = 0.047). (c) Neurobots were significantly larger than biobots (Kruskal–Wallis test*, p* = 0.0007). The central line on the box plot shows the median, and the bottom and top edges of the box indicate the 25th and 75th percentiles, respectively. The whiskers show the extent of the extreme data points not considered outliers, and the outliers are shown using the ‘+’ symbol.

To characterize the structure and function of nervous systems that self‐assemble within a novel embodiment, we established an experimental procedure for implanting biobots with neural precursor cells during the first few minutes of their formation. As shown previously, biobots can be constructed by excising tissue from the animal hemisphere of a Nieuwkoop and Faber stage 9 *Xenopus laevis* embryo (animal cap). Over the course of 30 minutes, the excised tissue gradually heals, initially forming a “bowl” shape before closing into a spherical structure [25]. We used this brief time window before tissue closure to introduce neuronal precursor cells into the interior of the healing tissue (Figure 1a (i, ii)‐top left panel).

To obtain neural cells, we took advantage of the fact that if the animal cap is excised and dissociated at the late blastula to early gastrula stage, and the dissociated cells are allowed to remain separated for 3 hours or more, they will assume a neural fate [40, 41]. To generate aggregates of neural precursors, we dissociated animal caps from approximately 50 embryos, allowed the cells to remain separated for 3 hours, and then reaggregated them. Clumps of these reaggregated cells were subsequently placed inside a freshly excised animal cap prior to its full closure (Figure 1a (i, ii), top left panel, see Methods). Within 30 minutes the formed composite assumes a spherical shape, and by the second day, it is fully healed (Figure 1a (ii), top right panel). As with non‐neuronal biobots, by the third day, multiciliated cells start appearing on their outer surface, and the bots start moving around in the dish. Similar to biobots, neurobots have a lifespan of about 9–10 days without being fed, and survive by consuming maternal yolk platelets present in all early *Xenopus* embryonic tissue [25].

Comparison of the gross morphology of neurobots and biobots revealed that, by Day 6, neurobots tend to exhibit a more elongated shape than biobots and become significantly larger (Figure 1b,c). To investigate whether the difference in size and elongation is simply due to implanting the animal caps with additional cells, we generated a third type of bot (sham neurobots) in a manner similar to neurobots, except that the implanted cells were not allowed to remain separated for 3 hours. Instead, they were reaggregated shortly after dissociation (within 30 minutes) to prevent the induction of neural fate. We found that the sham neurobots were not elongated and did not show a significant size difference compared to biobots (Figure S1a,b). These results suggest that the elongation and increase in size may be due to neuronal growth within neurobots.

### 2.2 Implanted Neural Precursor Cells Differentiate Into Neurons, Making Projections Within the Neurobot as Well as Toward the Cells Lining the Outer Surface

**FIGURE 2:**
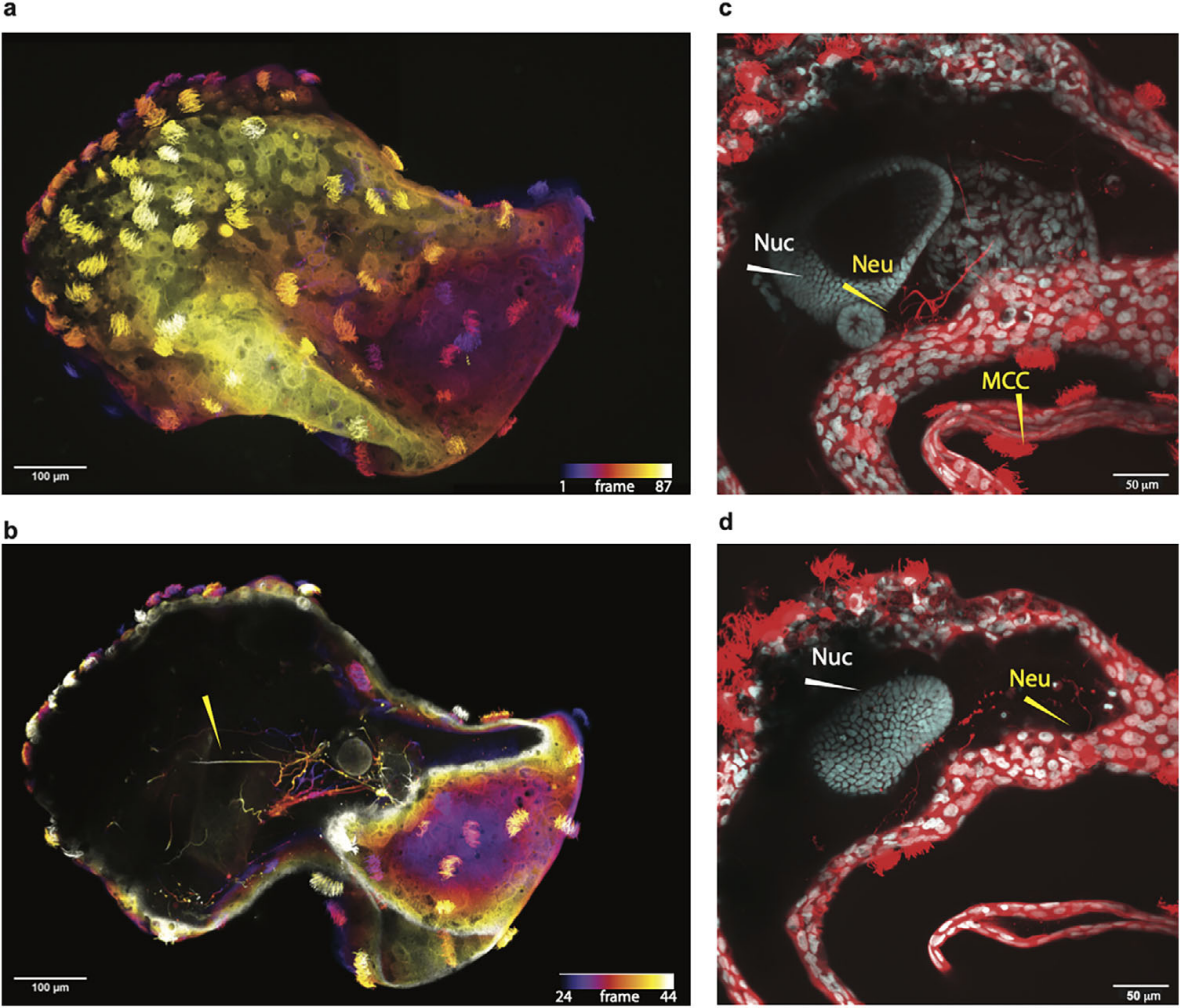
Implanted neural precursors develop into neurons and extend their processes throughout the neurobot. (a,b) *Z‐*projection of confocal image stack of a neurobot labeled with acetylated α‐tubulin, which stains neurons and cilia of the multiciliated cells. (b) is the staining of the same neurobot as shown in (a) with fewer projected planes, rendering neural processes inside the bot visible. Color code corresponds to depth within the bot (confocal plane number). (c,d) Subregions of the same neurobot, showing neural processes projecting toward surface cells. Red shows the acetylated α‐tubulin stain, and cyan depicts a nuclear (Hoechst) co‐label (Nuc). Yellow arrows point to neural processes (Neu) or multiciliated cells whose cilia are stained (MCC). White arrows point to nuclear staining (Nuc).

To determine whether the implanted cells had indeed differentiated into neurons, we fixed and stained the neurobots with an antibody that specifically binds to acetylated α‐ tubulin, which is abundantly present in neurons and multiciliated cells (see Methods; neurons and multiciliated cells are readily distinguishable from each other due to their distinctive morphology). We found that the implanted neural precursor cells indeed differentiate into neurons (Figure 2). The neurons extend their processes not only within the neurobot, but also toward the outer surface (see arrows in Figure 2c,d). Such projections toward the cells lining the surface of the bot suggest the possibility of neurons modulating the activity of surface effectors including multiciliated cells and/or the activity of those that modulate the ciliary beating frequency, for example, small secretory cells.

**FIGURE 3:**
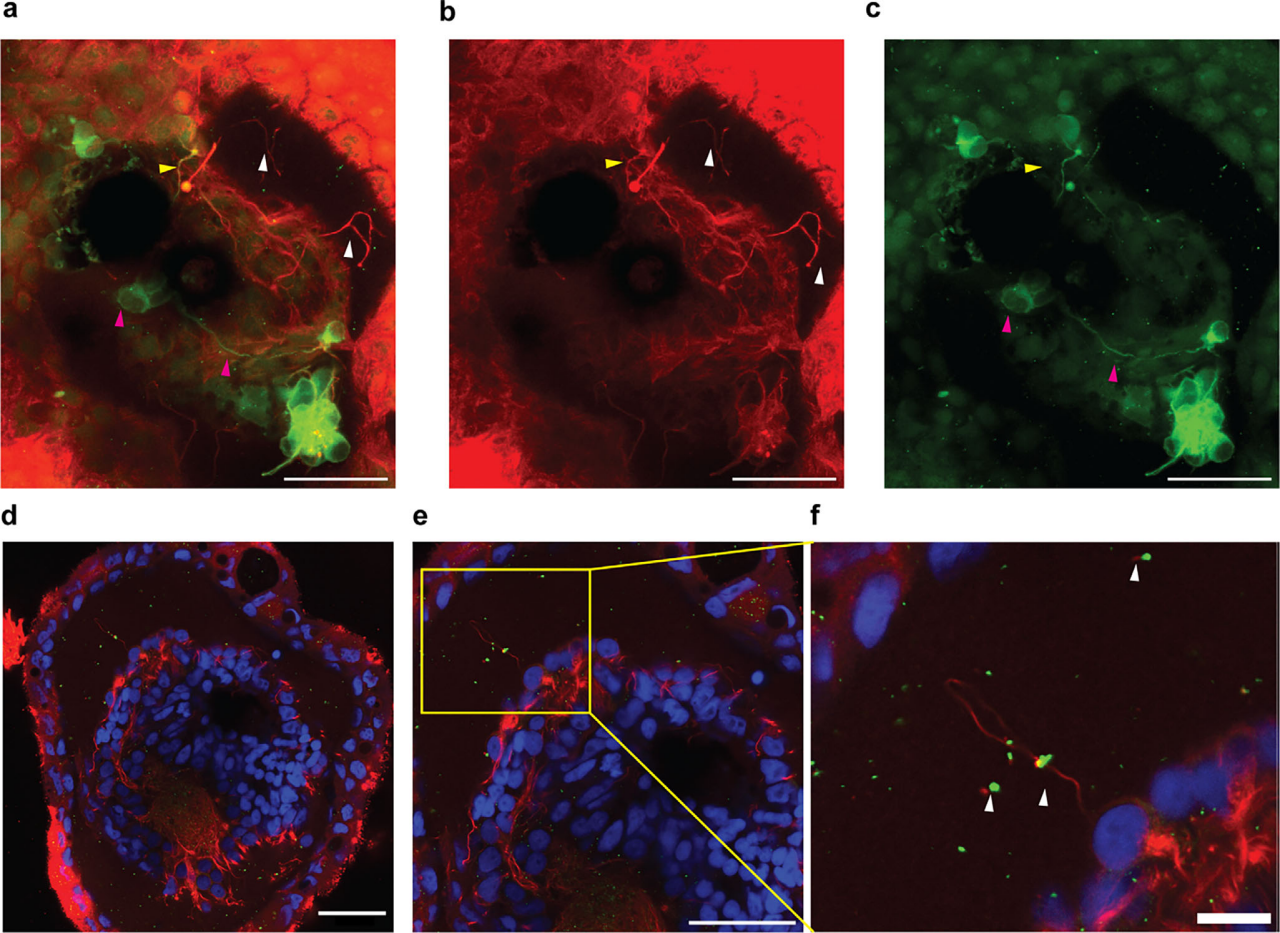
Immunostaining for acetylated α‐tubulin, MAP2, and synapsin‐1 reveals the presence of axons, dendrites, and synapses within neurobots. (a–c) Z‐projection of 11 confocal sections (total depth: 22 µm). Acetylated α‐tubulin (red) and MAP2 (green) are shown as: (a) merged image, (b) acetylated α‐tubulin channel, and (c) MAP2 channel. Yellow arrowheads mark regions of overlap; white arrowheads indicate sites labeled only for acetylated α‐tubulin; pink arrows denote regions labeled only for MAP2. Scale bar, 40 µm. (d–f) Distribution of putative synapses in a neurobot. Green puncta correspond to anti‐synapsin‐1 staining, red indicates acetylated α‐tubulin, and blue denotes nuclei. (e) Higher magnification of the neurobot shown in (d). (f) Enlarged view of the boxed region in (e). Scale bars, 40 µm (e) and 10 µm (f).

In a subset of neurobots, we performed co‐labeling with acetylated α‐tubulin and microtubule‐associated protein 2 (MAP2). MAP2 preferentially labels neuronal cell bodies and dendrites and is absent from axons [42], whereas acetylated α‐tubulin is most abundant in stable microtubules within proximal axons and present at lower levels in dendrites [43]. We observed clear MAP2 expression in neurobots, with distinct and partially overlapping labeling patterns between MAP2 and acetylated α‐tubulin. This differential distribution suggests that neurobots develop both axonal and dendritic compartments (Figure 3a–c).

To evaluate the presence of synaptic structures, we co‐labeled neurobots with an anti‐synapsin‐1 antibody, which serves as a presynaptic marker by labeling synaptic vesicles. Numerous synapsin‐1‐positive puncta were detected throughout the neurobots, frequently colocalizing with regions positive for acetylated α‐tubulin (Figure 3d–f, Secondary‐antibody‐only control: Figure S2). This overlap suggests that stable microtubule‐rich processes in the neurobots contain presynaptic specializations or vesicle clusters, consistent with the formation of putative synaptic contacts.

There was considerable variability in the pattern of neural growth among different neurobots. No two neurobots showed identical neural architecture (Figure S3). This is not surprising given the variability of initial conditions resulting from their manual construction and the inevitable variability of the amount of implanted tissue. This variability, however, allowed us to investigate correlations between various physical and behavioral characteristics of neurobots, which we will discuss in more detail in the following sections. Despite these variabilities, we found that neural processes in most neurobots tended to emanate from one or more nuclear regions (labeled using a nuclear stain), presumably corresponding to the implanted clumps of cells (Figure 1a and Figure 2c,d; Figure S3). Interestingly, these regions were often surrounded by regions with seemingly no nuclear staining (Figure 2b–d; Figure S3). The presence of these seemingly empty spaces is intriguing. We hypothesize that these regions may be acellular, may contain support structures such as the extracellular matrix (ECM), and/or may be occupied by neural processes that lack acetylated α‐tubulin, a marker of stable microtubules found in mature neurons but absent from immature ones. Support for the ECM hypothesis includes cases in which we observed neurites traversing long distances in this empty space along a straight line (Figure 2b, yellow arrow).

In order to test these hypotheses, we stained neurobots with phalloidin dye, which stains F‐actin filaments, which are present in all eukaryotic cells [44]. Additionally, we performed Second Harmonic Generation imaging to assess the presence of collagen fibers [45], which are the most common component of ECM. Although we found some evidence for collagen fibers and puncta, their expression was rather sparse (Figure S4b, green arrows; Figure S4e). This space may therefore contain other ECM proteins that do not form fibrils such as proteoglycans, laminins, or fibronectin [46]. Similarly, phalloidin staining was largely absent from this space and mostly overlapped with acetylated α‐tubulin, with only a few instances of differential labeling (Figure S4c,d). These findings suggest that the space is predominantly acellular but may still contain a small number of neural processes, consistent with the observed sparse labeling of acetylated α‐tubulin and synapsin‐1 within this region (Figure 3d–f). Future experiments are needed to further investigate the molecular composition of this space.

### 2.3 Neurobot Neurons Are Functional

**FIGURE 4:**
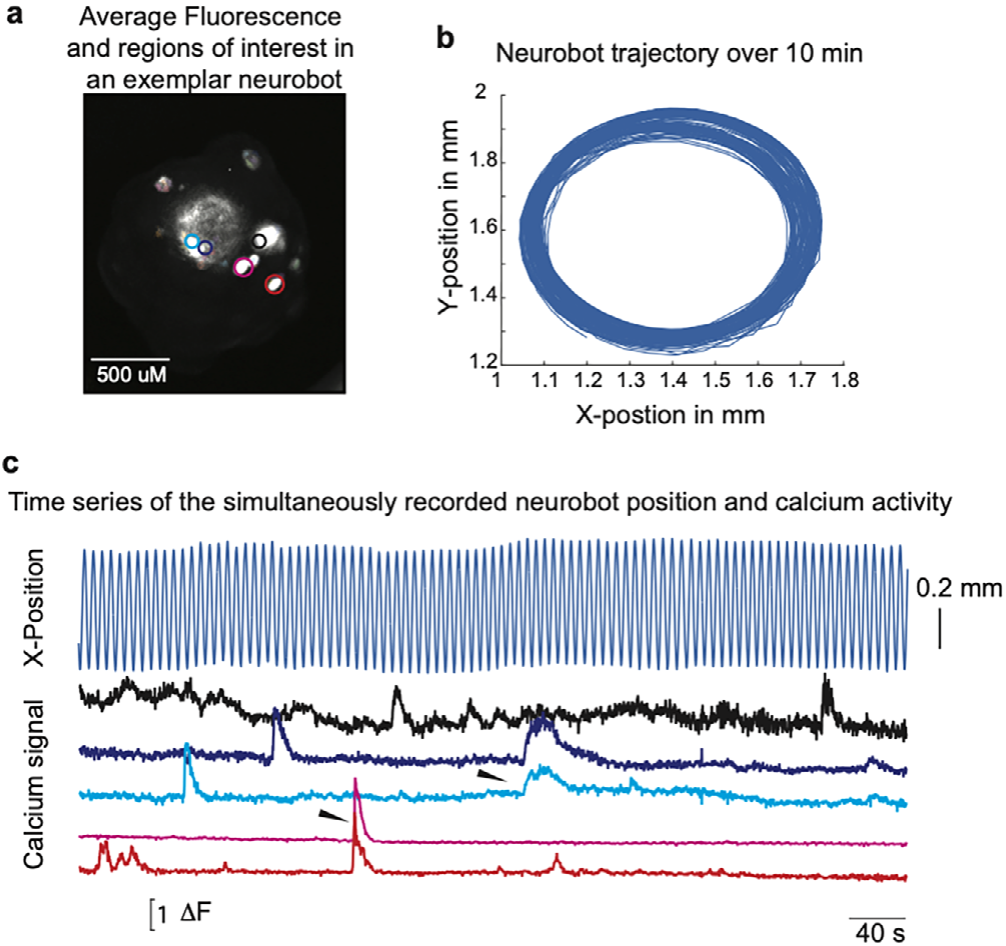
Calcium imaging in freely moving neurobots shows that the implanted cells are indeed active. (a) Average fluorescence of a freely moving neurobot containing neurons expressing GCaMP6s after motion correction (10 minutes of movement imaged at 5 frames per second). Colored circles correspond to regions of interest identified by the suite2p software, which could be single or multiple units. (b) Movement trajectory of the same neurobot. (c) The top curve shows the X‐position of the neurobot over time. The 5 bottom curves show baseline‐subtracted fluorescence activity of units labeled in panel (a). Arrowheads point to synchronized activity in some nearby (two shades of blue) and more distant (red and pink) ROIs.

To assess whether neurons within neurobots are functional, we built neurobots using neural cells extracted from embryos with genetically encoded calcium indicators (GCaMP6s, see Methods); this tool is commonly used to study neural activity [47]. We used a custom‐built widefield fluorescence microscope with a large field of view that enabled measurement of calcium activity in freely moving neurobots. Because the focal plane of the microscope was fixed in our setup, neural activity could only be recorded within a single plane of focus. It was therefore critical to maintain regions of interest within that focal plane, which proved challenging, particularly in neurobots exhibiting extensive rotational movements (See e.g., neurobot shown in Video S3). Neurobots with a flatter, disc‐like shape would be less likely to generate rotational movements and would therefore help maintain regions of interest in focus [48] (see Methods). Figure 4 shows an example of calcium signals recorded from a freely moving flattened neurobot, which exhibited circular movements around its center (Video S1). Motion‐corrected videos were then analyzed to extract the fluorescent activity (see Methods, Video S2). We found that the implanted cells indeed show calcium activity in all recorded neurobots. We occasionally observed synchronized activity in nearby or distant regions of interest (see e.g., arrowheads in Figure 4c), which may result from connectivity between these regions, although this could also be attributable to chance.

We found that keeping regions of interest in focus over longer periods of time was still quite challenging even in flattened neurobots. Further, although we did not formally study the impact of flattening on the movement patterns of neurobots, we found that they were in general less likely to move compared to regular ones. Consequently, we were unable to obtain repeated measurements of specific calcium activity sequences or movement patterns for statistical correlation analyses. Moreover, since every neurobot showed a different pattern of neural expression (Figure S3), findings in one neurobot could not be reproduced and confirmed in others. An important future direction will be to develop methods that enable consistent neural expression and experimental paradigms permitting repeated observation of directional movement and controlled rotation of regular, non‐flattened neurobots. Achieving these capabilities will be essential for rigorously linking neural activity to behavior.

### 2.4 Neurobots Tend to be More Active and Show an Increase in Their Movement Complexity, Compared to Non‐Neural Biobots

**FIGURE 5:**
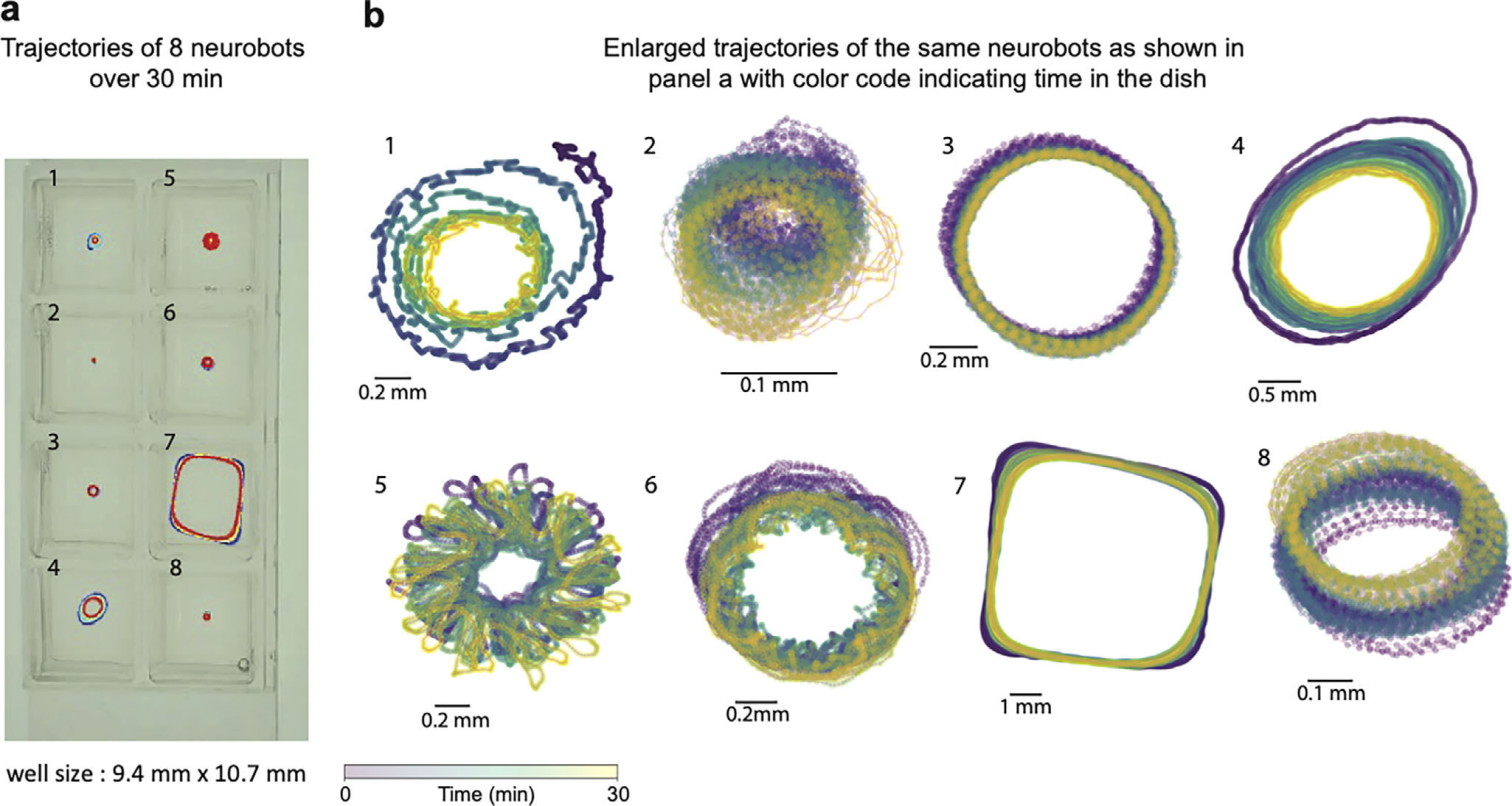
Neurobots show diverse and periodic patterns of spontaneous movement. (a) Exemplar trajectories of neurobots moving in an 8‐well plate over a 30‐minute trial. (b) Details of the trajectories of the same bots as shown in panel (b). The color gradient indicates time during the trial.

To investigate whether neural activity could influence bot movement, we recorded the spontaneously generated movements of both biobots and neurobots. We reasoned that if neurons exhibit spontaneous and variable activity patterns, as observed in our calcium imaging experiments, and if those neurons can modulate the bot's behavior, then neurobots should exhibit movement dynamics that differ from those of their non‐neural counterparts. We Recorded the spontaneous movements of the bots in small 8‐well plates over 30 minute periods (*n *= 46 neurobots and 48 biobots, Figure 5a, Video S3: single neurobot moving, Video S4: neurobots moving in an 8‐well plate). We then used an automatic tracking software [49] to measure the position of each bot at each video frame. Figure 5b shows the details of the 2D trajectories of the 8 neurobots depicted in panel (a). There was a large degree of variability in these trajectories, with some bots moving in circular/oval trajectories with relatively constant diameter (Figure 5b, bots #3, #8); bots that followed circular trajectories varying in diameter over time (Figure 5b, bot#4); ones that made more complex, sometimes spirograph‐like patterns (Figure 5b, bots #1, #5, #6); those that were seemingly following the dish's boundaries (Figure 5b, bot #7); bots that circled over very small areas (Figure 5b, bot #2); and those that did not move at all (data not shown). Interestingly, all moving bots tended to exhibit repeating behavioral motifs.

With the positional information obtained from tracking, we used custom Python code to extract 4 kinematic parameters from the bots to compare neurobots with biobots. These were the total distance travelled, average speed, average acceleration, and the percentage of the well that was traversed in 30 minutes (see Methods). We found no significant difference in the total distance traveled, the percentage of the well covered, and average speed and acceleration (Figure S5). However, we found that the minimum movement speed of neurobots was significantly higher than that of biobots, indicating that neurobots tended to move more than biobots, remaining idle less often.

**FIGURE 6:**
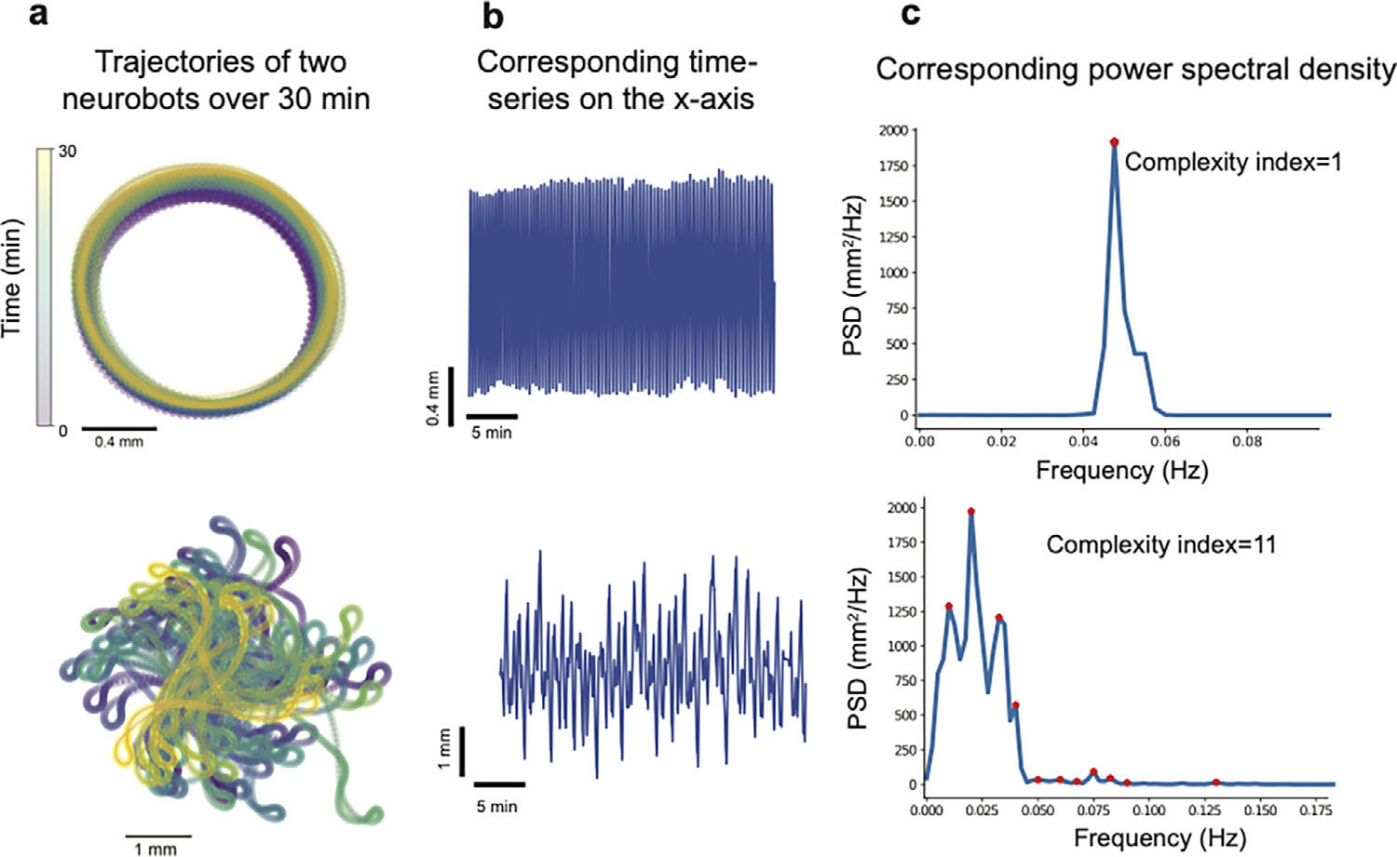
The number of peaks in the power spectral density was used to quantify the complexity of movement trajectories. (a) Examples of simple (top panel) and complex (bottom) trajectories. (b) Time series of the movement amplitudes projected on the *x*‐axis. (c) Power spectral densities corresponding to time series in panel (b) Red stars mark the location of significant peaks.

We had observed that some bots show simpler circular movement trajectories with relatively constant radii (e.g., Figure 5b‐bot #3), whereas others showed changes in their circling radius (e.g., Figure 5b‐bot #4) or a generally more complex movement pattern (e.g., Figure 5‐ bot #5). More complex movement patterns in neurobots would support the hypothesis that neural activity may play a modulatory role in their spontaneously generated movements. To further investigate potential differences in the movement patterns of neurobots and their non‐neural counterparts, we used a spectral analysis, calculating the Welch power spectral density (PSD) of the trajectory time series along the *x* and *y* coordinates (Figure 6a,b, two exemplar trajectories and their corresponding time series on the *x*‐coordinate, see Methods). We then detected significant peaks in the *x* and *y* PSDs, summed the number of unique peaks in *x* and *y* PSDs, and defined this number as the Complexity Index (CI, Figure 6c). In this analysis, a CI of one indicates a circular trajectory with constant diameter (where both *x* and *y* PSD have one peak at the same location), and the number increases as the trajectory becomes more complex. A complexity index of zero signifies non‐moving bots. We further used a time‐frequency analysis (wavelet‐transform) and surrogate data to confirm the significance of the power at the peaks identified in the Welch analysis (see Methods).

**FIGURE 7:**
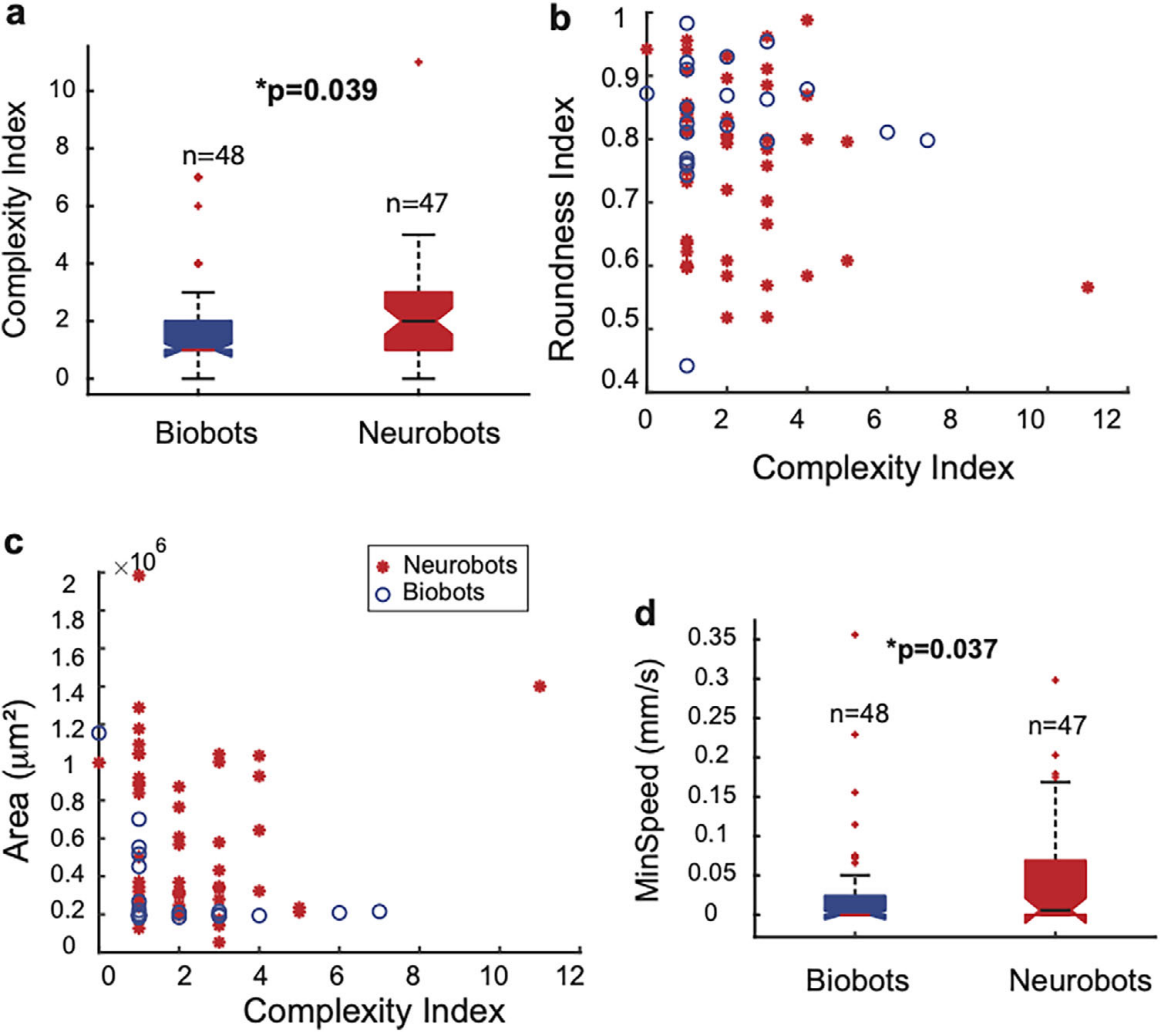
Neurobots show differences in movement patterns compared to biobots. (a) Neurobots have more complex trajectories than biobots (Kruskal–Wallis test, *p* = 0.039), and (b) this complexity is not correlated with their roundness (Pearson correlation coefficient = −0.15, two tailed *t*‐test *t*(45) = 45, *p* = 0.2), or (c) their area (Pearson correlation coefficient = −0.03, two tailed *t*‐test *t*(df) = 45, *p* = 0.76). (d) Neurobots were more likely to be active (have non‐zero minimum speed) than biobots (Kruskal–Wallis test, *p* = 0.037).

Interestingly, we found that neurobots showed a significantly higher degree of trajectory complexity compared to biobots (Figure 7a). This increased complexity could not be explained by the roundness or size of the biobots and neurobots, as these variables were not significantly correlated (Figure 7b,c). The increased complexity of neurobots could be a result of increased variability in the beating frequency of cilia in MCCs, changes in their spatial distribution, changes in the 3D structure of the bot, or changes in the activity or distribution of other cell types (including neurons) that may modulate the ciliary beating frequency, among other factors. When we measured this index in sham neurobots, we also found an increase, albeit not significant, relative to biobots (Figure S1c), indicating that the increased complexity we observe in neurobots is at least partially due to factors other than neural signaling. Like biobots, sham neurobots were more likely to show longer periods of immobility, and their minimum speeds were not significantly different from those of biobots (Figure S1d). Finally, consistent with the finding that minimum speed was significantly higher in neurobots (Figure 7d), we found that most inactive bots (Npeaks = 0) were in the biobot category, with 6 out of 48 (12.5%) biobots inactive, whereas only one out of 47 (2.1%) neurobots was inactive.

### 2.5 A Seizure‐Inducing Drug Differentially Affects the Behavior of Neurobots and Biobots

**FIGURE 8:**
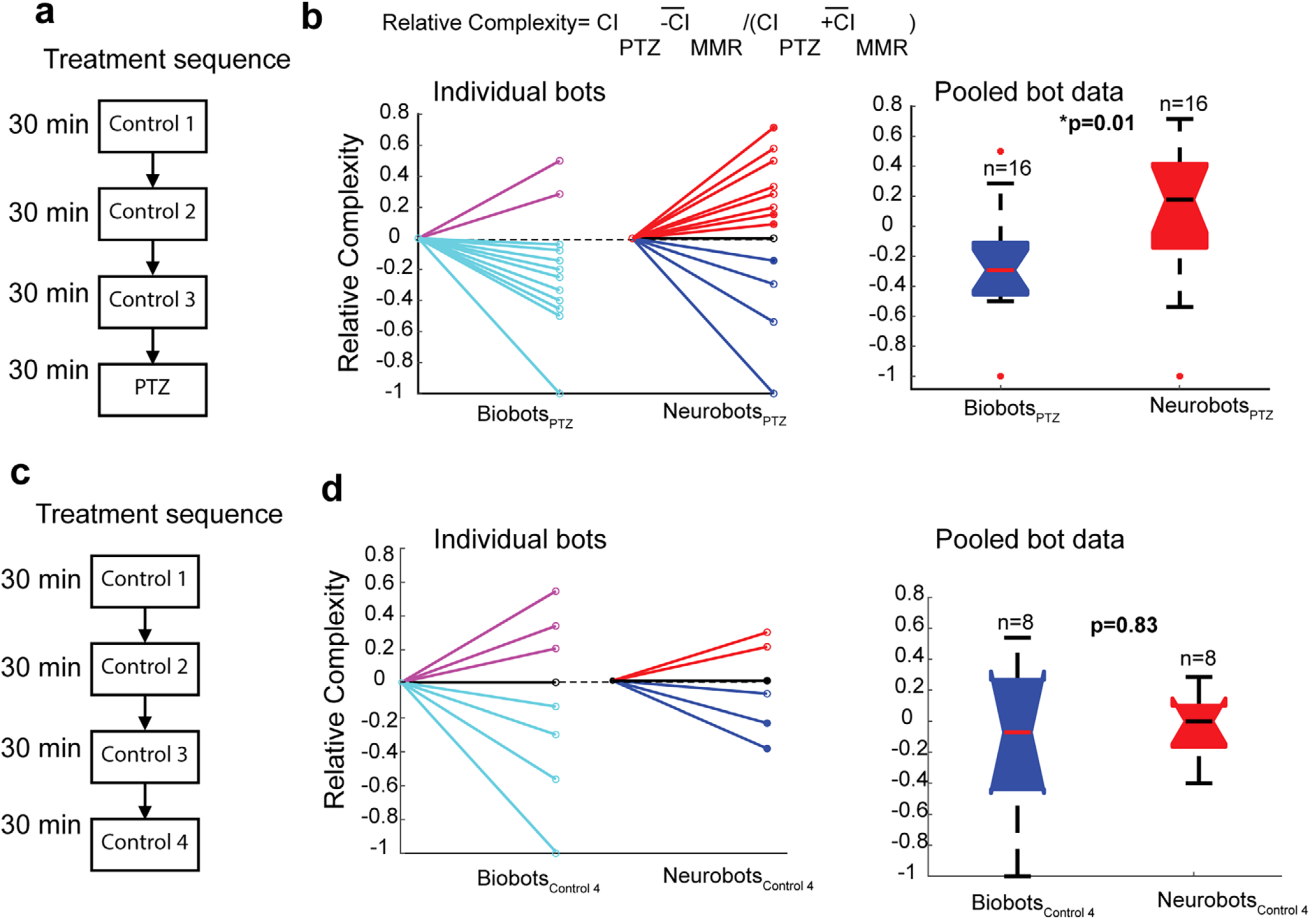
PTZ differentially impacted the movement of neurobots and biobots. (a) Experimental protocol for testing the effect of PTZ on the movement of neurobots (*n* = 16) and biobots (*n* = 16). Movement of bots was measured in control media, across three transfers for 30 min. The bots were then transferred to a dish containing 15 mM PTZ solution, and their behavior was measured for another 30 min. (b) Relative complexity was defined as the complexity index measured while in PTZ, relative to the average complexity index measured for three consecutive controls. Although most biobots reduced their complexity index relative to control (cyan lines: decreased complexity, purple lines: increased complexity), the relative complexity index for neurobots was equally likely to increase or decrease (red and blue lines). The relative complexity of zero corresponds to the average CI for the three controls (black). Filled circles indicate neurobots cultured in zolmitriptan prior to testing. Neurobots showed significantly higher relative complexity compared to biobots (Kruskal–Wallis test, *p* = 0.01). (c,d) Show the results from control experiments where the PTZ treatment block was replaced with another control step. Trajectory complexity was equally likely to increase or decrease in the absence of PTZ. There was no significant difference between the relative complexity of biobots and neurobots (Kruskal–Wallis test, *p* = 0.01).

To further investigate whether neural activity could play a role in modulating trajectory complexity, we performed pharmacological experiments, treating groups of neurobots and biobots with entylenetetrazole (PTZ), a GABA_A_ receptor antagonist used for its seizure‐inducing effects in animal studies [50]. Although we do not know the identity of neuronal constituents of neurobots, we reasoned that a positive result, for example, an increase in the complexity index after PTZ treatment, could be an indirect indication of the presence of GABAergic control of movement. To test this, we performed experiments in which we video recorded the movement of neurobots and biobots in regular media and compared the complexity indices after they were transferred to dishes containing 15 mM PTZ. To get an estimate on the baseline complexity index and account for potential variability due the transferring, we followed the experimental protocol depicted in Figure 8a.

The behavior of bots (16 neurobots and 16 biobots) was video recorded for 30 minutes in 8‐well plates filled with regular media (control 1), after which the bots were transferred to the second and third sets of dishes containing regular media (control 2, control 3). The bots were then transferred to dishes containing PTZ (Figure 8a). For each bot, we calculated the CIs for the three control conditions and used the average to calculate relative complexity measures for PTZ and the wash. To control for non‐specific effects that may occur upon the fourth transfer, we performed the same sequence of transfers for a second set of bots (8 neurobots and 8 biobots), where the fourth transfer was into wells that contained regular media and not PTZ (Figure 8c).

We found that all except two biobots showed a relative decline in their movement complexity while in PTZ (Figure 8b), thereby significantly reducing the CI relative to control (one‐sample *t*‐test, *t*(df) = 15, *p* = 0.009). The effect of PTZ on biobots suggests that PTZ may act on GABA receptors expressed by non‐neuronal cells or exert other off‐target effects. Indeed, GABAergic receptors are present on goblet cells lining the surface of both biobots and neurobots, which could indirectly modulate movement through altered mucus secretion (see Discussion).

The majority of neurobots, on the other hand, showed increased complexity relative to control, although a few did show a decline (Figure 8b, left panel). As a result of this dichotomy in the impact of PTZ, the average CI in neurobots was not significantly different relative to control (one‐sample *t‐*test, *t*(df) = 15 *p *= 0.39). When we compared the relative complexity after PTZ treatment, however, we found a significant difference between neurobots and biobots, with neurobots showing significantly higher values for relative complexity (Figure 8b, right panel). This effect was not observed when we transferred bots to the control media a fourth time (Figure 8c,d), confirming that the observed effect was specific to PTZ. The variability of the impact of PTZ on neurobots is in fact not surprising given the potential variability in the identity and the degree of expression of neurons (Figure S3). What is very interesting is the significant differential impact on the biobots and neurobots. Because neurobots are biobots + neurons, the fact that most of them showed an increase in their CI suggests a role for neural activity acting against the default inhibitory effect of PTZ on the movement of biobots. Alternatively, neural expression could indirectly contribute to these effects through its impact on the expression of MCCs or other cell types present on the outer surface of the bots.

### 2.6 Neurobots Show Significant Differences in the Distribution of MCCs, and Their Roundness Is Anti‐Correlated With the Degree of Neural Expression

**FIGURE 9:**
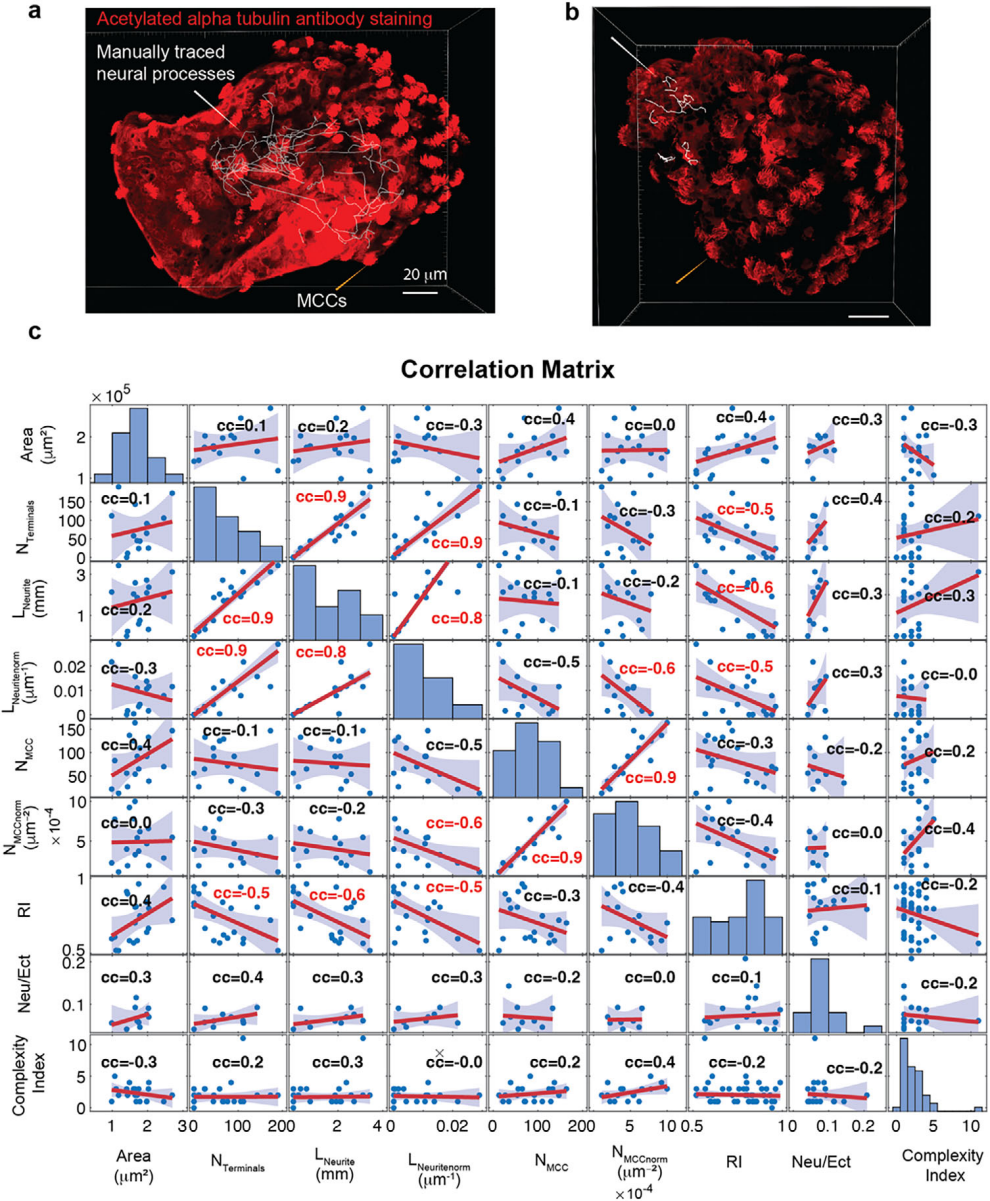
Pair‐wise correlations between size, shape, neural expression, and movement complexity in neurobots. (a,b) Examples of neurobots stained with acetylated α‐ tubulin which labels multiciliated cells and neurons. Overlaid white curves show the neural processes traced using Imaris software. Orange arrow heads point to exemplar multiciliated cells (a) Example of a neurobot with a high degree of innervation *N*_terminals _= 327, *L*_Neurite _= 7635.9 mm. (b) Example of a neurobot with small degree of innervation *N*_terminals _= 40, *L*_Neurite _= 753.8 mm. (c) Pairwise correlation between structural parameters of all stained neurobots and Complexity Index, *N*_terminals_ = total number of endings, *L*_Neurite _= total length of neurites, *L*_Neuritenorm _= total length of neurites normalized to area, *N*_MCC _= total number of multiciliated cells on the surface, *N*_MCCnorm _= *N*_MCC_ normalized to area, RI = Roundness Index, Neu/Ect = ratio of the areas of neural implant to ectoderm shell. White and yellow arrows point to the position of manually traced neural processes and multiciliated cells. Pearson correlation coefficients are depicted for each pair. Values in red correspond to statistically significant correlations (two‐tailed Student's *t‐*test, *P *< 0.05). A robust linear regression was used for linear fits. The full model statistics are provided in Spreadsheet S1).

To quantify the overall amount of neural expression and its relationship with neurobot morphology and behavior, we used the confocal images of neurobots immunostained with acetylated α‐tubulin antibody, which labels neurons and cilia in the MCCs. Using this data, we traced the neural processes and quantified the position of the MCCs using Imaris software (Figure 9a,b, see Methods). In this analysis, we did not distinguish between axons or dendrites and could not determine whether these processes belonged to individual neurons. We could, however, obtain rough estimates of the total amount of neural tissue and the degree of branching by calculating the total length of neurites and the number of terminals, which were defined as the number of nerve endings, agnostic of their identity (i.e., axonal or dendritic). Figure 9 (top panels) shows two examples of such traces in cases of neurobots with very few and many neurites. We calculated the correlation between the degree of neural growth, expression of MCCs, bot size, and shape, as well as the CI of the trajectories across all neurobots for which we had both behavioral and structural data (Figure 9c). We also calculated the correlation of all these parameters with the relative amount of neural tissue that was implanted on Day 1 as the neurobots were constructed. This ratio was calculated by dividing the area of the implanted clumps by that of the external shell (Figure 9c: Neu/Ect, Figure 1a (ii), see Methods).

Based on this analysis, we found that the total number of neuron terminals was highly correlated with both absolute (Pearson correlation coefficient = 0.9, *t*‐test, *t*(df) = 20, *p* = 2.63e‐09) and area‐normalized neural length (neurite density, Pearson correlation coefficient = 0.9, *t*‐test, *t*(df) = 17, *p* = 2.77e‐06). Interestingly, we found a significant negative correlation between neurite density and MCC expression density (Pearson correlation coefficient = −0.6, *t*‐test, *t*(df) = 12, *p *= 3.33e‐02); neurobots with higher neurite density tended to have lower overall density of MCCs. Consistent with this finding, we found that biobots (which do not have neurons) have a significantly higher density of MCCs compared to neurobots (Figure S6, Kruskal–Wallis test, *p* = 0.027). Additionally, we found a significant negative correlation between the Roundness Index (RI) and neurites’ absolute length (Pearson correlation coefficient = −0.6, *t‐*test, *t*(df) = 20, *p* = 5.09e‐03), neurites’ normalized length (Pearson correlation coefficient = −0.5, *t*‐test, *t*(df) = 17, *p* = 1.72e‐02), and total number of terminals (Pearson correlation coefficient = −0.5, *t*‐test, *t*(df) = 20, *p* = 1.72e‐02). That is, the more elongated the bot, the more neural expression, suggesting that the elongation could be a result of neural processes growing within the neurobot. This hypothesis is consistent with the finding that sham neurobots were not different from biobots in their RI (Figure S1a, Kruskal–Wallis test, *p* = 0.89). We did not find a significant correlation between CI and neural expression metrics although the neurobot with the highest complexity index also had the largest number of terminals and neurite length (see the outlier data point in the panels). Similarly, we found only a small correlation (non‐significant) between the relative amount of implanted tissue and the degree of neural expression metrics.

Previous studies showed that treatment with zolmitriptan, which is a selective 5‐hydroxytryptamine (5‐HT) 1B/1D receptor agonist, increased the degree of ectopic (but not native) neural growth in *Xenopus* embryos [51]. We investigated whether this treatment would have an impact on the degree of neural growth in neurobots, where the neurons are all ectopic. Interestingly, we found that this treatment increased the degree of neural expression in neurobots as well, although it did not have a significant effect on any of the behavioral measurements including trajectory complexity (Figure S7). In this group, we found a tight correlation between the ratio of neural implant to ectoderm shell and total number of terminals, as well as total neural length (Figure S7). These results indicate that neurons in a neurobot behave as ectopic, not native, cells. Notably, 4 of the 16 neurobots in the PTZ study presented in the previous section were cultured in zolmitriptan (filled circles Figure 8b; Figure S7), three of which showed an increase in relative complexity. This result points to the possibility that this treatment may bias neural expression toward those that respond to PTZ that is, GABAergic neurons. Further experiments are required to characterize the impact of zolmitriptan treatment on the neural expression patterns within neurobots.

In summary, neural growth in neurobots significantly impacts their shape and the distribution of MCCs, and this growth could be potentially increased by modulating serotonergic signaling. The degree to which neural expression contributes to trajectory complexity remains elusive. The lack of observed correlation between neurite growth and complexity index points to the potential heterogeneities in cell type expression and connectivity profiles of neurites across neurobots.

### 2.7 The Three Types of Bots Exhibit Significantly Different Patterns of Gene Expression

Like the structure and function of the central nervous system, transcriptomes are typically viewed as products of a long evolutionary history of selection. In standard organisms, they are further shaped by ongoing neural inputs [52, 53]. What would the transcriptome of a novel construct with a nervous system look like? With this question in mind, we next asked what changes to default biobot transcriptomes, if any, would be induced by the presence of neural tissue. To characterize the transcriptome of neurobots and compare it to its non‐neuronal counterparts (i.e., biobots and sham neurobots), we performed bulk RNA sequencing of their tissue. For each bot type, four biological samples were included (neurobots: NB1‐4, biobots: BB1‐4, sham neurobots: SH1‐4). Due to the small size of the bots, and, therefore, the small amount of RNA, each sample comprised tissue from multiple bots (see Methods).

**FIGURE 10:**
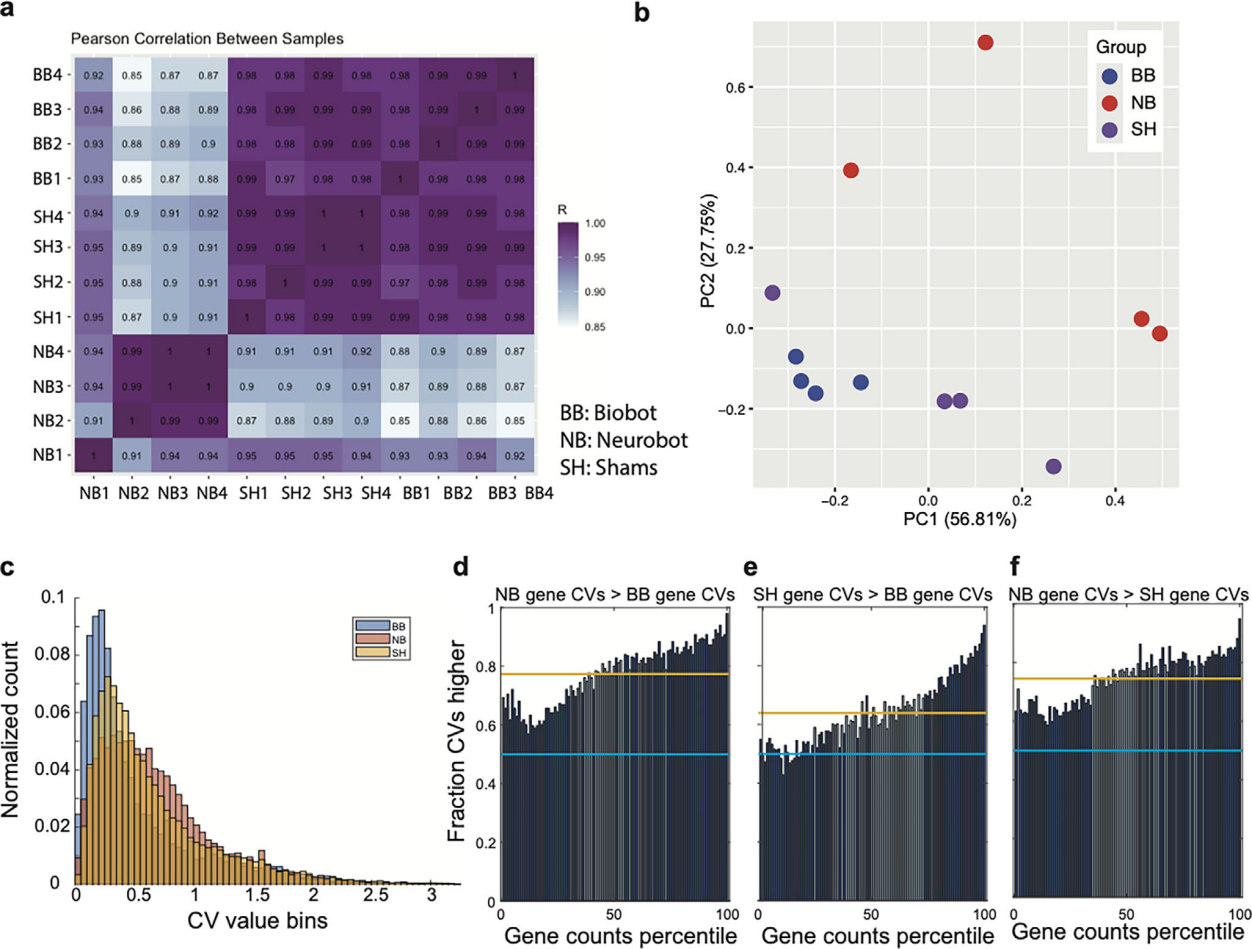
Comparison of gene expression and its variability between neurobots, biobots, and sham neurobots. (a) Pearson correlation of gene expression within and across groups of neurobots (NB), sham neurobots (SH), and biobots (BB). The closer the value to 1, the more similar the expression patterns. (b) Principal component analysis of gene expression values. Each dot corresponds to one sample, which contained multiple bots of one kind. (c) Histograms of coefficients of variation (CV) in gene counts (FPKM) in neurobots (red), biobots (blue), and sham neurobots (yellow). Neurobots showed a significantly higher variability in their gene counts compared to both biobots and shams, and shams showed a higher variability compared to biobots (Kruskal–Wallis test with multiple comparisons using Tukey's honestly significant difference test; *p* < 0.00001). Genes with higher counts showed a higher degree of variability in their expression when comparing neurobots with biobots and shams (d,e). Dark blue bars mark the bins where the difference in CV was significantly different from what is expected if the ranking of genes were randomly shuffled. Similarly, genes with low levels of expression showed lower coefficient of variation than expected by chance. The *p*‐value of each bin was defined as the proportion of the bin fractions from the distribution that were further in absolute value from the distribution mean than the true bin fraction. Bins with *p*‐values of *p *< 0.05 were deemed statistically significant and were colored dark blue. All other bins were colored light blue. The mean bin value from the distributions was plotted as an orange line. (f) Same as (d,e) but comparing neurobots with shams. Bin values above 0.5 (light blue line) indicate that more than half of the CVs in that bin had higher values in that comparison.

We found a high degree of correlation between normalized gene expression levels among all samples within each group (Fragments Per Kilobase of transcript sequence per Millions base pairs sequenced, FPKM [54]), indicating reliability and repeatability of the results (Figure 10a). Moreover, we found that gene expression levels in biobots and sham neurobots were much more correlated to one another than to neurobots (Figure 10a, see Methods). Similarly, neurobots could clearly be separated from shams and biobots based on the principal component analysis on the normalized gene expression value (FPKM) of all samples (Figure 10b, see Methods).

We next compared the gene count variability across the samples of biobots, neurobots, and sham neurobots on a gene‐by‐gene basis (Figure 10c–f). We found that neurobots showed a significantly higher variability, quantified by coefficient of variation (CV, see Methods) in their normalized gene counts (FPKM) across neurobot samples, compared to both biobots and shams, and samples of sham neurobots showed a higher variability compared to samples of biobots (Figure 10c). We then compared pairs of groups (e.g., NB and BB) to determine the fraction of genes that had a greater CV in normalized gene count in one group than the same gene in the other group. For the chosen pair of groups, genes were ranked by the mean count value across all pools of both groups, and the CV of each gene's counts across the pools of each group was calculated (Figure S10, see Methods). The CV list was split into 100 bins (percentiles) containing an equal number of genes, and the fraction of genes in the bin for which the CV of the first group was greater than that of the second group was found and plotted.

We found that in all bins, more than half of the neurobot genes showed higher CV compared to the biobot and sham group (Figure 10d,f; cyan horizontal line is at 0.5). For the sham neurobot group, genes in most, but not all bins showed higher CV compared to biobots (Figure 10c). In addition, we found a trend for genes with higher normalized counts showing a higher degree of variability in their expression when comparing neurobots with biobots and shams (Figure 10d,f). This pattern was significantly different from what is expected from chance in most bins (dark blue bins), that is, relative to the average CV calculated across all bins regardless of the order (orange line, see Methods). The overall higher variability seen in neurobots and shams compared to biobots could be due to several factors. First, in both neurobots and shams, the implanted cells are harvested from ∼50 embryos, whereas a biobot is made out of a single embryo. Moreover, higher variability in the implanted bots is expected due to the high variability in the size of the implants in both neurobots and shams. However, these are likely not the only factors involved, as neurobots showed significant differences in their gene count variability compared to shams. Neural differentiation, therefore, likely plays an important role in the increased variability in gene counts seen in neurobots.

**FIGURE 11:**
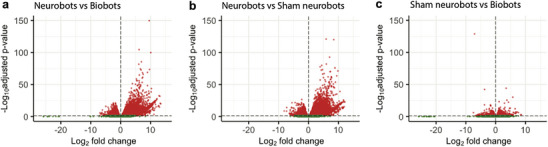
Distribution of differentially expressed genes between different bot groups. (a–c). The *X*‐axis shows the fold change in gene expression between samples of different groups, and the *Y‐*axis shows the statistical significance of the difference. Red dots represent genes that were significantly up (positive values on the *X*‐axis) or downregulated (negative values on the *X*‐axis); green dots represent genes with no significant change. Statistics were evaluated by DESeq2, which employs the two‐sided Wald test. Multiple hypothesis testing corrections were used to obtain adjusted *p*‐values. Red circles represent genes with adjusted *p‐*values that were statistically significant (*p* < 0.05).

Additionally, we found that neurobots included a significantly larger number of genes that were differentially expressed relative to biobots and sham neurobots (Figure 11a,b), whereas biobots exhibited a smaller subset of differentially expressed genes relative to sham neurobots (Figure 11c). Moreover, the number of significantly upregulated genes (*p* < 0.05, red dots with positive fold change) in neurobots compared to biobots and shams (6774 and 6859 genes, respectively), were much higher than those that were significantly downregulated (red dots with negative log fold change, 3578 in neurobots vs biobots and 4010 in neurobots vs shams), resulting in highly asymmetric volcano plots (Figure 11a,b). This was not the case when comparing shams with biobots (Figure 11c, 1733 upregulated and 1429 downregulated genes). These results are consistent with a gain‐of‐function as a result of neural growth in neurobots.

**FIGURE 12:**
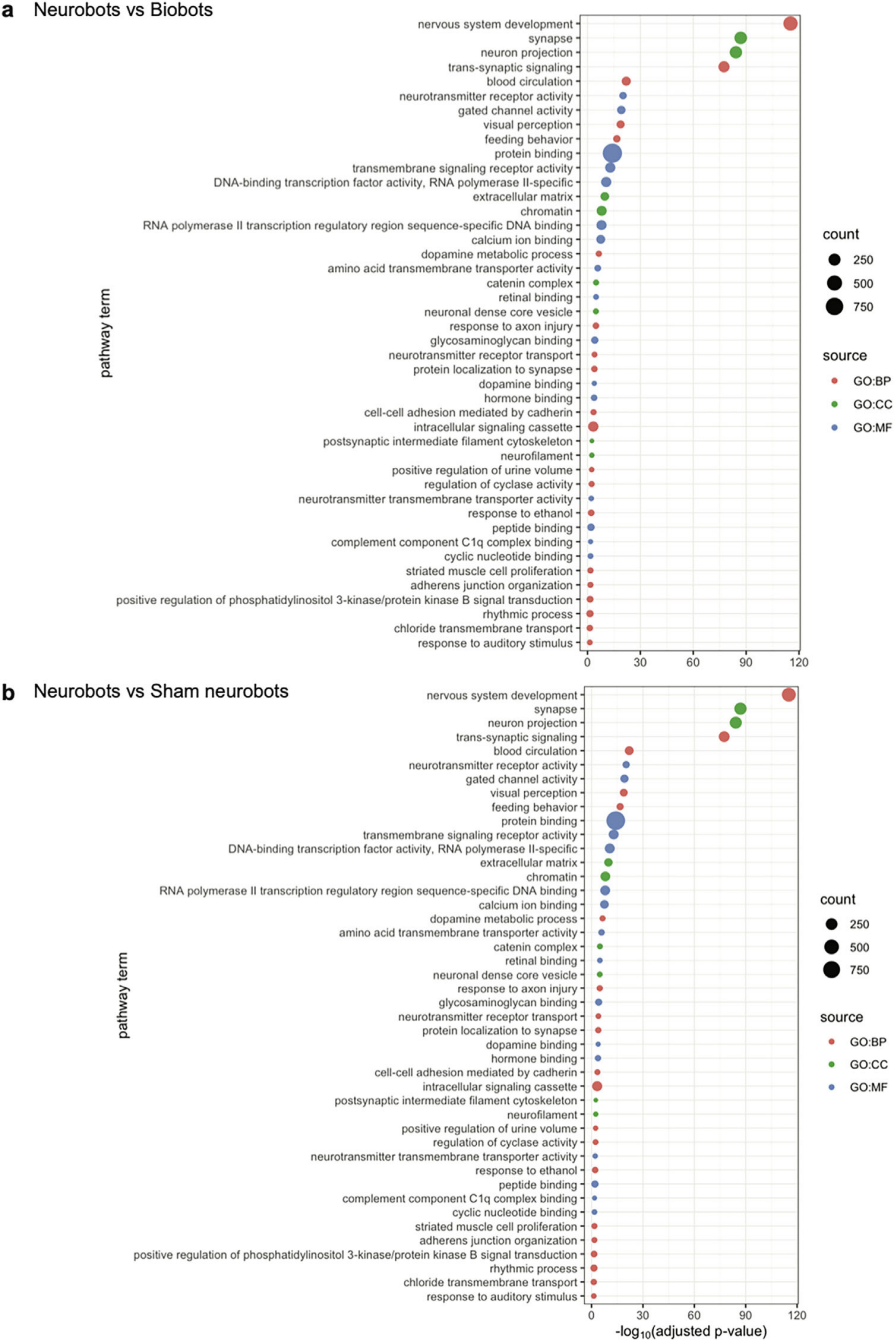
Enrichment analysis performed using Gene Ontology annotations on differentially expressed genes with at least 4‐log fold upregulation in expression. (a) neurobots versus biobots (b) neurobots versus shams, BP: Biological processes, CC: Cellular Components, MF: Molecular Function.

We next investigated which biological functions or pathways are significantly associated with the differentially expressed genes. We used Gene Ontology (GO) enrichment analysis to annotate genes to biological processes (bp), molecular function (mf), and cellular components (cc). Due to the large number of upregulated genes in neurobots, we focused on the highly overexpressed genes for this analysis (4 log‐fold or more increase in expression, 2445 genes when comparing neurobots to biobots and 2026 genes when comparing neurobots to shams). The most significantly upregulated pathways in neurobots relative to biobots, as well as in neurobots relative to shams, related to nervous system development, and synapse and neuron projection (Figure 12a,b). One of the interesting genes we found up‐regulated specifically in neurobots relative to biobots and shams is *Dact‐4*, a member of an evolutionarily conserved family of Dishevelled‐binding proteins involved in the regulation of Wnt and TGF‐beta signaling which is expressed in the Spemann organizer [55]. This suggests that the presence of neurons might exert an organizational influence on the surrounding soma; this molecular signature is consistent with known roles of the nervous system to direct cell behavior in cancer suppression [56, 57, 58], regeneration [59], and embryonic morphogenesis [53, 60]. Trans‐synaptic signaling and neurotransmitter receptor activity were also significantly upregulated in neurobots. This included glutamatergic, GABAergic, cholinergic, dopaminergic, serotonergic, and glycinergic receptors. Surprisingly, exclusively in neurobots, we also found significant enrichment in pathways involved in visual perception (Figure 12a).

Although pathways relating to neuron projection and trans‐synaptic signaling were slightly upregulated in sham neurobots compared to biobots, there were far fewer genes in each pathway, and they were less significant in their degree of upregulation compared to those in neurobots (Figure S8a). Further, there were relatively fewer genes downregulated when comparing neurobots with biobots, and shams (63 and 116 genes respectively), and very few genes were significantly downregulated by 4‐log folds when comparing shams with biobots (38 genes). Our enrichment analysis showed that the largest group of downregulated genes in neurobots compared to biobots belonged to the cellular component pathway (extracellular region in Figure S8b). Interestingly, this pathway includes some of the genes expressed in the *Xenopus* skin, including glycoprotein 2 (*gp2*), and mucin (*muc17*) suggesting that the properties of the “skin” of neurobots might be different from those of biobots.

In order to identify functional biological modules of differentially expressed genes, we extracted the largest protein–protein‐interaction (PPI) sub‐network of these genes using the STRING database. We then performed network embedding and clustering using multi‐nonnegative matrix factorization (MNMF) [61] to find specific functional biological modules. The clusters were subsequently enriched using g: Profiler [62]. Based on this analysis, we identified 25 clusters for neurobots vs biobots comparison and 5 clusters in neurobots vs sham neurobots comparison (Spreadsheet S2, Figures S9 and S10). There were not enough upregulated genes between biobots and sham neurobots to allow for this analysis. Similarly, due to the low number of downregulated genes, the network analysis could not be performed at either a 4‐fold or a 2‐fold change threshold level.

Consistent with the findings from the enrichment analysis, we found clusters containing genes critical for the development of the nervous system, cell fate commitment, and *wnt* signaling pathways (Cluster 3, Figure S9a, see the NB vs BB tab in Spreadsheet S2). A plethora of growth factors (e.g., various FGFs, BDNF, and EGF), and their receptors were revealed by Cluster 17, which also showed enrichment in enzyme‐linked receptor protein signaling pathways (Figure S9b, see the NB versus BB tab in Spreadsheet S2).

**FIGURE 13:**
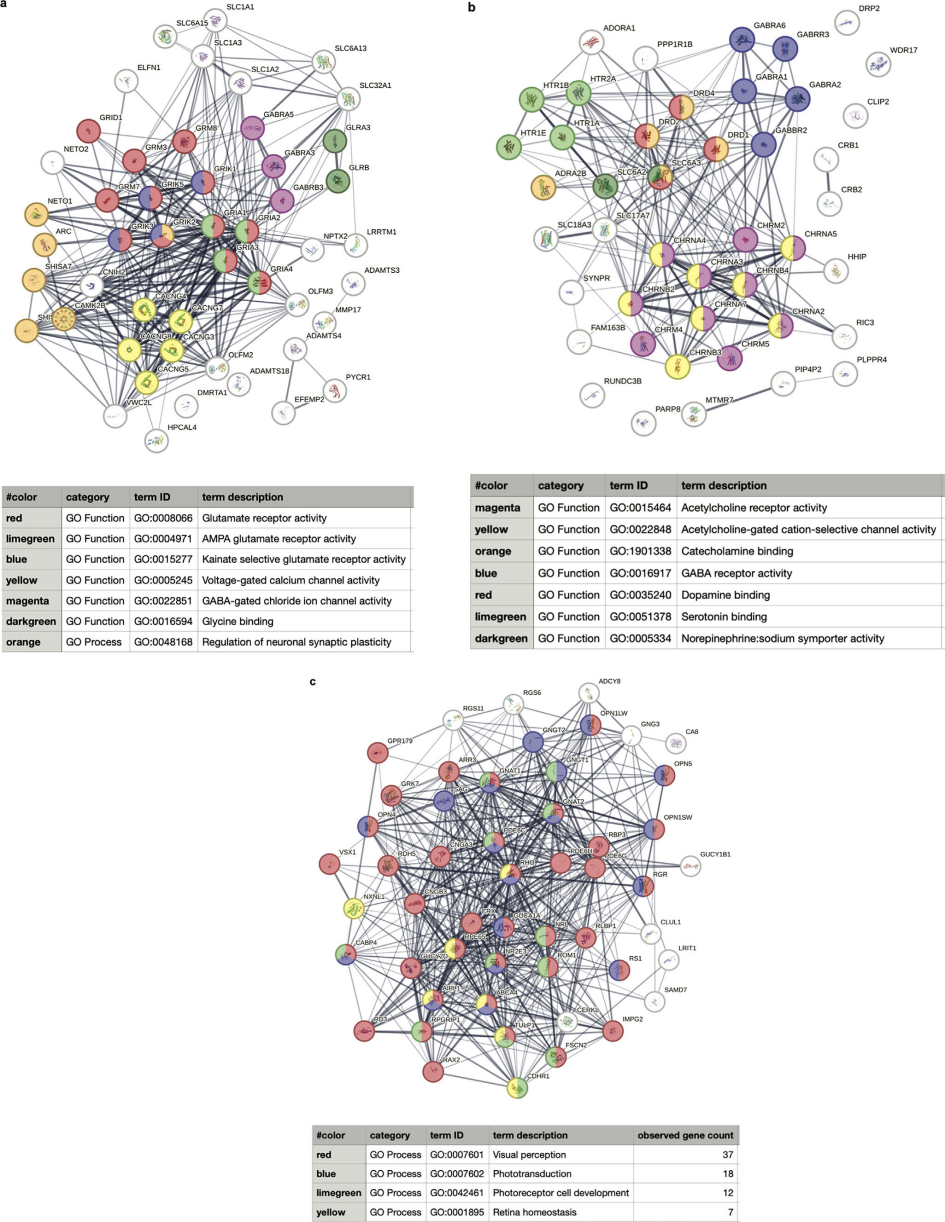
Cluster‐based network connectivity patterns among genes that were upregulated in neurobots compared to biobots. (a) Cluster 15 (b) Cluster 23 (c) Cluster 1. Network connectivity was calculated using the STRING online tool. The edges indicate both functional and physical protein associations the line thickness indicates the strength of data support. Only nodes with interaction scores with confidence higher than 0.4 are shown. Nodes of special interest are highlighted in color.

Neurobots contained genes encoding various neurotransmitter receptors including glutamate (e.g., *gria1‐4*), kainate receptors (e.g., *grik1,2,3,5*), GABAergic (e.g., *gabara3,5; gabarb3*), and glycinergic receptors (e.g., *glra3; glrb*), genes encoding voltage gated calcium channels (*cacng3,4,5,7,8*), as well as those involved in the uptake of neurotransmitters (e.g., *slc1a1,2,3*). Genes with important roles in synaptic plasticity were also present in neurobots (*arc, camk2b*, Cluster 15, Figure 13, see the NB vs. BB tab in Spreadsheet S2) [52]. Cholinergic and muscarinic (*chrna2,3,4,5,7; chrnb2,3,4, chrm2,4,5*), serotonergic (*htr1a,b,e*; *htr2a*), and dopaminergic (*drd1,2,4*) were among other neurotransmitter receptors (Cluster 23, Figure 13b, see the NB vs BB tab in Spreadsheet S2).

Notably, one of the largest clusters (Cluster 1) contained genes encoding various aspects of visual perception, phototransduction, and photoreceptor development (Figure 13c, see the NB vs. BB tab in Spreadsheet S2). Specifically, this cluster included red and violet cone opsins (*opn1lw, opn1sw*), retinal G‐protein couple receptors (*rgr*), melanopsin (*opn4, opn5*), rhodopsin (*rho*), as well as many other related genes that encode proteins involved in visual processing. In addition to Cluster 1, Cluster 12 (Figure S9c) also included genes related to eye, lens, and retina development, including genes found in major retinal cell types [63], that is, retinal ganglion cells (*neurod1,2; pou4f1*), and horizontal cells (*onecut1, lhx1*). Genes found in bipolar cells (*unc5d*), and amacrine cells (*prdm13*) were also present in Clusters 21 and 22, respectively (Figure S9d,e, see the NB vs. BB tab in Spreadsheet S2), suggesting that neurobots could potentially sense and process light stimuli.

Many other clusters included significant enrichment in genes encoding various aspects of the nervous system. This included Cluster 5, which contained various synapsins, tubulins, and microtubule‐associated proteins, which are implicated in biological processes such as the synaptic vesicle cycle, neuron development, regulation of neurotransmitter secretion, and synaptic vesicle localization (Figure S9f, see the NB vs. BB tab in Spreadsheet S2). Cluster 9 revealed the presence of voltage‐gated ion channels including various types of sodium and potassium channels as well as voltage‐gated calcium channels (Figure S9g, see the NB vs. BB tab in Spreadsheet S2). Cluster 11 contained genes encoding various G‐protein coupled receptors, as well as those implicated in the modulation of chemical synaptic transmission (Figure S9h, see the NB vs. BB tab in Spreadsheet S2). Cluster 14 revealed the presence of various hormones and neuropeptides and their receptors (Figure S9i, see the NB vs. BB tab in Spreadsheet S2). Cluster 21 included genes important for axonogenesis and neuron projection development (Figure S9d, see the NB vs. BB tab in Spreadsheet S2).

Moreover, we found that there was significant upregulation in genes encoding ECM constituents (Figure 12a, Cluster 20, and Figure S9j, see the NB vs. BB tab in Spreadsheet S2) including collagen (*col17a, col4a2*), which is the most abundant fibrous protein and constitutes the main structural element of ECM [46], and fibulins [64], which are glycoproteins that are secreted in the ECM and provide mechanical support in connective tissue (*fbln1*). Our Second Harmonic Generation imaging indeed showed the presence of some collagen fibers within the central cavity of neurobots, although they were only detected at very low levels (Figure S4). Future experiments are required for assessing the presence of fibulins and other potential constituents of the ECM in this space.

As expected, network embedding and clustering analysis of the upregulated genes in neurobots relative to shams similarly revealed overexpression of genes relating to synapse organization, regulation of neurotransmitter and receptor activity, chemical synaptic transmission (within Clusters 4,5), visual perception (within Cluster 3), neuron projection, and perineuronal nets (within Cluster 2, Figure S10). These findings shed light on biological pathways/molecular functions that are innately present in neurobots in the absence of any external manipulations and will inform future work toward building specialized neurobots through selective enhancement of these pathways.

**FIGURE 14:**
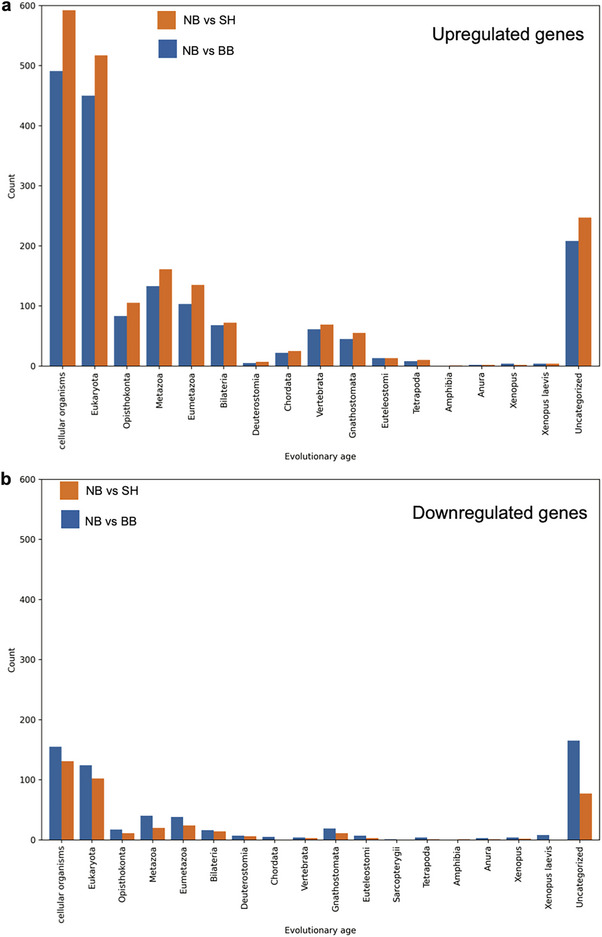
Phylostratigraphic analysis of upregulated or downregulated transcripts in neurobots compared to biobots and shams. (a) 54% of upregulated genes in neurobots fall into the two categories of most ancient genes (“All living organisms” and “Eukaryota”). (b) Very few ancient genes are downregulated. In total 279 are downregulated in these two strata for the NB versus BB conditions and 233 for the NB versus SH condition, whereas 941 and 1109, genes are upregulated respectively.

Finally, we tested the hypothesis that neurobots are expressing a more ancient transcriptome as a result of their nascent evolutionary history. We applied a phylostratigraphic analysis for the differentially expressed genes in the different conditions (Figure 14, NB vs SH and NB vs BB). Interestingly, we found that more than 54% of upregulated genes in neurobots fall into the two categories of most ancient genes (“All living organisms” and “Eukaryota”, Figure 14a). By comparison, very few ancient genes are downregulated. In total 279 genes are downregulated in these two strata for the NB vs BB conditions, and 233 for the NB vs SH condition (Figure 14b), while for the upregulated genes, we obtained 941 and 1109, respectively. Therefore, we conclude that the development of neurobots involves a transcriptomic shift toward very ancient genes for neurobots compared to biobots and shams.

## 3 Discussion

In this study, we built and investigated behavioral, anatomical, and transcriptional properties of novel living constructs with incorporated neural tissue. Using *Xenopus laevis* embryonic cells, we built two types of living constructs: one using ectodermal cells (biobots) as reported in prior studies [25, 48, 65], and another, novel construct, made using ectodermal and neural precursor cells (neurobots, Figure 1a). We showed that neurobots are viable and self‐motile like their non‐neuronal counterparts (Videos S3 and S4), and that the implanted neural precursor cells indeed differentiate into neurons and extend their processes throughout the construct (Figures 1 and 2; Figure S3).

We found that neurobots became significantly larger than biobots and were significantly more elongated (Figure 1b,c). In addition to overall changes in shape and size revealed by our two‐dimensional analysis, neurobots exhibited differences in the 3D distribution of multiciliated cells and in overall body morphology. In the future, a more advanced 3D analysis incorporating the spatial positions of MCCs will help quantify further differences between neurobots and biobots and elucidate how these may correlate with their behaviors.

Neurobots exhibited a large degree of variability in the amount innervation and in neural architecture (Figure 9; Figure S3). The variability in the size and number of the implanted neural precursor cell clumps (due to the manual nature of their creation), could be a major contributing factor to the variability in neural growth and patterning. Indeed, we found a small positive correlation between the relative size of the implanted tissue and the resulting neurite length and number of neural terminals (see Figure 9c). Another source of variability may stem from the need to pool neural precursors from multiple embryos to generate sufficiently large neuronal clumps for manual implantation. Consequently, the individual cells within each clump were unlikely to be genetically identical and may have differed slightly in their developmental stage. Developing automated methods to implant a defined number of cells, ideally derived from the same embryo, will facilitate more accurate quantification of the relationship between implant size and subsequent growth, and will likely reduce variability across neurobots. Efforts are underway to develop robot scientist platforms for the field of synthetic morpho‐engineering.

We found that the majority of neural processes emanating from the implanted neural precursor clumps grew within the central cavity of the bot. However, some processes clearly extended toward the outer epithelium (arrowheads Figure 2; Figure S3). Future experiments will investigate more thoroughly how neurons modulate the activity of outer epithelial cell types, including multiciliated cells, goblet cells, and serotonergic cells. For example, optogenetic activation of neurons combined with imaging of fluorescent microbead flow patterns could be used to test the causal relationship between neural activity and ciliary beating frequency. When paired with genetic knockdown of relevant cell‐surface receptors, such as serotonergic receptors [29], such approaches could help elucidate the mechanisms by which neurons influence movement in neurobots. It is important to note that, unlike in some invertebrates where ciliary beating frequency, and thus cilia‐driven movement, is under neural control [32], the beating frequency of MCCs on tadpole skin has not been shown to be under neural control. Instead, it is thought to be controlled by molecules secreted by other epithelial cells for example, serotonin (through small secretory cells) [29], and may also be controlled by factors known to influence beating frequency of motile cilia in other mucociliary epithelia such as extracellular ATP molecules secreted in the context of injury or inflammation [66, 67, 68], and noxious sensory stimuli [69]. The emergence of neural regulation of ciliary beating frequency in neurobots would therefore represent a novel property.

Despite variation in the shape and innervation patterns of neurobots, we found that all of them exhibited a central cavity largely devoid of cell bodies (as indicated by the absence of nuclear staining) and containing only a few neurites with sparse expression of the presynaptic marker synapsin‐1 (Figures 2 and 3; Figure S3). We confirmed the largely acellular nature of this space through staining with phalloidin, which labels F‐actin present in all eukaryote cells (Figure S4). We speculated that this region may be filled with extracellular matrix‐like structures. The presence of neurites extending in extremely straight courses points to the presence of such a supporting structure (Figure 2b, red arrowhead). Further, our transcriptomic analysis demonstrated a significant upregulation of genes encoding ECM proteins, including both fibrillar collagens and non‐fibrillar components such as fibulins. In order to assess whether this space contains fibrillar ECM proteins, we used Second Harmonic Generation, which is a label‐free method for visualizing fibrillar ECM proteins such as collagen [45]. Although we found evidence for the presence of collagen fibers, these fibers were extremely sparse (Figure S4). Other non‐fibrillar ECM components, such as proteoglycans, laminins, or fibronectins, may occupy much of this space. It will be important for future work to characterize the molecular composition of this region in greater detail.

Neurobots exhibited a diverse range of movement patterns, and these patterns tended to be more complex than those observed in their non‐neuronal counterparts, indicating that neural expression may affect movement either directly, that is, through neural signaling to the motor effectors, or via changes in the expression patterns of motor effectors. Indeed, we observed a negative correlation between the degree of neural expression and the density of multiciliated cells (Figure 9c). Future modeling efforts could help determine the extent to which differences in the spatial distribution of MCCs alone contribute to the observed increase in movement complexity.

Simultaneous recording of neural activity and behavior could be used to assess the potential neural correlates of the behavior. Indeed, our calcium imaging experiments indicated that the implanted neurons were spontaneously active; however, due to technical difficulties in measuring calcium signals in freely moving neurobots, we were not able to make conclusions about neural correlates of observed movements. Specifically, measuring calcium in freely moving neurobots was complicated by their rotational movements, which often resulted in losing track of specific regions of interest (Video S3). Such 3D rotational movements could be suppressed by making flattened, disk‐like neurobots, however, these neurobots tended not to move as much (data not shown). Future experiments in which the 3D movement of the bots can be better controlled without flattening, as well as experimental setups that allow repeated measurements, will be critical for establishing correlations between neural activity and behavior.

Albino, pigment‐less *Xenopus laevis* embryos were used to generate the body of neurobots intended for calcium imaging, as the lack of pigmentation facilitates visualization of neuronal calcium activity in the interior. Albino embryos exhibit developmental trajectories similar to those of wild‐type embryos [70], but differences in visually evoked behaviors have been reported in albino tadpoles [71, 72]. Thus, possible differences in the pattern of neural growth and behavioral phenotypes of neurobots with and without body pigmentation, especially in the context of visually‐evoked behaviors, may be an interesting subject of future investigation.

Consistent with a role of neural activity in modulating behavior, we found that treatment of biobots and neurobots with the GABA_A_ receptor antagonist PTZ resulted in significantly different outcomes. To our surprise, we found that most biobots decreased their movement complexity with PTZ treatment, indicating the presence of non‐neuronal drug targets. Indeed GABAergic receptors are found on the surface of the mucus‐secreting goblet cells, and treating *Xenopus* embryos with bicuculline, which is also a GABA_A_ antagonist, was shown to inhibits mucus secretion [73]. It is possible that treatment with PTZ changes the beating frequency of MCCs through changes in mucus secretion. The effect of PTZ on neurobots was significantly different from that on biobots (Figure 8). In fact, the majority of neurobots showed an increase in movement complexity, suggesting that neural activity may contribute to the observed differential effect. Additional experiments are required to identify the targeted cell types (neuronal or non‐neuronal) and to assess whether the effect we observed is caused by GABA signaling or is due to off‐target effects of PTZ. Although PTZ is a noncompetitive antagonist of GABA_A_ receptors, it may also exert indirect effects on other targets at higher concentrations, including glutamate receptors and voltage‐gated ion channels [74]. Future experiments where the activity of all or a specific population of neurons could be modulated pharmacologically or optogenetically could provide important insight on the causal relationship between neural activity and behavior, as well as the underlying mechanisms.

We raised a group of neurobots in zolmitriptan, which is a selective 5‐hydroxytryptamine (5‐HT) 1B/1D receptor agonist, known to increase the degree of ectopic neural growth in *Xenopus* embryos [51]. Interestingly, three out of four of these neurobots showed an increase in movement complexity when treated with PTZ (filled circles Figure 8b; Figure S7). Moreover, these neurobots showed a tighter correlation between the amount of implanted tissue and the degree of innervation (Figure S7). Interestingly, 5‐HT_1B_ receptors are shown to modulate GABA release [75], and serotonergic signaling is thought to affect the migration pattern of cortical interneurons [76]. Altered serotonergic signaling in neurobots treated with zolmitriptan may therefore impact the expression of GABAergic neurons during their development. Further experiments are needed to discover the mechanisms underlying this effect and whether zolmitriptan treatment results in biased expression of specific neural subtypes.

Our transcriptomics analysis revealed the landscape of differentially expressed genes between neurobots, biobots, and sham neurobots. Overall, neurobots showed a significant upregulation in gene expression compared to biobots and sham neurobots, whereas biobots and shams were more similar to one another (Figure 10a,b and Figure 11). Functional enrichment analysis revealed that neurobots, compared to both biobots and shams, exhibit a high level of enrichment in genes involved in nervous system development, synapse formation, neuron projection, and trans‐synaptic signaling (Figure 12a,b). Genes encoding major neurotransmitter receptors were present in the transcriptome of neurobots. This included glutamatergic, GABAergic, cholinergic, dopaminergic, serotonergic, and glycinergic receptors.

Our gene network analysis resulted in the identification of multiple functional clusters, allowing us to more deeply examine the genes and pathways that are upregulated in neurobots. Notably, we found a large cluster containing genes with important roles in visual perception (Cluster 1, Figure 13c; Figure S9c). This cluster contained genes normally expressed exclusively in *Xenopus* eyes, including various members of the opsin family such as a retinal G protein‐coupled receptor, various cone opsins, rhodopsin, as well as genes encoding many other proteins implicated in visual processing. This remarkable finding suggests the possible presence of visually evoked behaviors in neurobots. The next and most exciting step will be to test this hypothesis and discover the ways that light could modulate motor output in neurobots. If present, this will be a completely novel emergent behavior. Follow‐up proteomic analyses will be necessary to determine whether the upregulated transcripts are translated into corresponding proteins and to investigate their spatial organization.

We showed that neurobots exhibit a significant increase in the variability of their gene counts compared to their non‐neuronal counterparts (BBs and SHs, Figure 10c–f). Although some of this variability could be attributable to the manual nature of their creation, and/or pooling of implanted cells from multiple embryos, the excess variability seen in neurobots relative to shams suggests that neurons may play an important role in guiding the way cells explore the gene expression landscape. The nervous systems of animals are known to influence the behavior of non‐neural cells and tissues [60, 77], so it is possible that the information processing activity of the neurons, in response to the unique “life experiences” of individuals, or internally‐generated spontaneous signaling, might account for the neurobots exhibiting the largest inter‐individual gene expression variability of the groups. Moreover, studies show that when cells are exposed to novel stressors that they do not have existing homeostatic mechanisms to resolve, they resort to making random changes in the expression levels of many genes. It is possible that the bots we report here are undergoing stressors that evolution did not prepare them for, and may be employing this kind of exploration of gene expression space [78, 79, 80, 81, 82]. Future single‐cell RNA‐seq experiments, combined with methods for implanting defined numbers of cells, ideally derived from a single embryo, will provide critical insight into cell‐to‐cell variability and determine whether increased transcriptional variability in neurobots is confined to specific cell types.

Finally, based on a phylostratigraphic analysis, we show that the majority of upregulated genes in neurobots consist of the most ancient genes (Figure 14), a pattern that differs significantly from that observed in biobots and shams. In all cases, the cells were wild‐type, and no genomic editing, synthetic biology circuits, scaffolds, or drugs were used. These results suggest that novel configurations of cell types can have large‐scale systemic effects on the transcriptome of the resulting multicellular construct and move it toward the gene expression profiles of the evolutionary past.

Building neurobots with predictable nervous system architecture and in large numbers is one of the major remaining challenges of this study. Future automation and standardization efforts will enable higher throughput and consistency, allowing repeated measurements to be performed on neurobots with both identical and distinct nervous system architectures. The impact of pharmacological, optical, and other types of stimulation on the neurobot behavior could be assessed. Additionally, the creation and neuroanatomical characterization of large numbers of identical bots will allow for the discovery of frequently emerging patterns and motifs, thereby shedding light on the potential space for nervous system architectures in novel living constructs whose precise layout has not been shaped by selection for this specific behavioral configuration.

This study establishes a model system and experimental roadmap to increase our understanding of the plasticity of evolutionarily determined hardware of living beings to adapt on developmental (not evolutionary) timescales and to provide interfaces to bioengineered living constructs that may provide novel control capabilities for useful synthetic living machines and shed light on the origins of novelty in the evolution of nervous systems.

## 4 Methods

### 4.1 Animal Husbandry and Construction of Biobots, Neurobots, and Shams

All experiments were approved by Tufts University Institutional Animal Care and Use Committee (IACUC) under the protocol number M2023‐18.

Biobots were constructed as described previously [25], by excising tissue from the animal hemisphere of a Nieuwkoop and Faber stage 9 *Xenopus laevis* embryo (animal cap).

To construct neurobots and sham neurobots we excised 40–50 such animal caps and let them sit with external surface facing up in 60 mm petri dishes filled with a calcium and magnesium free solution (50.3 mM NaCl, 0.7 mM KCl, 9.2 mm Na_2_HPO_4_, 0.9 mm KH_2_PO_4_, 2.4 mm NaHCO_3_, 1 mm edetic acid (EDTA), pH 7.3), and coated with 1% agarose made in the same solution. After about 30–40 min the cells were fully dissociated. The dissociated cells were transferred to a deep 60 mm petri dish containing 0.75 Marc's Modified Ringer (MMR) solution, using a P200 pipette, taking as little liquid as possible. For constructing sham neurobots, the dissociated cells were immediately reaggregated and formed into clumps (see below). For constructing neurobots, we dispersed the dissociated cells as far as possible by moving the solution in the dish using a P1000 pipette. The cells were left still in the dish for ∼3–4 h. To reaggregate cells, the dish containing dissociated cells was placed on a shaker, and cells were thereby brought together in the middle of the dish. They were then allowed to reaggregate for approximately 1 h, at which point various clumps of cells formed spontaneously.

Using a P1000 pipette, clumps of ∼>10 cells were moved into the wells of an agarose‐coated 6‐well plate. Larger clumps were broken into smaller ones that could fit inside the body of the bot, that is animal caps excised from a new set of embryos (see Figure 1a). Depending on the size of the clumps, one or more were implanted.

Next, a new set of animal caps was dissociated from a second batch of embryos from a later fertilization (at late blastula, early gastrula stage), and were placed with the external surface facing down individually in the wells of the same 6‐well plate. The excised animal caps slowly formed a bowl and eventually closed up within approximately 10–15 min. Clumps of neural precursor cells (or non‐neuronal clumps in the case of shams) were placed inside this bowl (Figure 1a (ii)) before it was closed using fine forceps, and enough time was allowed for the animal cap to fully close before moving the dish to the incubator. Bots were housed in an incubator set to 14°C, and experiments were performed in a laboratory maintained at approximately 18°C.

### 4.2 Immunohistochemistry

Bots were fixed overnight at 4°C in 4% paraformaldehyde (Thermo Fisher Scientific) with 0.25% gluteraldehyde (Electron Microscopy Sciences) individually in 96‐well plates. The next day, they were washed three times at room temperature in PBS‐Triton X(PBT, 0.1% Triton X‐100 (Sigma) in PBS‐/‐ (Gibco)) for at least 15 min and then incubated in the 10% Casblock (invitrogen) dissolved in PBT for at least 1 h. They were then transferred into the solution containing primary antibodies and Hoescht (33342, Thermo Scientific). The plate was sealed using parafilm and covered in foil for light protection and placed on a shaker in the cold room for 3 days at 4°C. The bots were next washed 3 times in PBT at room temperature and then transferred and incubated overnight at 4°C in the secondary antibodies, with the dish sealed with parafilm and covered in foil. Finally, the bots were washed again 3 times in PBT and either stored in PBS at 4°C or mounted into 15‐slide 18‐well flat dishes (81821, Ibidi) in an antifade mounting medium (Vectashield, Vectorlabs H‐1000‐10) for confocal imaging. Primary antibodies used were (Anti‐acetylated tubulin antibody, Mouse monoclonal, Sigma T7451; Anti‐synapsin‐1 antibody, Guinea pig monoclonal recombinant antibody, Sysy Antibodies 106 308; MAP2 antibody, Rabbit polyclonal antibody, Cell Signaling technologies 4542), and the corresponding secondary antibodies were (Goat anti‐mouse Alexa 594, Invitrogen A32742, Goat anti‐guinea pig IgG (H+L) highly cross‐adsorbed secondary antibody, Alexa Fluor 488, ThermoFisher Scientific A‐11073, Goat anti‐rabbit IgG (H+L) cross‐adsorbed secondary antibody, Alexa Fluor 488, ThermoFisher Scientific A‐11008). For F‐actin staining fixed and permeabilized neurobots were incubated with the F‐actin‐specific stain, Alexa Fluor 647 Phalloidin (Thermo Fisher Cat # A22287) at 1:50 in PBS (‐/‐) overnight at 4^○^C. Neurobots were washed 3× with PBS (‐/‐) at room temperature and subsequently mounted in Vectashield.

### 4.3 Confocal and Multiphoton Microscopy

Confocal images were acquired using the Stellaris 8 microscope (Leica Microsystems), equipped with a Chameleon Vision II (Coherent; 80 MHz repetition rate). Z‐stacks were collected at 2 µm intervals with a 25 ×/0.95 NA or a 40×/ 1.1 NA water‐immersion objectives. Each signal was acquired in series. To ensure deep tissue penetration, Alexa 647‐Phalloidin was excited by two‐photon excitation at 819 nm, with emission collected between 636 and 746 nm. Second harmonic generation (SHG) imaging was performed at 890 nm excitation,  with emission detected between 465 and 616 nm, as previously described [45]. Fiji (Image J), the LAS X (Leica Microsystems), and Imaris (Oxford Instruments) programs were used to process the images.

### 4.4 Calcium Imaging

Embryos at the four‐cell stage were microinjected in all four blastomeres with mRNA encoding the genetically encoded fluorescent calcium indicator GCaMP6s. These embryos were used for obtaining clumps of neural precursor cells for implantation. Albino embryos were used as the outer shell of the neurobots in these experiments so that the fluorescence signal from neurons could be visualized more easily as wild‐type embryos are pigmented. We used a custom‐built microscope to measure calcium activity in freely moving neurobots (Video S1). We used Fiji's [83] Descriptor Based Series Registration plugin to correct for the motion of the neurobot (Video S2), then used Suite2p software [84] to identify active units (Figure 5). To avoid movements in the *Z*‐direction, which resulted in changes in the plane of focus, we created flattened neurobots as described previously [48]. Briefly, on the day after their formation, neurobots were pressed down using a glass coverslip, which was gradually lowered over them as small amounts of MMR were removed from the dish. Neurobots were left under pressure for 3 h, after which MMR was gradually added to the dish resulting in the release of the coverslip.

### 4.5 Quantification of the Bot Shape and Neural Tracing

We used the brush tool in Fiji [83] to fill in the shape of the bot and calculated the area and roundness index defined as the major_axis / minor_axis. We used the same tool to estimate the relative amount of implanted neural precursor tissue by dividing the area of the implanted clumps to the outer shell (Figure 1a). We used Imaris software (Oxford Instruments) to quantify neural expression using confocal stacks acquired from the bots that were stained with antibodies against acetylated α‐tubulin, which labeled neurons and cilia in multiciliated cells. We manually traced neurites using the filament function and exported values corresponding to the total length of neurites (dendrite length sum parameter in Imaris) and the number of terminal points (number of dendrite terminal points parameter in Imaris). For the analysis of the multiciliated cell distribution, we estimated the total number of MCCs by marking the center of each MCC using the Spots tool in Imaris and calculated the total number of multiciliated cells. We then used this value to calculate the MCC density by dividing this number by the total area of the bot.

### 4.6 Behavioral Analysis

Videos of bot movements were taken over 30 min under various conditions and tracked with the DLTdv digitizing tool [49] in MATLAB, and the *x* and *y* coordinates of the center of mass were calculated. A custom Python code was used to extract various kinematic variables using the time series of the coordinates. We calculate total Euclidean distance travelled, average speed, and average acceleration. Additionally, we calculated the percentage of the well that was traversed by the bots by dividing the space of each well into 0.1 mm bins. We then calculated the percent covered area by dividing the number of unique visited bins by the total number of bins. We calculated a complexity index by first calculating the power spectral density (PSD) of the trajectory time series along the *x* and *y* coordinates and identifying peaks in the power. We calculated Welch's power spectral density estimate with a window size of 400 s and an overlap of 1 s between windows for each of the *x* and *y* time series. We picked a threshold of 10 pixels^2^/Hz (∼0.17 mm^2^/Hz = 0.4 mm/Hz) to detect peaks in the PSD of the *x* and *y* coordinates. This threshold was chosen empirically to remove the baseline noise corresponding to the tracking of the center of mass of bots that had an average radius of 0.4 mm. We then defined the complexity index as the total number of unique peaks in the *x* and *y* PSDs.

To further confirm the significance of power at the peak frequencies identified by Welch power analysis, we used the wavelet transform and surrogate data. For each time series, we used MATLAB to calculate the continuous wavelet transform and from there calculated the wavelet power spectrum across all time. To assess the significance of dominant frequencies found from the Welch spectral analysis, we generated surrogate time series that preserve the original signal's amplitude spectrum but randomize its phase information in the wavelet space. These surrogates were used to compute a distribution of wavelet power spectra, from which significance thresholds (95th percentile) were derived for hypothesis testing. Only 4 out of 243 peaks identified through Welch analysis across all bots did not pass the significance test in the wavelet space. This small difference is expected because the two methods differ in their frequency resolution: Welch analysis uses uniform resolution across all frequencies, whereas wavelet analysis employs logarithmic spacing, providing finer detail at lower frequencies and coarser resolution at higher frequencies.

### 4.7 RNA‐Sequencing and Bioinformatics

We submitted 12 samples (4 samples per biobot type, 5–15 bots per sample: Number of bots per sample: {NB1 = 14, NB2 = 12, NB3 = 12, NB4 = 12}, {BB1 = 16, BB2 = 6, BB3 = 6, BB4 = 5}, {NN1 = 5, NN2 = 6, NN3 = 6, NN4 = 5}) submerged in Trizol (Invitrogen) in 2 mL Eppendorf tubes, to Novogene (Novogene Corporation Inc., Sacramento, CA) for low‐input, high‐lipid, bulk RNA extraction. The first batch submitted to Novogene (NB1 and BB1) contained a larger number of bots to test the efficacy of the low‐input method. The next series of sample batches (NB2‐4, BB2‐4, NN1‐4) contained a smaller number of bots based on the results from the first batch. Equal quantities of RNA were sequenced from each sample using the NovaSeq6000 sequencer, resulting in consistent library size across samples. Clean reads were extracted from FASTQ files, removing reads with adapter contamination, when uncertain nucleotides constitute more than 10 percent of either read (*N* > 10%), and when low‐quality nucleotides (Base Quality less than 5) constitute more than 50 percent of the read. The index to the reference genome (*Xenopus laevis* version 10.1) was built using Hisat2 v2.0.5 [85], and clean reads were aligned to the reference. The mapped reads of each sample were assembled using StringTie (v1.3.3b) [86], and FeatureCounts v1.5.0‐p3 [87] was used to count the read numbers mapped to each gene. FPKM of each gene was calculated based on the length of the gene and read counts mapped to this gene. Differential expression analysis was performed using the DESeq2 R package (1.20.0) [88], and the resulting *p*‐values were adjusted using Benjamini and Hochberg's approach for controlling the false discovery rate.

The webapp g: Profiler [62] was used to perform functional enrichment analysis of differentially expressed genes. For each comparison, upregulated genes (*p*‐adjusted < 0.05; log2foldchange > 4) and downregulated genes (*p*‐adjusted < 0.05; log2foldchange < ‐4) were separately mapped from *Xenopus* to human symbols using the HGNC Comparison of Orthology Predictions (HCOP) tool [89]. Genes lacking an established gene symbol were removed from analysis. Each gene list was separately queried using g: Profiler across all data sources. The statistical data scope included only annotated genes, and the g: SCS method was used for computing multiple testing corrections for *p‐*values at a threshold of *p *< 0.05. The R package ggplot2 (v3.5.1) [90] was used to generate dot plots of gene ontology driver terms from g: Profiler. Driver terms were determined by grouping significant terms into sub‐ontologies based on their relations, then identifying the leading gene sets that give rise to other significant functions in the ontology neighborhood.

For network analysis and clustering, we applied network analysis techniques to discover biological functional modules [91, 92]. By integrating gene expression and interaction data, we extracted PPI for the different biobots in their respective conditions and applied network embedding and clustering techniques similar to those described by Cantini et al. [93] and Pio‐Lopez et al. [94]. Specifically, we used the MNMF algorithm developed by Wang et al. [61] for network embedding and clustering. To create a network for the bots, we started by isolating genes of interest and identifying corresponding human orthologs under various conditions using the HCOP database [95]. We then used the STRING database [96] to extract relevant PPI networks. The clusters identified through our network embedding and clustering method were further analyzed for enrichment using g: Profiler [62].

### 4.8 Analysis of Gene Expression Variability

The normalized gene count variability was compared between groups (BBs, NBs, and SHs) using a MATLAB script and the method summarized in Figure S11. We excluded NB1 and BB1 since they were sent to Novogene for sequencing as a different batch than other NBs (NB2‐4) and BBs (BB2‐4), and we wanted to reduce the impact of inter‐batch variability (see *RNA‐sequencing and bioinformatics)*. We kept all four NN groups as they were sent as a part of the same batch (NN1‐4). For each gene in each pair of groups being compared, the mean count value across all pools of both groups was found, and genes were ranked from greatest to least mean. Genes for which any of the counts across all pools was 0 were discarded. Because the count value of a given gene in a given pool represents the mean value of all the individuals in that pool, the standard deviation of the pools gives the standard error of the means (SE) of the group. The SE is related to the number of individuals per pool (*n*) and the standard deviation of the individuals within the pools (*σ*) with the equation SE = *σ*/sqrt(*n*). By multiplying the SE by sqrt(*n*), *σ* can be calculated. Dividing σ by the mean count value of the pools gives the coefficient of variation (CV). The ranked gene CV lists were then split into 100 bins (percentiles) containing equal numbers of genes from highest to lowest counts. Within each bin, the number of genes with greater CV for the first group than the second was counted and divided by bin size to find the fraction of genes in the bin with greater CV in the first group. These fractions were then plotted as bar graphs in Figure 10c–e, with a blue line marking 0.5. The bin values appeared to vary with gene count percentile, so to determine the statistical significance of each bin's departure from its expected value, a permutation test was used. For each plot (each pair of groups), the order of the gene pairs was randomly shuffled (keeping pairs together), and new bins were generated. This was repeated 1000 times for random shuffles to produce a distribution of bin fraction values for each bin. The *p*‐value of each bin was defined as the proportion of the bin fractions from the distribution that were further in absolute value from the distribution mean than the true bin fraction. Bins with *p*‐values of *p* < 0.05 were deemed statistically significant and were colored dark blue. in Figure 10d‐f. All other bins were colored light blue. The mean bin value from the distributions was plotted as a yellow line.

### 4.9 Phylostratigraphic Analysis

We employed the phylostrat package [97] to conduct a phylostratigraphic analysis on transcripts of all three bot types, with *Xenopus* laevis (taxon ID 8355) designated as the reference species. This software automated several key steps in evolutionary analysis: (1) it built a clade tree using species from the UniProt database and aligned it with the latest NCBI taxonomy; (2) the clade tree was trimmed to maintain a phylogenetically diverse selection of representative species for each phylostratum; (3) a comprehensive protein sequence database was constructed from hundreds of species based on this clade tree, with additional data such as human and yeast proteomes manually added, resulting in 329 species for our study; (4) a similarity search was performed by conducting pairwise BLAST comparisons between the proteins encoded by *Xenopus laevis* and those of the target species; (5) the best hits were identified, and gene homology was inferred between *Xenopus laevis* and the target species; (6) each gene was assigned to a phylostratum that corresponded to the oldest clade for which a homolog was identified. Genes specific to *Xenopus laevis* were classified as orphan genes and placed within the *Xenopus laevis* phylostratum. The evolutionary stages we focused on include: All living organisms (bacteria, eubacteria), Eukaryota, Opisthokonta, Metazoa, Eumetazoa, Bilateria, Deuterostomia, Chordata, Vertebrata, Gnathostomata, Euteleostomi, Sarcopterygii, Tetrapoda, Anura, *Xenopus*, and *Xenopus laevis*. This methodological approach enabled a detailed examination of gene emergence and their evolutionary trajectories across various taxa. By implementing Phylostratr, we systematically mapped the age of the bot genes in the different conditions with a specific phylostrata to understand the distribution of ages of the bots’ overexpressed genes. We used the upregulated and downregulated genes in neurobots (log2foldchange > 4 and log2foldchange < ‐2 respectively).

### 4.10 Statistical Analysis

Statistical analyses of behavioral and shape data were performed using the non‐parametric Kruskal–Wallis test. When appropriate, multiple comparisons conducted using Tukey's honestly significant difference test. For PTZ experiments, we used a one‐sample *t*‐test to assess whether the population mean of the relative complexity index was significantly different from zero. Robust linear regression was used to quantify relationships between pairs of neurobots’ physical, neuroanatomical, and behavioral measures; the significance of regression coefficients and Pearson correlation coefficients was assessed using two‐tailed *t*‐statistics. Significance of peak frequencies identified by Welch analysis was validated using continuous wavelet transforms and phase‐randomized surrogate time series to generate null distributions of wavelet power, with significance assessed at the 95th percentile. In box plots, whiskers showed the non‐outlier extent, + signs depicted outliers, and the top and bottom of the box showed the upper and lower quartiles of the data. The horizontal bar inside the box showed the median. MATLAB (Mathworks, Natick, MA) functions were used for all statistical analyses.

The statistical analysis for differential gene expression data was performed using the R package DESeq2, which employed the two‐sided Wald test. Multiple hypothesis testing corrections were used to obtain adjusted *p*‐values. For the analysis of gene expression variability, the statistical significance of bin deviations from expected values was assessed using a permutation test. For each group comparison, gene pairs were randomly shuffled 1000 times (with pairs kept intact) to generate null distributions of bin fractions; bin *p*‐values were defined as the proportion of permuted values more extreme than the observed value, and bins with *p *< 0.05 were considered significant.

## Funding

This research was supported by HR0011‐18‐2‐0022, W911NF1920027, awarded by the Department of Defense, and grants from the John Templeton Foundation and Northpond Ventures.

## Conflicts of Interest

M.L. is a scientific co‐founder and consults for Fauna Systems, a company seeking to commercialize frog cell‐based biobot technology.

## Supporting information




**Supporting File 1**: advs74389‐sup‐0001‐SuppMat.pdf.

**Supporting File 2**: advs74389‐sup‐0002‐SuppMat.docx.

**Supporting File 3**: advs74389‐sup‐0003‐VideoS1.mp4.

**Supporting File 4**: advs74389‐sup‐0004‐VideoS2.mp4.

**Supporting File 5**: advs74389‐sup‐0005‐VideoS3.mp4.

**Supporting File 6**: advs74389‐sup‐0006‐VideoS4.mp4.

**Supporting File 7**: advs74389‐sup‐0007‐SuppMat.docx.

**Supporting File 8**: advs74389‐sup‐0008‐Data.zip.

## Data Availability

RNA‐sequencing data generated during this study are available in the NCBI Gene Expression Omnibus (GEO) public repository under accession number GSE295614. All other data supporting the findings of this study are available from the corresponding authors upon reasonable request.

## References

[1] X. Navarro , M. Vivó , and A. Valero‐Cabré , “Neural Plasticity After Peripheral Nerve Injury and Regeneration,” Progress in Neurobiology 82 (2007): 163–201, 10.1016/j.pneurobio.2007.06.005. 17643733

[2] A. Antonini and M. P. Stryker , “Rapid Remodeling of Axonal Arbors in the Visual Cortex,” Science 260 (1993): 1819–1821, 10.1126/science.8511592. 8511592

[3] H. T. Cline , M. Lau , and M. Hiramoto , “Activity‐Dependent Organization of Topographic Neural Circuits,” Neuroscience 508 (2023): 3–18, 10.1016/j.neuroscience.2022.11.032. 36470479 PMC9839526

[4] T. R. Makin and H. Flor , “Brain (re)Organisation Following Amputation: Implications for Phantom Limb Pain,” Neuroimage 218 (2020): 116943, 10.1016/j.neuroimage.2020.116943. 32428706 PMC7422832

[5] D. J. Blackiston and M. Levin , “Ectopic Eyes Outside the Head in Xenopus Tadpoles Provide Sensory Data for Light‐Mediated Learning,” Journal of Experimental Biology 216 (2013): 1031–1040, 10.1242/jeb.074963. 23447666 PMC3587383

[6] R. George , M. Chiappalone , M. Giugliano , et al., “Plasticity and Adaptation in Neuromorphic Biohybrid Systems,” iScience 23 (2020): 101589. 33083749 10.1016/j.isci.2020.101589 PMC7554028

[7] J. Soriano , “Neuronal Cultures: Exploring Biophysics, Complex Systems, and Medicine in a Dish,” Biophysica 3 (2023): 181–202, 10.3390/biophysica3010012.

[8] S. P. Pașca , “The Rise of Three‐Dimensional Human Brain Cultures,” Nature 553 (2018): 437–445. 29364288 10.1038/nature25032

[9] M. A. Lancaster , M. Renner , C. Martin , et al., “Cerebral Organoids Model human Brain Development and Microcephaly,” Nature 501 (2013): 373–379, 10.1038/nature12517. 23995685 PMC3817409

[10] C. A. Trujillo , R. Gao , P. D. Negraes , et al., “Complex Oscillatory Waves Emerging From Cortical Organoids Model Early human Brain Network Development,” Cell Stem Cell 25 (2019): 558–569.e7, 10.1016/j.stem.2019.08.002. 31474560 PMC6778040

[11] G. Quadrato , J. Brown , and P. Arlotta , “The Promises and Challenges of Human Brain Organoids as Models of Neuropsychiatric Disease,” Nature Medicine 22 (2016): 1220–1228, 10.1038/nm.4214. 27783065

[12] A. El Din , L. Moenkemoeller , A. Loeffler , et al., “Human Neural Organoid Microphysiological Systems Show the Building Blocks Necessary for Basic Learning and Memory,” Communications Biology 8 (2025): 1237, 10.1038/s42003-025-08632-5. 40819006 PMC12357958

[13] D. J. Bakkum , Z. C. Chao , and S. M. Potter , “Spatio‐Temporal Electrical Stimuli Shape Behavior of an Embodied Cortical Network in a Goal‐Directed Learning Task,” Journal of Neural Engineering 5 (2008): 310–323, 10.1088/1741-2560/5/3/004. 18714127 PMC2559979

[14] A. Novellino , P. D'Angelo , L. Cozzi , M. Chiappalone , V. Sanguineti , and S. Martinoia , “Connecting Neurons to a Mobile Robot: An In Vitro Bidirectional Neural Interface,” Computational Intelligence and Neuroscience 2007 (2007): 12725, 10.1155/2007/12725. 18350128 PMC2266971

[15] B. J. Kagan , A. C. Kitchen , N. T. Tran , et al., “In Vitro Neurons Learn and Exhibit Sentience When Embodied in a Simulated Game‐World,” Neuron 110 (2022): 3952–3969.e8, 10.1016/j.neuron.2022.09.001. 36228614 PMC9747182

[16] N. Rouleau , N. J. Murugan , and D. L. Kaplan , “Toward Studying Cognition in a Dish,” Trends in Cognitive Sciences 25 (2021): 294–304, 10.1016/j.tics.2021.01.005. 33546973 PMC7946736

[17] O. Aydin , “Neuromuscular Actuation of Biohybrid Motile Bots,” Proceedings of the National Academy of Sciences of the United States of America (2019), 19841–19847. 31527266 10.1073/pnas.1907051116 PMC6778261

[18] O. Aydin , A. P. Passaro , M. Elhebeary , et al., “Development of 3D Neuromuscular Bioactuators,” APL Bioengineering 4 (2020): 016107, 10.1063/1.5134477. 32161837 PMC7064368

[19] S. Park , M. Gazzola , K. S. Park , et al., “Phototactic Guidance of a Tissue‐Engineered Soft‐Robotic Ray,” Science 353 (2016): 158–162, 10.1126/science.aaf4292. 27387948 PMC5526330

[20] J. Wang , X. Zhang , J. Park , et al., “Computationally Assisted Design and Selection of Maneuverable Biological Walking Machines,” Advanced Intelligent Systems 3 (2021): 2000237, 10.1002/aisy.202000237.

[21] N. Ando and R. Kanzaki , “Insect‐Machine Hybrid Robot,” Current Opinion in Insect Science 42 (2020): 61–69, 10.1016/j.cois.2020.09.006. 32992040

[22] S. Tsuda , S. Artmann , and K. Zauner , Artificial Life Models in Hardware ed. M.‐E. Faust and S. Carrier (Springer, 2009), 213–232, 10.1007/978-1-84882-530-7.

[23] W. P. Clawson and M. Levin , “Endless Forms Most Beautiful 2.0: Teleonomy and the Bioengineering of Chimaeric and Synthetic Organisms,” Biological Journal of the Linnean Society 139 (2023): 457–486, 10.1093/biolinnean/blac073.

[24] S. M. Potter , D. A. Wagenaar , R. Madhavan , and T. B. DeMarse , “Long‐Term Bidirectional Neuron Interfaces for Robotic Control, and In Vitro Learning Studies,” in Proceedings of the 25th Annual International Conference of the IEEE Engineering in Medicine and Biology Society (IEEE Cat. No.03CH37439) (IEEE, 2004), 3690–3693.

[25] D. Blackiston , E. Lederer , S. Kriegman , S. Garnier , J. Bongard , and M. Levin , “A Cellular Platform for the Development of Synthetic Living Machines,” Science Robotics 6 (2021): abf1571, 10.1126/scirobotics.abf1571. 34043553

[26] H. J. Kang and H. Y. Kim , “Mucociliary Epithelial Organoids From Xenopus Embryonic Cells: Generation, Culture and High‐Resolution Live Imaging,” Journal of Visualized Experiments 161 (2020): 61604, 10.3791/61604. 32804169

[27] P. Walentek and I. K. Quigley , “What We Can Learn From a Tadpole About Ciliopathies and Airway Diseases: Using Systems Biology in Xenopus to Study Cilia and Mucociliary Epithelia,” Genesis 55 (2017): 23001, 10.1002/dvg.23001. PMC5276738 28095645

[28] E. Dubaissi and N. Papalopulu , “Embryonic Frog Epidermis: A Model for the Study of Cell‐Cell Interactions in the Development of Mucociliary Disease,” Disease Models & Mechanisms 4 (2011): 179–192, 10.1242/dmm.006494. 21183475 PMC3046089

[29] P. Walentek , S. Bogusch , T. Thumberger , et al., “A Novel Serotonin‐Secreting Cell Type Regulates Ciliary Motility in the Mucociliary Epidermis of Xenopus Tadpoles,” Development 141 (2014): 1526–1533. 24598162 10.1242/dev.102343

[30] F. Keijzer , M. van Duijn , and P. Lyon , “What Nervous Systems Do: Early Evolution, Input–Output, and the Skin Brain Thesis,” Adaptive Behavior 21 (2013): 67–85, 10.1177/1059712312465330.

[31] F. Keijzer , “Moving and Sensing Without Input and Output: Early Nervous Systems and the Origins of the Animal Sensorimotor Organization,” Biology & Philosophy 30 (2015): 311–331, 10.1007/s10539-015-9483-1. 26005236 PMC4438119

[32] C. Verasztó , N. Ueda , and L. A. Bezares‐Calderón , “Ciliomotor Circuitry Underlying Whole‐Body Coordination of Ciliary Activity in the Platynereis Larva,” Elife 6 (2017): e26000, 10.7554/eLife.26000. 28508746 PMC5531833

[33] G. O. Mackie , C. L. Singla , and C. Thiriot‐Quievreux , “Nervous Control of Ciliary Activity in Gastropod Larvae,” The Biological Bulletin 151 (1976): 182–199, 10.2307/1540713. 963121

[34] A. G. Collins , J. H. Lipps , and J. W. Valentine , “Modern Mucociliary Creeping Trails and the Bodyplans of Neoproterozoic Trace‐makers,” Paleobiology 26 (2000): 47–55, 10.1666/0094-8373(2000)026<0047:MMCTAT>2.0.CO;2.

[35] A. Ivantsov , A. Nagovitsyn , and M. Zakrevskaya , “Traces of Locomotion of Ediacaran Macroorganisms,” Geosciences 9 (2019): 395, 10.3390/geosciences9090395.

[36] V. Pai , L. Pio‐Lopez , M. Sperry , P. Erickson , and M. X. T. Levin , “Gene Expression Changes in Wild‐Type Cells Comprising a Form of Biobot,” preprint, bioRxiv, August (2024), 10.31219/osf.io/n2jre.

[37] Y. Satou‐Kobayashi , J. Kim , A. Fukamizu , and M. Asashima , “Temporal Transcriptomic Profiling Reveals Dynamic Changes in Gene Expression of Xenopus Animal Cap Upon Activin Treatment,” Scientific Reports 11 (2021): 14537, 10.1038/s41598-021-93524-x. 34267234 PMC8282838

[38] J. Lee , A. F. Møller , S. Chae , et al., “A Single‐Cell, Time‐Resolved Profiling of Xenopus Mucociliary Epithelium Reveals Nonhierarchical Model of Development,” Science Advances 9 (2023): add5745, 10.1126/sciadv.add5745. PMC10081853 37027470

[39] A. Angerilli , P. Smialowski , and R. A. Rupp , “The Xenopus Animal Cap Transcriptome: Building a Mucociliary Epithelium,” Nucleic Acids Research 46 (2018): 8772–8787, 10.1093/nar/gky771. 30165493 PMC6158741

[40] S. I. Wilson and T. Edlund , “Neural Induction: Toward a Unifying Mechanism,” Nature Neuroscience 4, no. 4 (2001): 1161–1168, 10.1038/nn747. 11687825

[41] H. Grunz and L. Tacke , “Neural Differentiation of Xenopus Laevis Ectoderm Takes Place After Disaggregation and Delayed Reaggregation Without Inducer,” Cell Differentiation and Development 28 (1989): 211–217, 10.1016/0922-3371(89)90006-3. 2620262

[42] L. Dehmelt and S. Halpain , “The MAP2/Tau Family of Microtubule‐Associated Proteins,” Genome Biology 6 (2005): 204, 10.1186/gb-2004-6-1-204. 15642108 PMC549057

[43] D. Perdiz , R. Mackeh , C. Poüs , and A. Baillet , “The Ins and Outs of Tubulin Acetylation: More Than Just a Post‐Translational Modification?,” Cellular Signalling 23 (2011): 763–771, 10.1016/j.cellsig.2010.10.014. 20940043

[44] R. Dominguez and K. C. Holmes , “Actin Structure and Function,” Annual Review of Biophysics 40 (2011): 169–186, 10.1146/annurev-biophys-042910-155359. PMC3130349 21314430

[45] C. R. Esquibel , K. D. Wendt , H. C. Lee , et al., “Second Harmonic Generation Imaging of Collagen in Chronically Implantable Electrodes in Brain Tissue,” Frontiers in Neuroscience 14 (2020): 95, 10.3389/fnins.2020.00095. 32733179 PMC7358524

[46] C. Frantz , K. M. Stewart , and V. M. Weaver , “The Extracellular Matrix at a Glance,” Journal of Cell Science 123 (2010): 4195–4200, 10.1242/jcs.023820. 21123617 PMC2995612

[47] M. Z. Lin and M. J. Schnitzer , “Genetically Encoded Indicators of Neuronal Activity,” Nature Neuroscience 19 (2016): 1142–1153, 10.1038/nn.4359. 27571193 PMC5557009

[48] S. Kriegman , D. Blackiston , M. Levin , and J. Bongard , “Kinematic Self‐Replication in Reconfigurable Organisms,” Proceedings of the National Academy of Sciences of the United States of America 118 (2021): 2112672118, 10.1073/pnas.2112672118. PMC8670470 34845026

[49] T. L. Hedrick , “Software Techniques for Two‐ and Three‐Dimensional Kinematic Measurements of Biological and Biomimetic Systems,” Bioinspiration & Biomimetics 3 (2008): 034001, 10.1088/1748-3182/3/3/034001. 18591738

[50] T. Shimada and K. Yamagata , “Pentylenetetrazole‐Induced Kindling Mouse Model,” Journal of Visualized Experiments 136 (2018): 56573, 10.3791/56573. PMC6101698 29985308

[51] D. J. Blackiston , K. Vien , and M. Levin , “Serotonergic Stimulation Induces Nerve Growth and Promotes Visual Learning via Posterior Eye Grafts in a Vertebrate Model of Induced Sensory Plasticity,” npj Regenerative Medicine 2 (2017): 8, 10.1038/s41536-017-0012-5. 29302344 PMC5665622

[52] S. W. Flavell and M. E. Greenberg , “Signaling Mechanisms Linking Neuronal Activity to Gene Expression and Plasticity of the Nervous System,” Annual Review of Neuroscience 31 (2008): 563–590, 10.1146/annurev.neuro.31.060407.125631. PMC2728073 18558867

[53] C. Herrera‐Rincon , V. P. Pai , K. M. Moran , J. M. Lemire , and M. Levin , “The Brain Is Required for Normal Muscle and Nerve Patterning During Early Xenopus Development,” Nature Communications 8 (2017): 587, 10.1038/s41467-017-00597-2. PMC5610959 28943634

[54] N. L. Bray , H. Pimentel , P. Melsted , and L. Pachter , “Near‐Optimal Probabilistic RNA‐Seq Quantification,” Nature Biotechnology 34 (2016): 525–527, 10.1038/nbt.3519. 27043002

[55] G. Colozza and E. M. De Robertis , “Dact‐4 Is a Xenopus laevis Spemann Organizer Gene Related to the Dapper/Frodo Antagonist of β‐Catenin Family of Proteins,” Gene Expression Patterns 38 (2020): 119153, 10.1016/j.gep.2020.119153. 33186756

[56] A. Pawlowski and G. Weddell , “Induction of Tumours in Denervated Skin,” Nature 213 (1967): 1234–1236, 10.1038/2131234a0.

[57] B. Scharrer , “Insect Tumors Induced by Nerve Severance: Incidence and Mortality,” Cancer Research 13 (1953): 73–76. 13032953

[58] B. Scharrer , “Experimental Tumors After Nerve Section in an Insect,” Experimental Biology and Medicine 60 (1945): 184–189, 10.3181/00379727-60-15132. 21006288

[59] A. Kumar and J. P. Brockes , “Nerve Dependence in Tissue, Organ, and Appendage Regeneration,” Trends in Neurosciences 35 (2012): 691–699, 10.1016/j.tins.2012.08.003. 22989534

[60] C. Herrera‐Rincon and M. Levin , “Booting up the Organism During Development: Pre‐Behavioral Functions of the Vertebrate Brain in Guiding Body Morphogenesis,” Communicative & Integrative Biology 11 (2018): 1433440, 10.1080/19420889.2018.1433440. PMC5824965 29497473

[61] X. Wang , “Community Preserving Network Embedding,” in AAAI'17: Proceedings of the Thirty‐First AAAI Conference on Artificial Intelligence (AAAI Press, 2017), 203–209.

[62] L. Kolberg , U. Raudvere , I. Kuzmin , P. Adler , J. Vilo , and H. Peterson , “g:Profiler—Interoperable Web Service for functional enrichment Analysis and Gene Identifier Mapping (2023 Update),” Nucleic Acids Research 51 (2023): W207–W212, 10.1093/nar/gkad347. 37144459 PMC10320099

[63] J. Hahn , A. Monavarfeshani , M. Qiao , et al., “Evolution of Neuronal Cell Classes and Types in the Vertebrate Retina,” Nature 624 (2023): 415–424, 10.1038/s41586-023-06638-9. 38092908 PMC10719112

[64] W. S. Argraves , H. Tran , W. H. Burgess , and K. Dickerson , “Fibulin Is an Extracellular Matrix and Plasma Glycoprotein With Repeated Domain Structure,” The Journal of Cell Biology 111 (1990): 3155–3164, 10.1083/jcb.111.6.3155. 2269669 PMC2116371

[65] S. Kriegman , D. Blackiston , M. Levin , and J. Bongard , “A Scalable Pipeline for Designing Reconfigurable Organisms,” Proceedings of the National Academy of Sciences of the United States of America 117 (2020): 1853–1859, 10.1073/pnas.1910837117. 31932426 PMC6994979

[66] S. D. Joshi , T. R. Jackson , L. Zhang , C. Stuckenholz , and L. A. Davidson , “Supracellular Contractility in Xenopus Embryo Epithelia Regulated by Extracellular ATP and the Purinergic Receptor P2Y2,” Journal of Cell Science 138 (2025): jcs263877, 10.1242/jcs.263877. 40856002 PMC12516192

[67] M. Dosch , J. Gerber , F. Jebbawi , and G. Beldi , “Mechanisms of ATP Release by Inflammatory Cells,” International Journal of Molecular Sciences 19 (2018): 1222, 10.3390/ijms19041222. 29669994 PMC5979498

[68] L. Zhang and M. J. Sanderson , “Oscillations in Ciliary Beat Frequency and Intracellular Calcium Concentration in Rabbit Tracheal Epithelial Cells Induced by ATP,” The Journal of Physiology 546 (2003): 733–749, 10.1113/jphysiol.2002.028704. 12563000 PMC2342584

[69] A. S. Shah , Y. Ben‐Shahar , T. O. Moninger , J. N. Kline , and M. J. Welsh , “Motile Cilia of Human Airway Epithelia Are Chemosensory,” Science 325 (2009): 1131–1134, 10.1126/science.1173869. 19628819 PMC2894709

[70] Z. Shan , S. Li , C. Yu , et al., “Embryonic and Skeletal Development of the Albino African Clawed Frog (Xenopus laevis),” Journal of Anatomy 242 (2023): 1051–1066, 10.1111/joa.13835. 36708289 PMC10184547

[71] G. T. Adebogun , et al., “Albino Xenopus Laevis Tadpoles Prefer Dark Environments Compared to Wild Type,” MicroPublication Biology 2023 (2023): 750. 10.17912/micropub.biology.000750 PMC9941856 36824381

[72] J. Tsui , N. Schwartz , and E. S. Ruthazer , “A Developmental Sensitive Period for Spike Timing‐Dependent Plasticity in the Retinotectal Projection,” Frontiers in Synaptic Neuroscience 2 (2010): 13, 10.3389/fnsyn.2010.00013. 21423499 PMC3059707

[73] H. J. Sim , S. Kim , K. Myung , T. Kwon , H. Lee , and T. J. Park , “Xenopus: An Alternative Model System for Identifying Muco‐Active Agents,” PLoS One 13 (2018): 0193310, 10.1371/journal.pone.0193310. PMC5823443 29470529

[74] Á. B. Monteiro , A. F. Alves , A. C. Ribeiro Portela , et al., “Pentylenetetrazole: A Review,” Neurochemistry International 180 (2024): 105841, 10.1016/j.neuint.2024.105841. 39214154

[75] D. A. N. Al‐Halboosi , O. Savchenko , L. K. Heisler , and S. Sylantyev , “Modulation of GABA Release by 5‐HT_1B_ Receptors: An Interplay With AMPA‐Receptors and Voltage‐Gated Ca^2+^ Channels,” Neuropharmacology 241 (2023): 109758, 10.1016/j.neuropharm.2023.109758. 37827445

[76] S. Murthy , M. Niquille , N. Hurni , et al., “Serotonin Receptor 3A Controls Interneuron Migration Into the Neocortex,” Nature Communications 5 (2014): 5524, 10.1038/ncomms6524. PMC4263148 25409778

[77] Y. Wenger , W. Buzgariu , and B. Galliot , “Loss of Neurogenesis in Hydra Leads to Compensatory Regulation of Neurogenic and Neurotransmission Genes in Epithelial Cells,” Philosophical Transactions of the Royal Society B: Biological Sciences 371 (2016): 20150040, 10.1098/rstb.2015.0040. PMC4685579 26598723

[78] H. I. Schreier , Y. Soen , and N. Brenner , “Exploratory Adaptation in Large Random Networks,” Nature Communications 8 (2017): 14826, 10.1038/ncomms14826. PMC5413947 28429717

[79] S. Stern , T. Dror , E. Stolovicki , N. Brenner , and E. Braun , “Genome‐wide Transcriptional Plasticity Underlies Cellular Adaptation to Novel Challenge,” Molecular Systems Biology 3 (2007): MSB4100147, 10.1038/msb4100147. PMC1865588 17453047

[80] E. Braun , “The Unforeseen Challenge: From Genotype‐to‐Phenotype in Cell Populations,” Reports on Progress in Physics 78 (2015): 036602, 10.1088/0034-4885/78/3/036602. 25719211

[81] C. Fields and M. Levin , “Competency in Navigating Arbitrary Spaces as an Invariant for Analyzing Cognition in Diverse Embodiments,” Entropy 24 (2022): 819, 10.3390/e24060819. 35741540 PMC9222757

[82] M. Levin , “Technological Approach to Mind Everywhere: An Experimentally‐Grounded Framework for Understanding Diverse Bodies and Minds,” Frontiers in Systems Neuroscience 16 (2022): 768201, 10.3389/fnsys.2022.768201. 35401131 PMC8988303

[83] J. Schindelin , I. Arganda‐Carreras , E. Frise , et al., “Fiji: An Open‐Source Platform for Biological‐Image Analysis,” Nature Methods 9 (2012): 676–682, 10.1038/nmeth.2019. 22743772 PMC3855844

[84] M. Pachitariu , C. Stringer , M. Dipoppa , et al., “Suite2p: Beyond 10,000 Neurons With Standard Two‐Photon Microscopy,” BioRxiv (2016): 061507, 10.1101/061507.

[85] A. Mortazavi , B. A. Williams , K. McCue , L. Schaeffer , and B. Wold , “Mapping and Quantifying Mammalian Transcriptomes by RNA‐Seq,” Nature Methods 5 (2008): 621–628, 10.1038/nmeth.1226. 18516045 PMC13303166

[86] M. Pertea , G. M. Pertea , C. M. Antonescu , T. Chang , J. T. Mendell , and S. L. Salzberg , “StringTie Enables Improved Reconstruction of a Transcriptome From RNA‐Seq Reads,” Nature Biotechnology 33 (2015): 290–295, 10.1038/nbt.3122. PMC4643835 25690850

[87] Y. Liao , G. K. Smyth , and W. Shi , “featureCounts: An Efficient General Purpose Program for Assigning Sequence Reads to Genomic Features,” Bioinformatics 30 (2014): 923–930, 10.1093/bioinformatics/btt656. 24227677

[88] M. I. Love , W. Huber , and S. Anders , “Moderated Estimation of Fold Change and Dispersion for RNA‐Seq Data With DESeq2,” Genome Biology 15 (2014): 550, 10.1186/s13059-014-0550-8. 25516281 PMC4302049

[89] B. Yates , K. A. Gray , T. E. M. Jones , and E. A. Bruford , “Updates to HCOP: The HGNC Comparison of Orthology Predictions Tool,” Briefings in Bioinformatics 22 (2021): bbab155, 10.1093/bib/bbab155. 33959747 PMC8574622

[90] H. Wickham , Ggplot2: Elegant Graphics for Data Analysis (Springer International Publishing, 2016).

[91] A. Barabási , N. Gulbahce , and J. Loscalzo , “Network Medicine: A Network‐Based Approach to Human Disease,” Nature Reviews Genetics 12 (2011): 56–68, 10.1038/nrg2918. PMC3140052 21164525

[92] W. Nelson , M. Zitnik , B. Wang , J. Leskovec , A. Goldenberg , and R. Sharan , “To Embed or Not: Network Embedding as a Paradigm in Computational Biology,” Frontiers in Genetics 10 (2019): 381, 10.3389/fgene.2019.00381. 31118945 PMC6504708

[93] L. Cantini , E. Medico , S. Fortunato , and M. Caselle , “Detection of Gene Communities in Multi‐Networks Reveals Cancer Drivers,” Scientific Reports 5 (2015): 17386, 10.1038/srep17386. 26639632 PMC4671005

[94] L. Pio‐Lopez , A. Valdeolivas , L. Tichit , É. Remy , and A. Baudot , “MultiVERSE: A Multiplex and Multiplex‐Heterogeneous Network Embedding Approach,” Scientific Reports 11 (2021): 8794, 10.1038/s41598-021-87987-1. 33888761 PMC8062697

[95] R. L. Seal , B. Braschi , K. Gray , et al., “Genenames.Org: The HGNC Resources in 2023,” Nucleic Acids Research 51 (2023): D1003–D1009, 10.1093/nar/gkac888. 36243972 PMC9825485

[96] D. Szklarczyk , R. Kirsch , M. Koutrouli , et al., “The STRING Database in 2023: Protein–Protein Association Networks and Functional Enrichment Analyses for Any Sequenced Genome of Interest,” Nucleic Acids Research 51 (2023): D638–D646, 10.1093/nar/gkac1000. 36370105 PMC9825434

[97] Z. Arendsee , J. Li , U. Singh , A. Seetharam , K. Dorman , and E. S. Wurtele , “Phylostratr: A Framework for Phylostratigraphy,” Bioinformatics 35 (2019): 3617–3627, 10.1093/bioinformatics/btz171. 30873536

