# Supplementary material for: Engineered Living Systems With Self‐Organizing Neural Networks: From Anatomy to Behavior and Gene Expression
Source: Adv Sci (Weinh). 2026 Feb 20;13(28):e08967. doi: 10.1002/advs.202508967 (PMC13185861; doi:10.1002/advs.202508967)
Supplement: Supplementary file 1 — Supporting File 1: advs74389‐sup‐0001‐SuppMat.pdf. [file ADVS-13-e08967-s004.pdf]

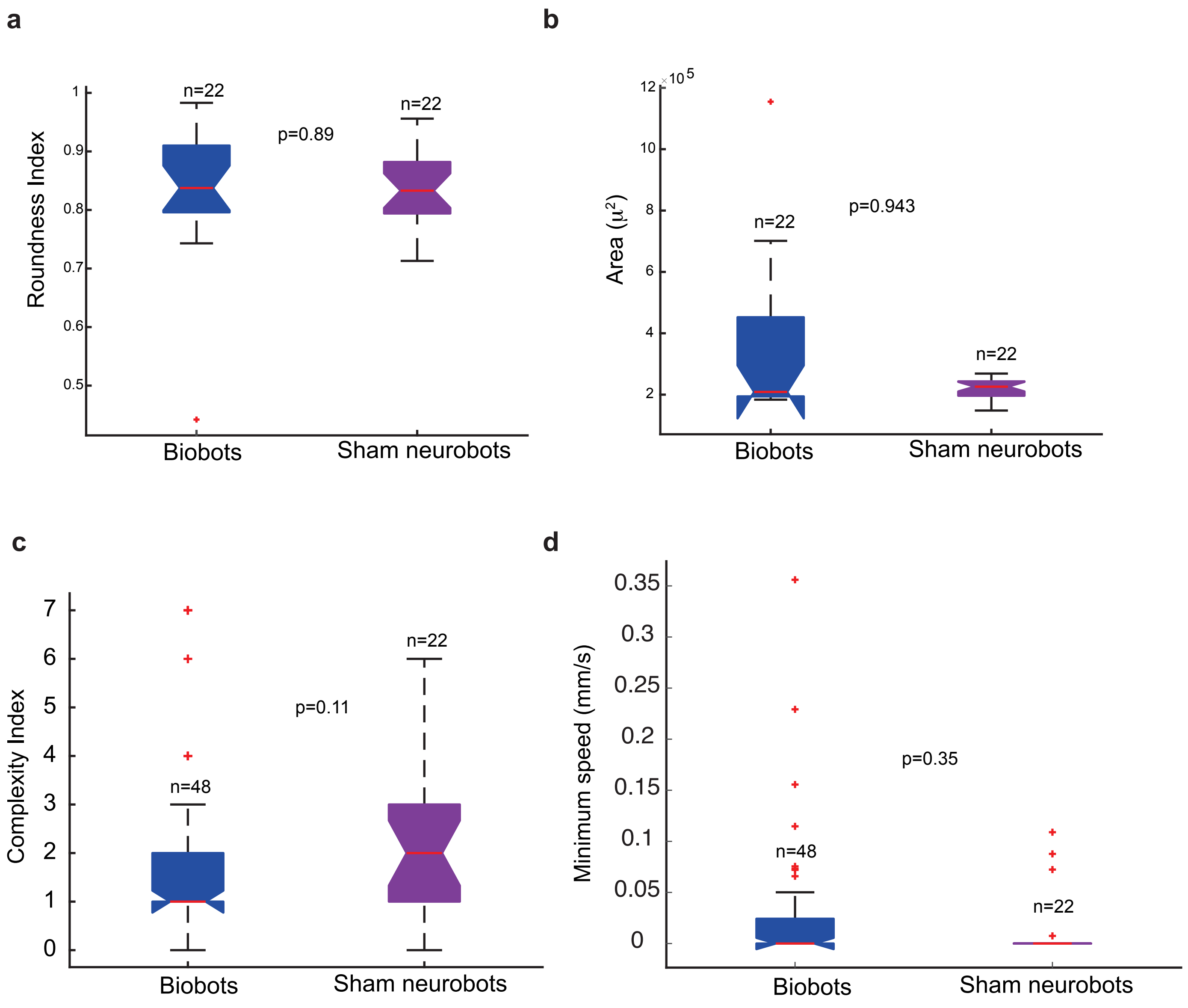

a
Roundness Index
1
0.9
0.8
0.7
0.6
0.5
n=22
n=22
p=0.89
Biobots
Sham neurobots
b
Area (μ²)
×10⁵
12
10
8
6
4
2
n=22
n=22
p=0.943
Biobots
Sham neurobots
c
Complexity Index
7
6
5
4
3
2
1
0
n=48
n=22
p=0.11
Biobots
Sham neurobots
d
Minimum speed (mm/s)
0.35
0.3
0.25
0.2
0.15
0.1
0.05
0
n=48
n=22
p=0.35
Biobots
Sham neurobots

Figure S1

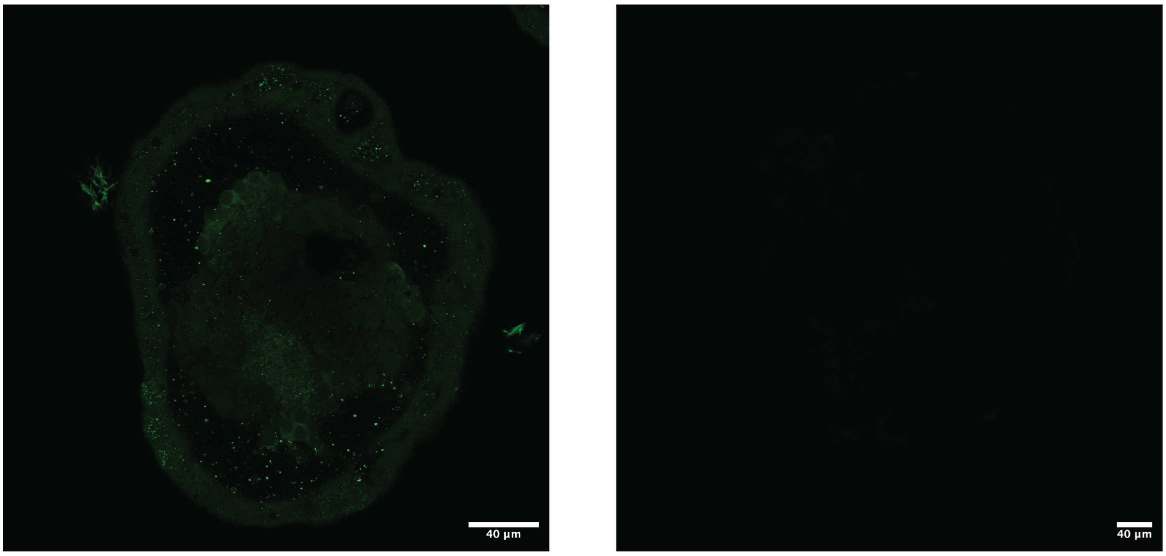

Figure S2

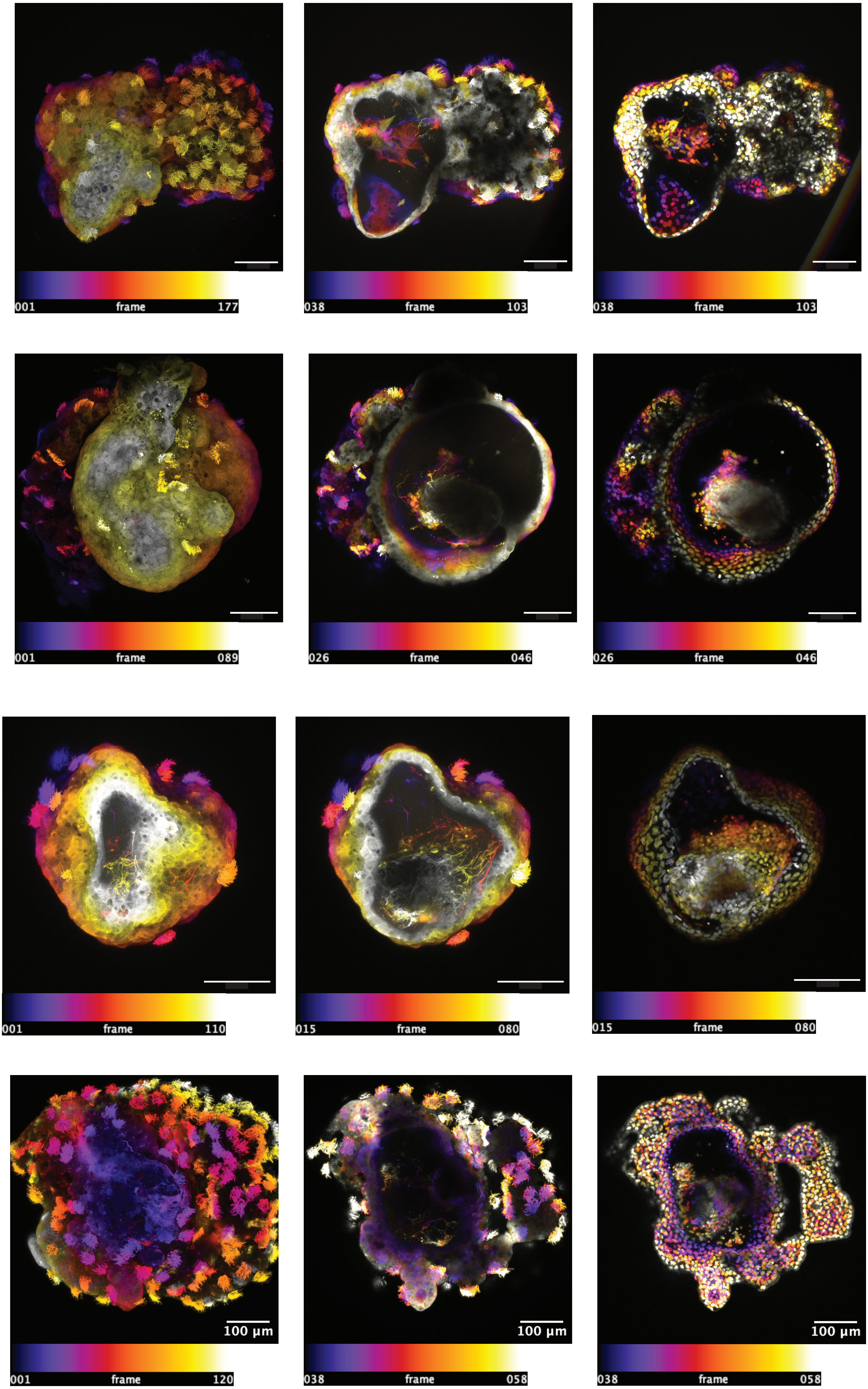

001 frame 177
038 frame 103
038 frame 103
001 frame 089
026 frame 046
026 frame 046
001 frame 110
015 frame 080
015 frame 080
100 µm
001 frame 120
100 µm
038 frame 058
100 µm
038 frame 058

Figure S3

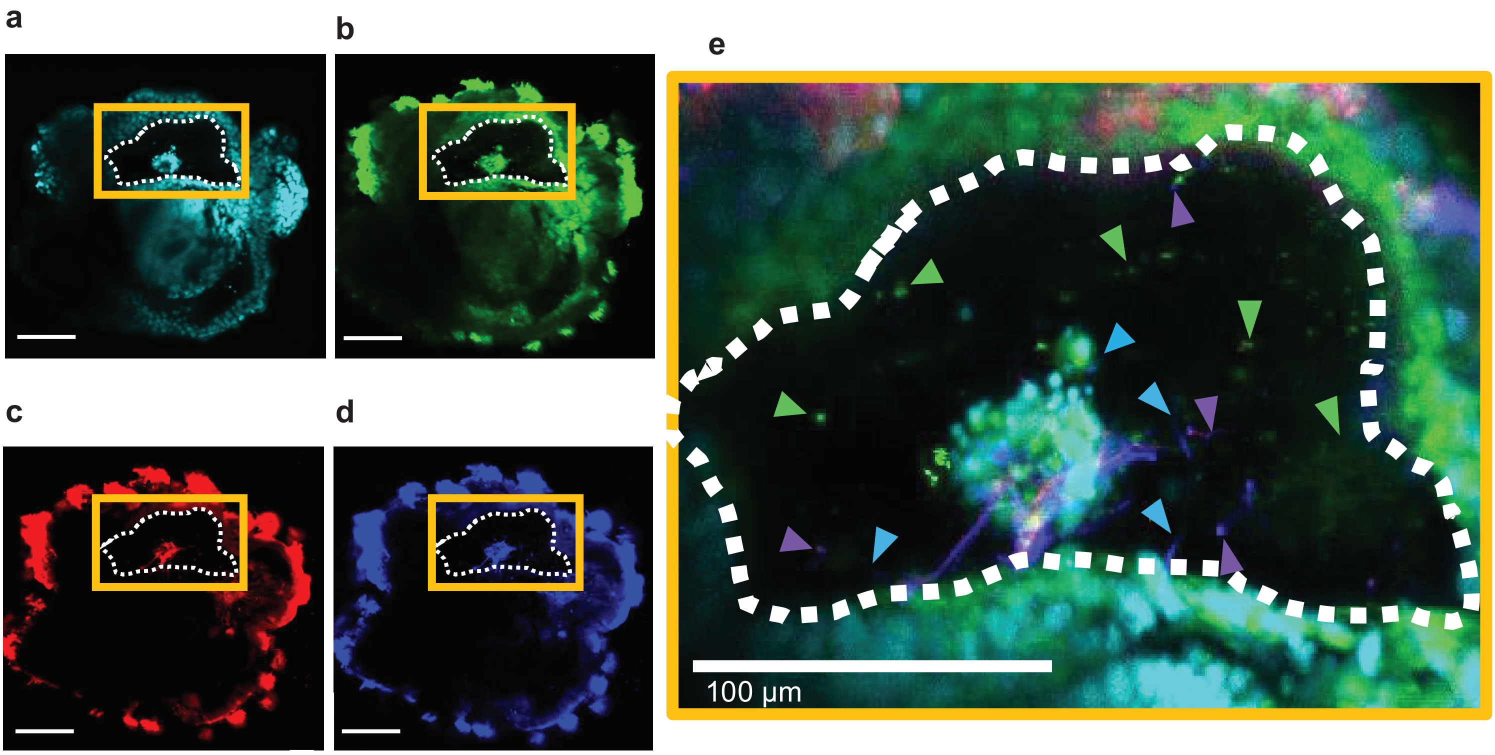

Figure S4

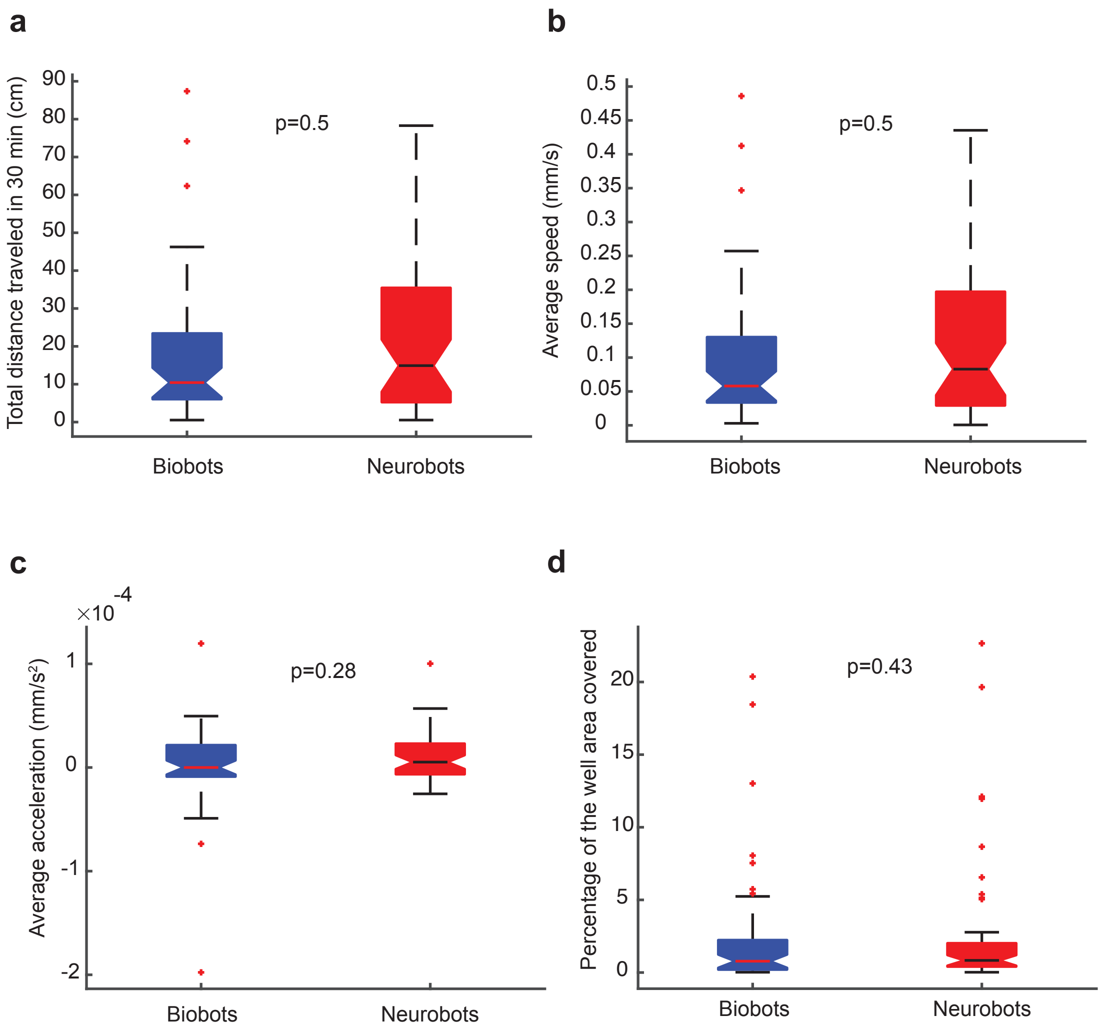
a
Total distance traveled in 30 min (cm)
0
10
20
30
40
50
60
70
80
90
p=0.5
Biobots
Neurobots
b
Average speed (mm/s)
0
0.05
0.1
0.15
0.2
0.25
0.3
0.35
0.4
0.45
0.5
p=0.5
Biobots
Neurobots
c
×10^-4
Average acceleration (mm/s²)
1
0
-1
-2
p=0.28
Biobots
Neurobots
d
Percentage of the well area covered
0
5
10
15
20
p=0.43
Biobots
Neurobots

Figure S5

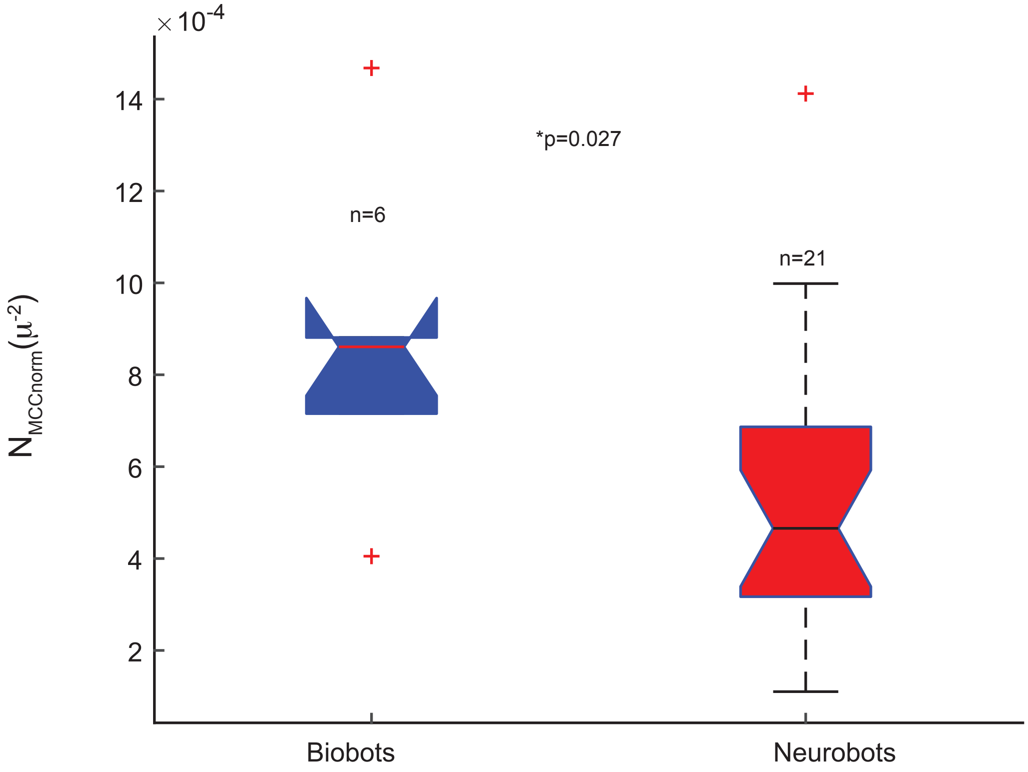

Figure S6

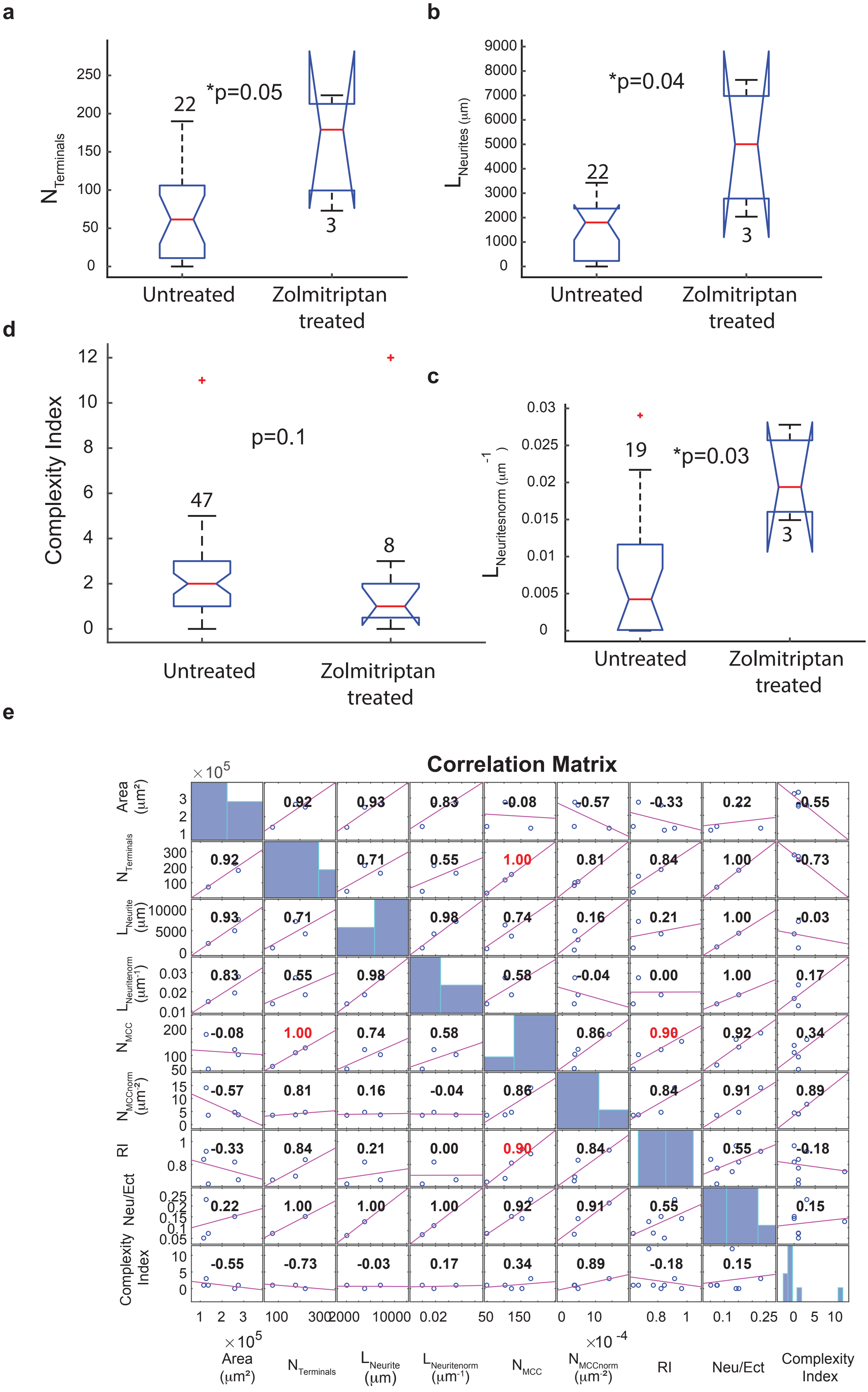
a
b
d
c
e
22
*p=0.05
3
Untreated
Zolmitriptan treated
22
*p=0.04
3
47
p=0.1
8
Complexity Index
19
*p=0.03
3
Correlation Matrix
Area (μm²)
N Terminals
L Neurite (μm)
L Neuritenorm (μm-1)
N MCC
N MCCnorm (μm-2)
RI
Neu/Ect
Complexity Index

Figure S7

a

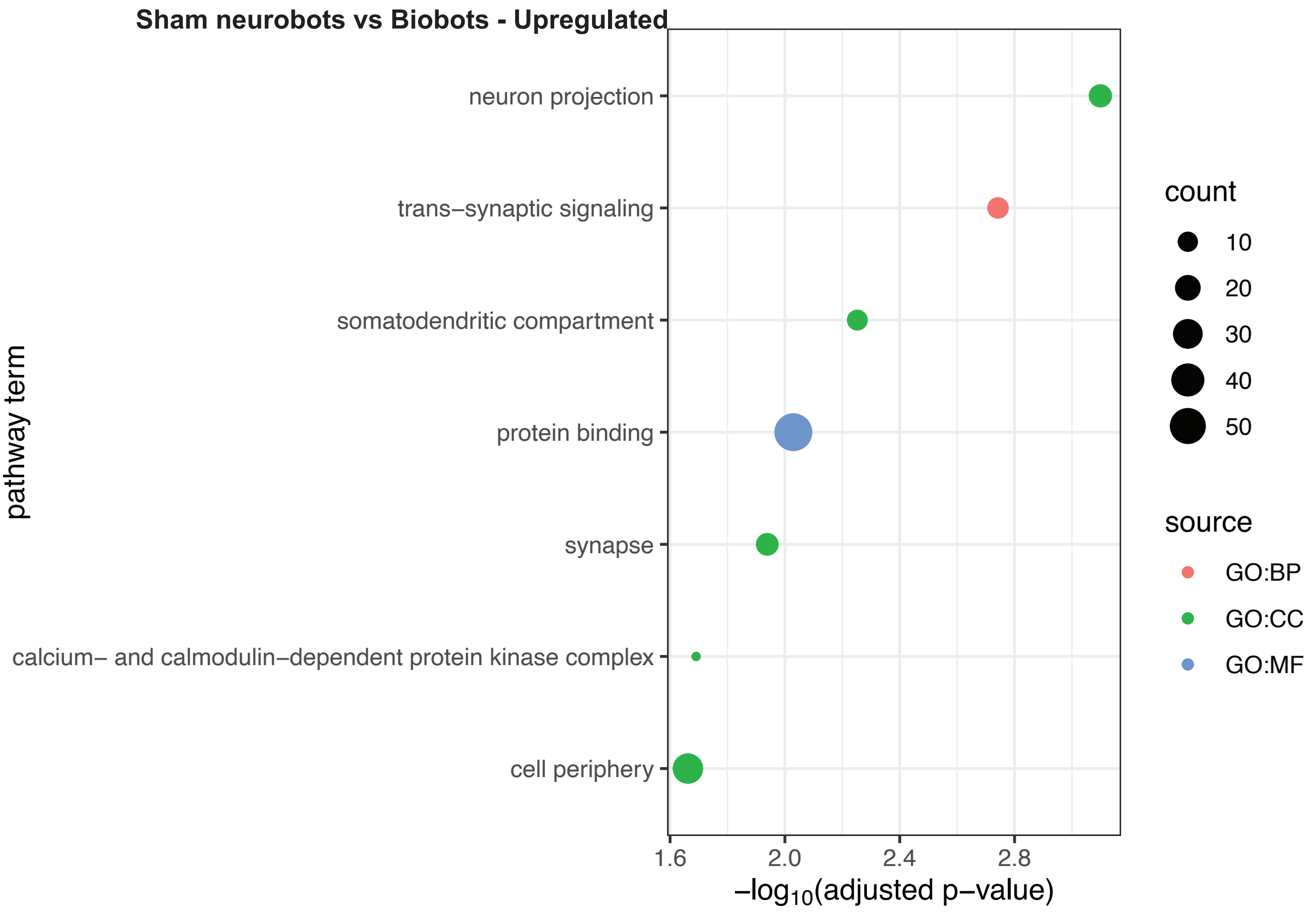

Sham neurobots vs Biobots - Upregulated
neuron projection
trans−synaptic signaling
somatodendritic compartment
protein binding
synapse
calcium− and calmodulin−dependent protein kinase complex
cell periphery
pathway term
1.6
2.0
2.4
2.8
−log10(adjusted p−value)
count
10
20
30
40
50
source
GO:BP
GO:CC
GO:MF

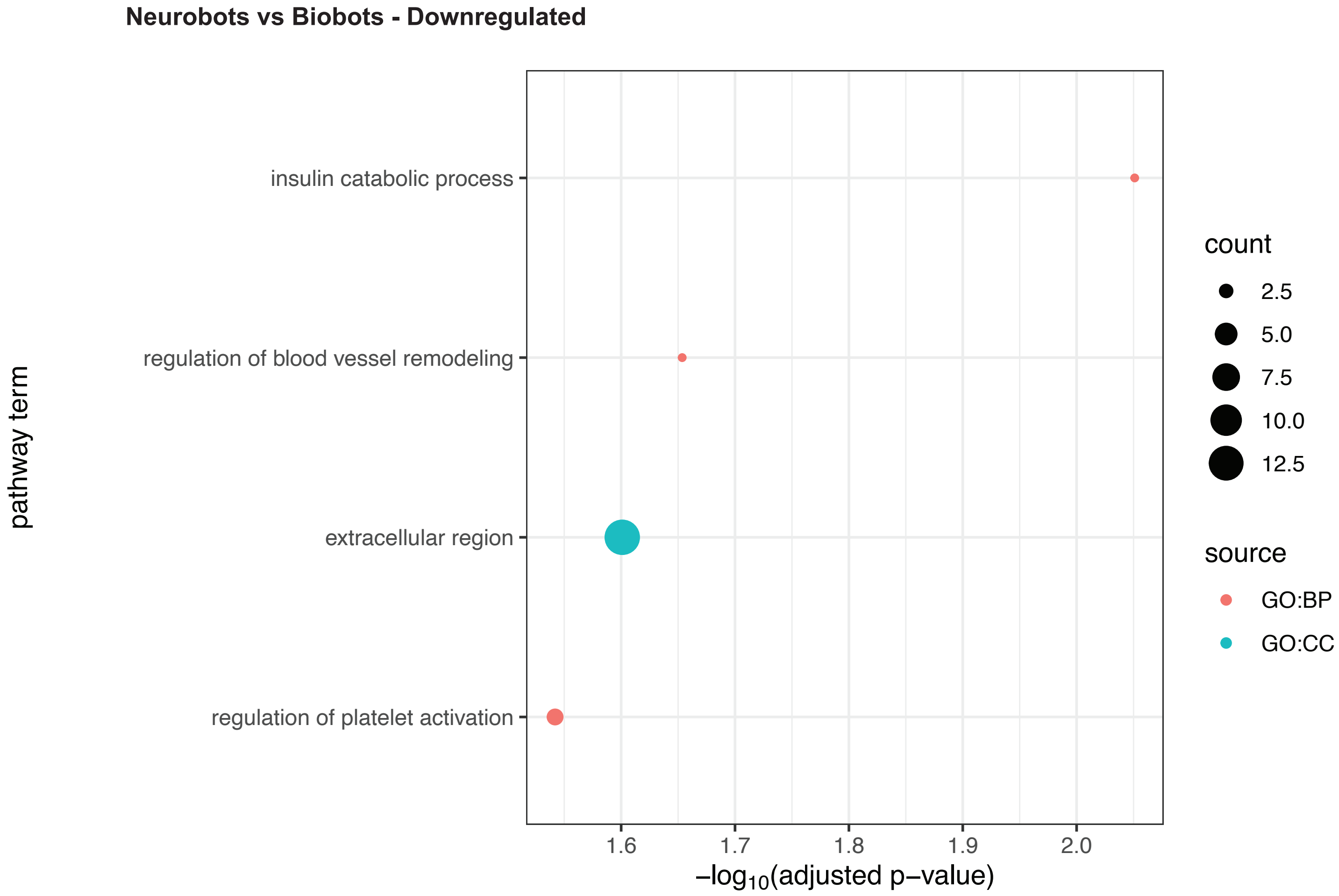

Neurobots vs Biobots - Downregulated
insulin catabolic process
regulation of blood vessel remodeling
extracellular region
regulation of platelet activation
pathway term
1.6
1.7
1.8
1.9
2.0
−log10(adjusted p−value)
count
2.5
5.0
7.5
10.0
12.5
source
GO:BP
GO:CC

Figure S8

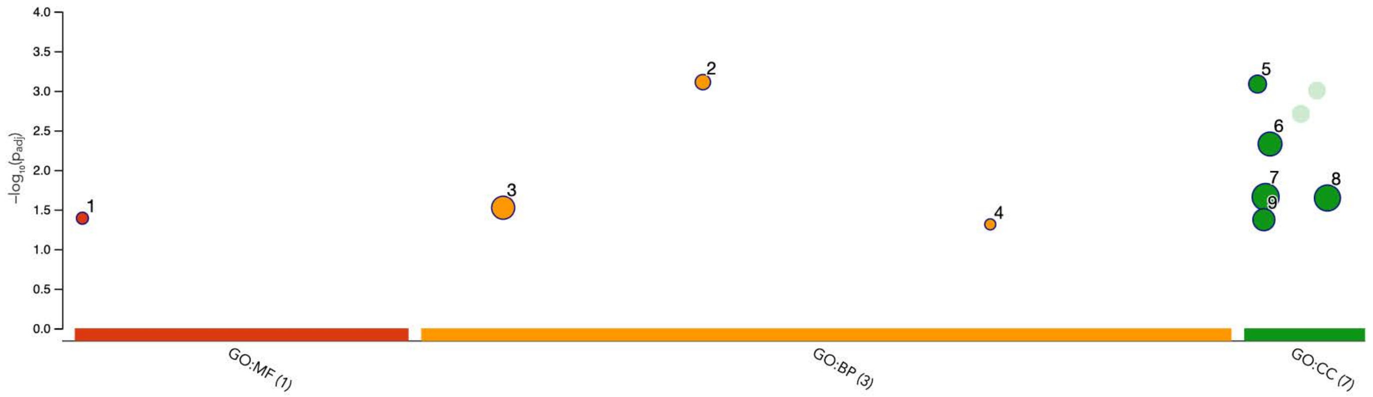

ID	Source	Term ID	Term Name	p_{adj} (query_1)
1	GO:MF	GO:0001849	complement component C1q complex binding	4.066×10^{-2}
2	GO:BP	GO:0035633	maintenance of blood-brain barrier	7.771×10^{-4}
3	GO:BP	GO:0007155	cell adhesion	2.998×10^{-2}
4	GO:BP	GO:0097241	hematopoietic stem cell migration to bone mar...	4.864×10^{-2}
5	GO:CC	GO:0005923	bicellular tight junction	8.224×10^{-4}
6	GO:CC	GO:0030054	cell junction	4.701×10^{-3}
7	GO:CC	GO:0016020	membrane	2.195×10^{-2}
8	GO:CC	GO:0071944	cell periphery	2.260×10^{-2}
9	GO:CC	GO:0009986	cell surface	4.249×10^{-2}

Cluster 2

**version**	e111_eg58_p18_f463989d
**date**	8/26/2024, 6:27:48 PM
**organism**	hsapiens

g:Profiler

Figure S9a

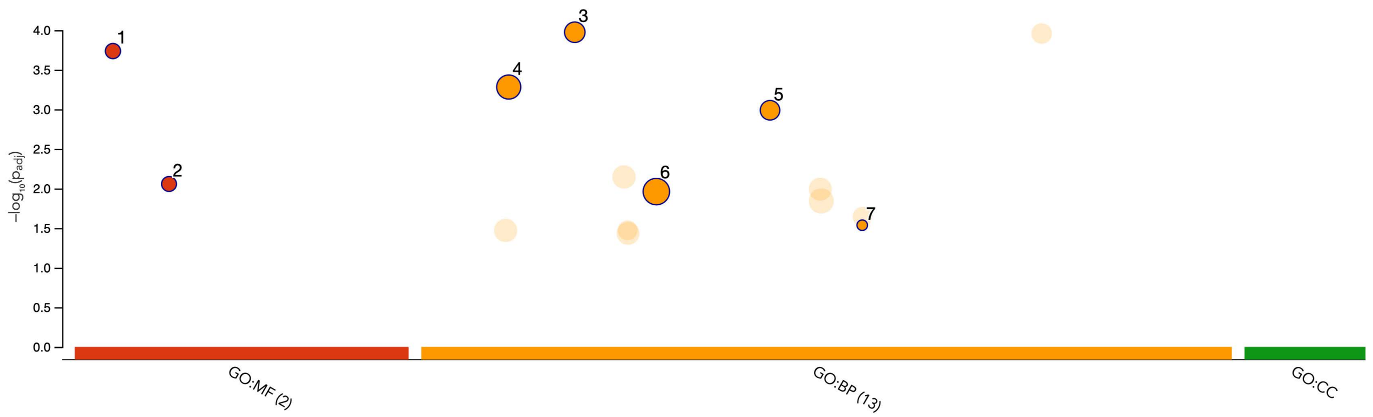

ID	Source	Term ID	Term Name	p_{adj} (query_1)
1	GO:MF	GO:0005109	frizzled binding	1.837×10^{-4}
2	GO:MF	GO:0017147	Wnt-protein binding	8.767×10^{-3}
3	GO:BP	GO:0016055	Wnt signaling pathway	1.063×10^{-4}
4	GO:BP	GO:0007399	nervous system development	5.230×10^{-4}
5	GO:BP	GO:0045165	cell fate commitment	1.027×10^{-3}
6	GO:BP	GO:0032501	multicellular organismal process	1.093×10^{-2}
7	GO:BP	GO:0060061	Spemann organizer formation	2.908×10^{-2}

Cluster 3

**version**	e111_eg58_p18_f463989d
**date**	8/26/2024, 3:56:14 PM
**organism**	hsapiens

g:Profiler

Figure S9b

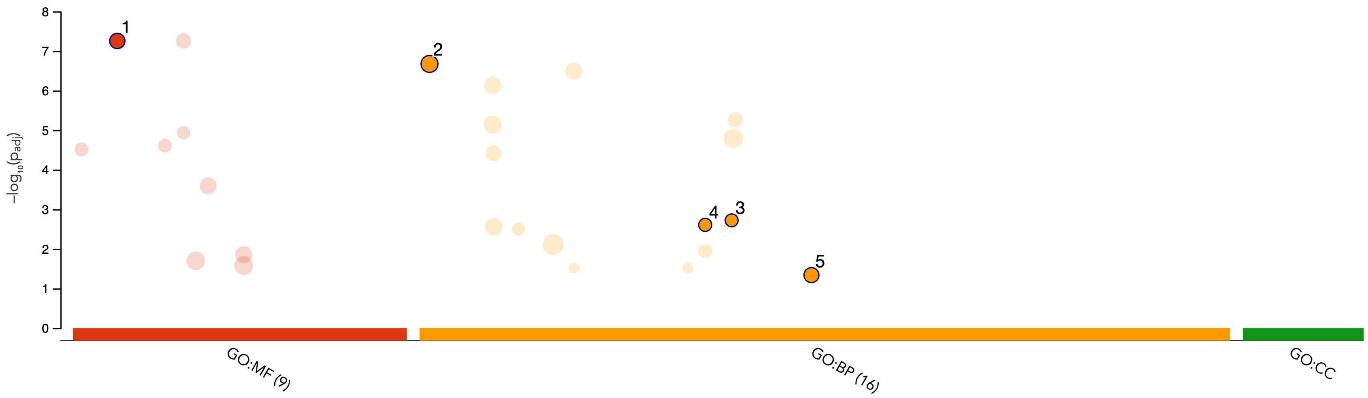

ID	Source	Term ID	Term Name	p_{adj} (query_...
1	GO:MF	GO:0005501	retinoid binding	5.584×10^{-8}
2	GO:BP	GO:0001523	retinoid metabolic process	2.124×10^{-7}
3	GO:BP	GO:0042363	fat-soluble vitamin catabolic process	1.909×10^{-3}
4	GO:BP	GO:0035810	positive regulation of urine volume	2.479×10^{-3}
5	GO:BP	GO:0048384	retinoic acid receptor signaling pathway	4.613×10^{-2}

Cluster 4

**version**	e111_eg58_p18_f463989d
**date**	9/6/2024, 6:36:42 PM
**organism**	hsapiens

g:Profiler

Figure S9c

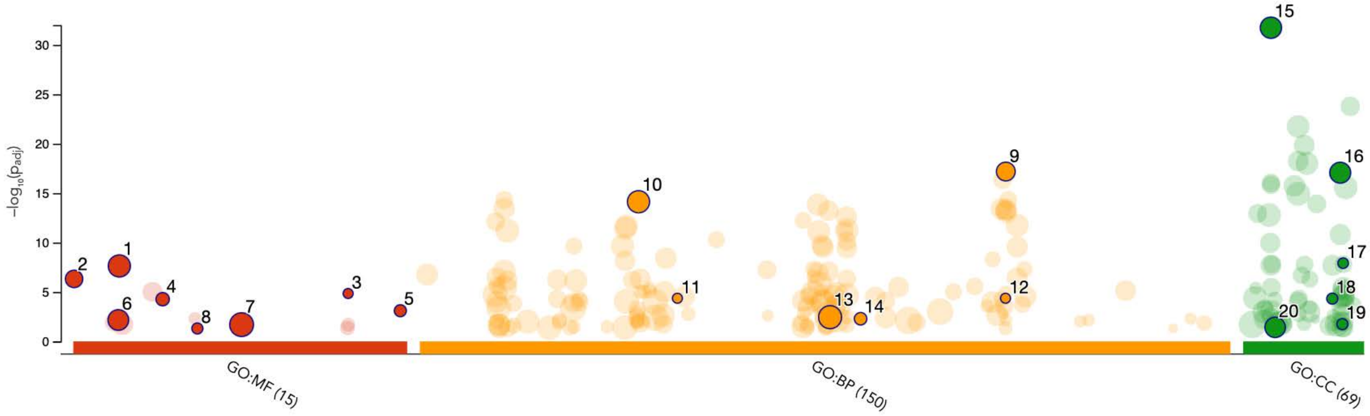

ID	Source	Term ID	Term Name	p_{adj} (query_1)
1	GO:MF	GO:0008092	cytoskeletal protein binding	2.330×10^{-8}
2	GO:MF	GO:0000149	SNARE binding	4.758×10^{-7}
3	GO:MF	GO:0099184	structural constituent of postsynaptic intermediate filament cytoskeleton	1.429×10^{-5}
4	GO:MF	GO:0016812	hydrolase activity, acting on carbon-nitrogen (but not peptide) bonds, in cyclic amides	5.370×10^{-5}
5	GO:MF	GO:1903136	cuprous ion binding	7.920×10^{-4}
6	GO:MF	GO:0005543	phospholipid binding	6.792×10^{-3}
7	GO:MF	GO:0042802	identical protein binding	2.014×10^{-2}
8	GO:MF	GO:0031694	alpha-2A adrenergic receptor binding	4.952×10^{-2}
9	GO:BP	GO:0099504	synaptic vesicle cycle	6.512×10^{-18}
10	GO:BP	GO:0031175	neuron projection development	7.439×10^{-15}
11	GO:BP	GO:0033693	neurofilament bundle assembly	4.327×10^{-5}
12	GO:BP	GO:0099185	postsynaptic intermediate filament cytoskeleton organization	4.327×10^{-5}
13	GO:BP	GO:0050877	nervous system process	3.548×10^{-3}
14	GO:BP	GO:0060052	neurofilament cytoskeleton organization	5.122×10^{-3}
15	GO:CC	GO:0030424	axon	1.837×10^{-32}
16	GO:CC	GO:0098793	presynapse	8.544×10^{-18}
17	GO:CC	GO:0099160	postsynaptic intermediate filament cytoskeleton	1.221×10^{-8}
18	GO:CC	GO:0097418	neurofibrillary tangle	4.716×10^{-5}
19	GO:CC	GO:0099012	neuronal dense core vesicle membrane	1.794×10^{-2}
20	GO:CC	GO:0031252	cell leading edge	3.806×10^{-2}

Cluster 5

**version**	e111_eg58_p18_f463989d
**date**	8/26/2024, 6:29:09 PM
**organism**	hsapiens

g:Profiler

Figure S9d

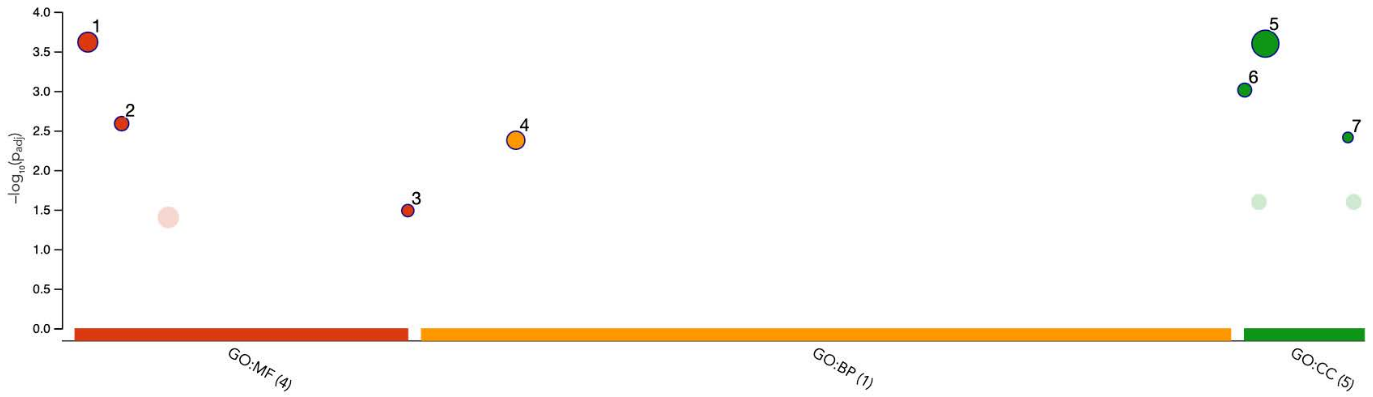

ID	Source	Term ID	Term Name	p_{adj} (query_1)
1	GO:MF	GO:0003924	GTPase activity	2.411×10^{-4}
2	GO:MF	GO:0008157	protein phosphatase 1 binding	2.588×10^{-3}
3	GO:MF	GO:2001069	glycogen binding	3.256×10^{-2}
4	GO:BP	GO:0008277	regulation of G protein-coupled receptor signaling pathway	4.199×10^{-3}
5	GO:CC	GO:0016020	membrane	2.525×10^{-4}
6	GO:CC	GO:0000164	protein phosphatase type 1 complex	9.741×10^{-4}
7	GO:CC	GO:0120216	matrilin complex	3.871×10^{-3}

Cluster 6

**version**	e111_eg58_p18_f463989d
**date**	8/26/2024, 6:29:48 PM
**organism**	hsapiens

g:Profiler

Figure S9e

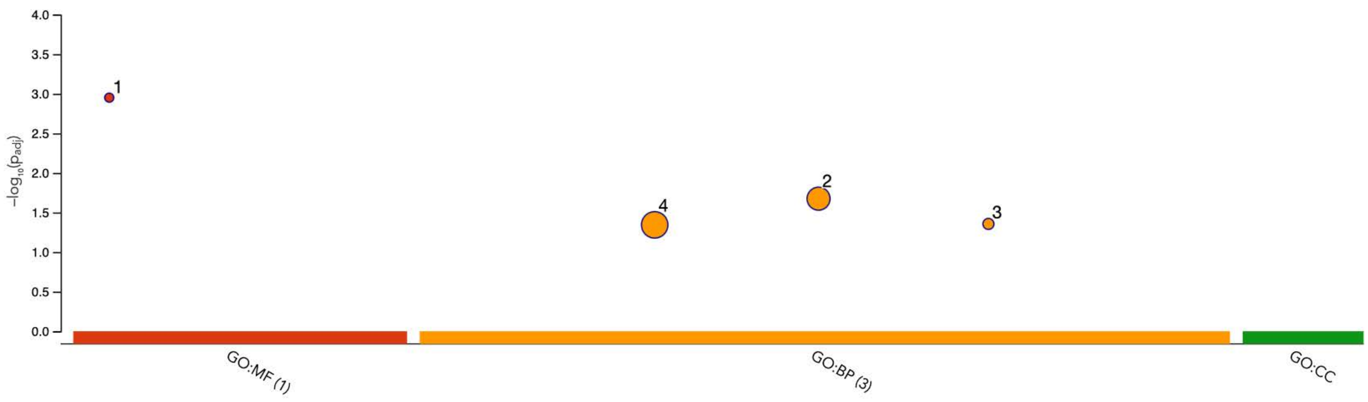

ID	Source	Term ID	Term Name	p_{adj} (query_1)
1	GO:MF	GO:0004968	gonadotropin-releasing hormone receptor activity	1.120×10^{-3}
2	GO:BP	GO:0048699	generation of neurons	2.115×10^{-2}
3	GO:BP	GO:0097211	cellular response to gonadotropin-releasing hormone	4.408×10^{-2}
4	GO:BP	GO:0032501	multicellular organismal process	4.534×10^{-2}

Cluster 8

**version**	e111_eg58_p18_f463989d
**date**	8/26/2024, 6:30:42 PM
**organism**	hsapiens

g:Profiler

Figure S9f

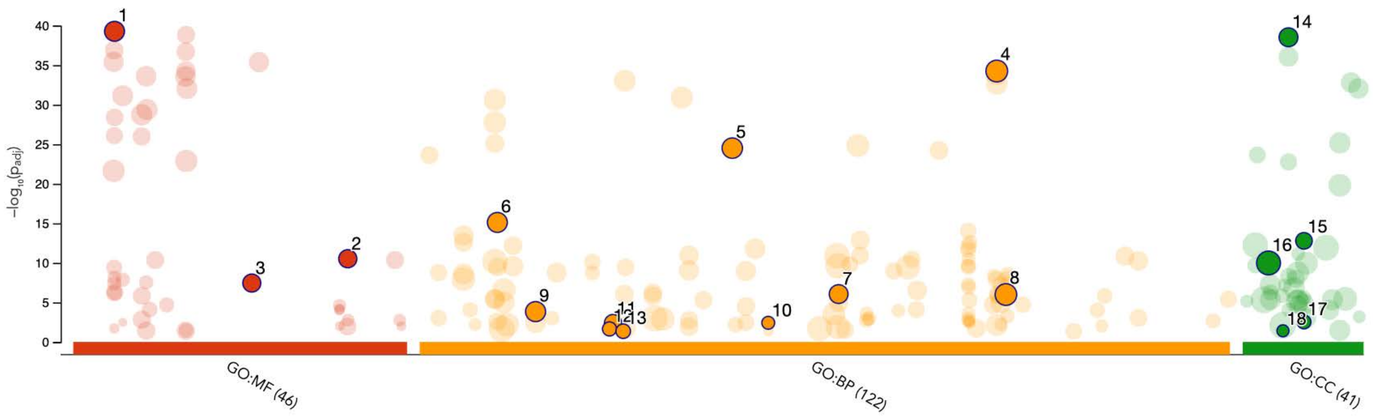

ID	Source	Term ID	Term Name	p_{adj} (query_1)
1	GO:MF	GO:0005261	monoatomic cation channel activity	5.245×10^{-40}
2	GO:MF	GO:0099106	ion channel regulator activity	2.994×10^{-11}
3	GO:MF	GO:0044325	transmembrane transporter binding	3.579×10^{-8}
4	GO:BP	GO:0098662	inorganic cation transmembrane transport	5.555×10^{-35}
5	GO:BP	GO:0042391	regulation of membrane potential	3.102×10^{-25}
6	GO:BP	GO:0006936	muscle contraction	7.677×10^{-16}
7	GO:BP	GO:0051260	protein homooligomerization	8.827×10^{-7}
8	GO:BP	GO:0099537	trans-synaptic signaling	1.011×10^{-6}
9	GO:BP	GO:0010038	response to metal ion	1.462×10^{-4}
10	GO:BP	GO:0045161	neuronal ion channel clustering	3.782×10^{-3}
11	GO:BP	GO:0021675	nerve development	4.125×10^{-3}
12	GO:BP	GO:0021554	optic nerve development	2.254×10^{-2}
13	GO:BP	GO:0022038	corpus callosum development	4.372×10^{-2}
14	GO:CC	GO:0034703	cation channel complex	2.989×10^{-39}
15	GO:CC	GO:0044304	main axon	1.640×10^{-13}
16	GO:CC	GO:0030054	cell junction	1.030×10^{-10}
17	GO:CC	GO:0044305	calyx of Held	2.989×10^{-3}
18	GO:CC	GO:0033010	paranodal junction	3.884×10^{-2}

Cluster 9

version e111_eg58_p18_f463989d
date 8/26/2024, 6:31:40 PM
organism hsapiens

g:Profiler

Figure S9g

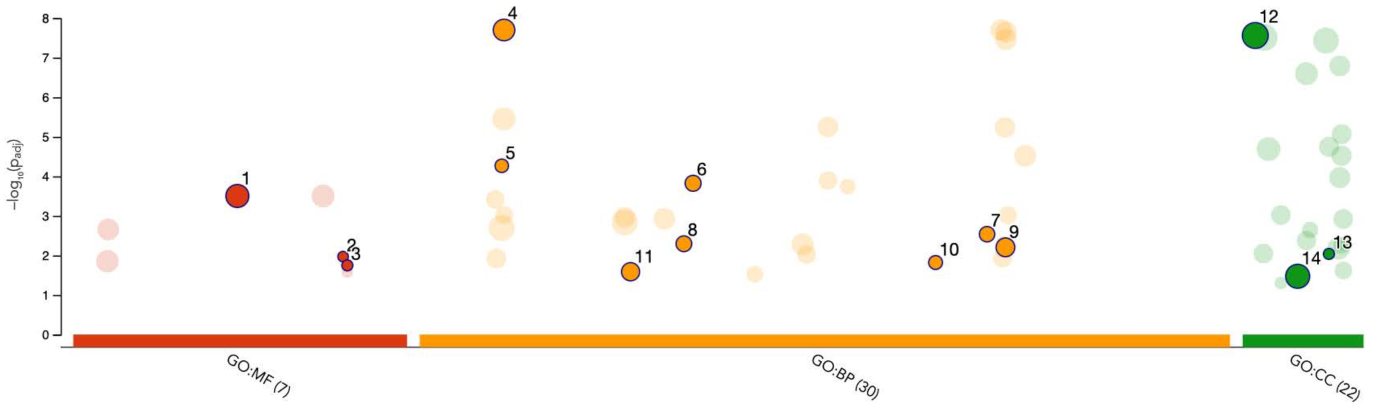

ID	Source	Term ID	Term Name	p_{adj} (query_1)
1	GO:MF	GO:0038023	signaling receptor activity	3.139×10^{-4}
2	GO:MF	GO:0097016	L27 domain binding	1.073×10^{-2}
3	GO:MF	GO:0098919	structural constituent of postsynaptic density	1.785×10^{-2}
4	GO:BP	GO:0007268	chemical synaptic transmission	1.998×10^{-8}
5	GO:BP	GO:0007158	neuron cell-cell adhesion	5.415×10^{-5}
6	GO:BP	GO:0035176	social behavior	1.500×10^{-4}
7	GO:BP	GO:0097106	postsynaptic density organization	2.903×10^{-3}
8	GO:BP	GO:0034331	cell junction maintenance	5.068×10^{-3}
9	GO:BP	GO:0099504	synaptic vesicle cycle	6.241×10^{-3}
10	GO:BP	GO:0071625	vocalization behavior	1.508×10^{-2}
11	GO:BP	GO:0030534	adult behavior	2.586×10^{-2}
12	GO:CC	GO:0005886	plasma membrane	2.749×10^{-8}
13	GO:CC	GO:0097025	MPP7-DLG1-LIN7 complex	9.148×10^{-3}
14	GO:CC	GO:0042995	cell projection	3.369×10^{-2}

Cluster 10

**version**	e111_eg58_p18_f463989d
**date**	8/26/2024, 6:33:19 PM
**organism**	hsapiens

g:Profiler

Figure S9h

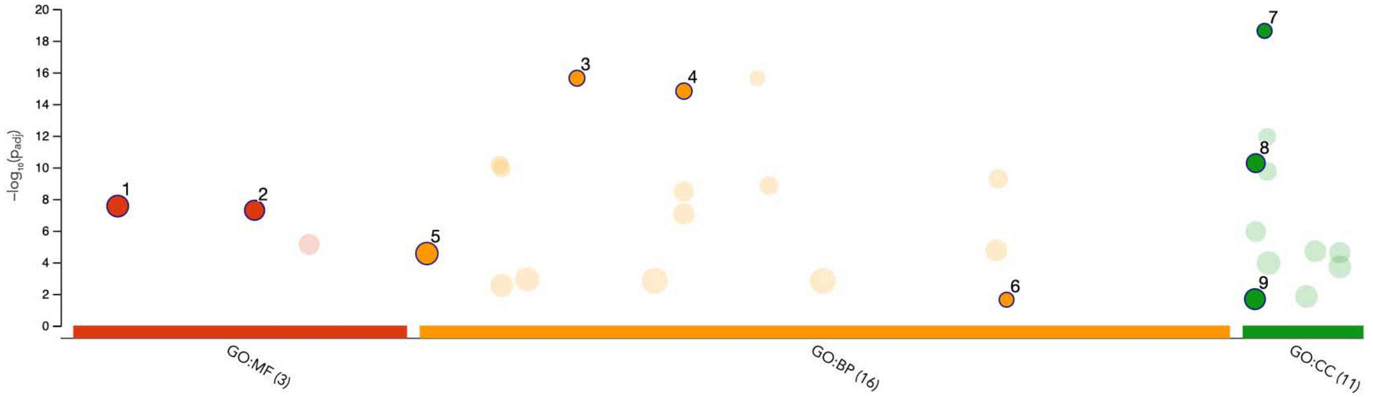

ID	Source	Term ID	Term Name	p_{adj} (query_1)
1	GO:MF	GO:0005509	calcium ion binding	2.799×10^{-8}
2	GO:MF	GO:0045296	cadherin binding	5.126×10^{-8}
3	GO:BP	GO:0016339	calcium-dependent cell-cell adhesion via plasma membrane cell adhesion molecules	2.319×10^{-16}
4	GO:BP	GO:0034332	adherens junction organization	1.517×10^{-15}
5	GO:BP	GO:0000902	cell morphogenesis	2.781×10^{-5}
6	GO:BP	GO:0099560	synaptic membrane adhesion	2.316×10^{-2}
7	GO:CC	GO:0016342	catenin complex	2.373×10^{-19}
8	GO:CC	GO:0005912	adherens junction	5.419×10^{-11}
9	GO:CC	GO:0005874	microtubule	2.126×10^{-2}

Cluster 11

**version**	e111_eg58_p18_f463989d
**date**	8/26/2024, 6:32:43 PM
**organism**	hsapiens

g:Profiler

Figure S9i

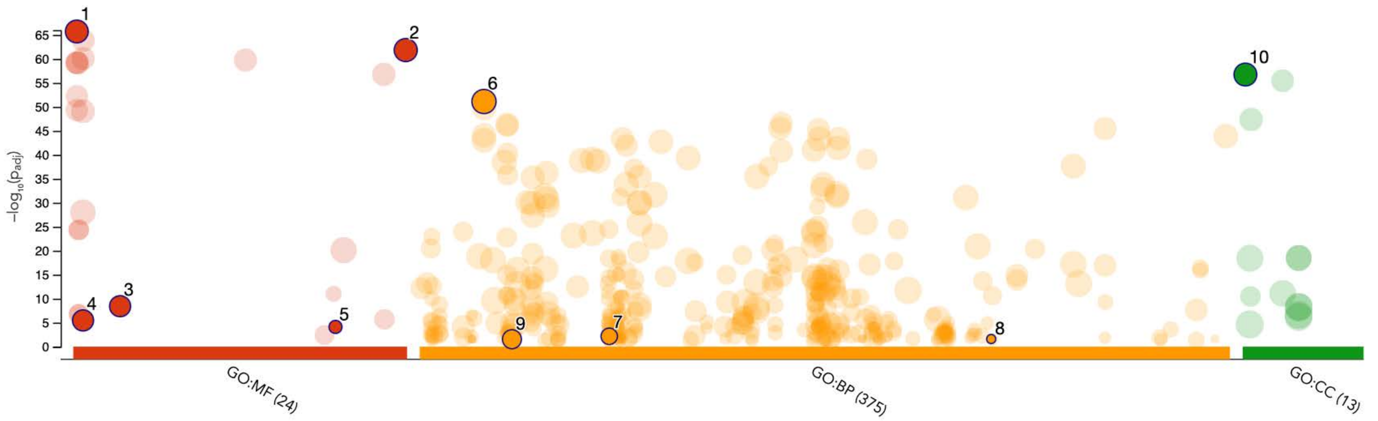

ID	Source	Term ID	Term Name	p_{adj} (query_1)
1	GO:MF	GO:0000981	DNA-binding transcription factor activity, RNA polymerase II-specific	1.896×10^{-66}
2	GO:MF	GO:1990837	sequence-specific double-stranded DNA binding	1.439×10^{-62}
3	GO:MF	GO:0008134	transcription factor binding	3.743×10^{-9}
4	GO:MF	GO:0003682	chromatin binding	3.416×10^{-6}
5	GO:MF	GO:0071837	HMG box domain binding	7.655×10^{-5}
6	GO:BP	GO:0006357	regulation of transcription by RNA polymerase II	7.619×10^{-52}
7	GO:BP	GO:0021545	cranial nerve development	6.963×10^{-3}
8	GO:BP	GO:0097378	dorsal spinal cord interneuron axon guidance	2.578×10^{-2}
9	GO:BP	GO:0007601	visual perception	2.868×10^{-2}
10	GO:CC	GO:0000785	chromatin	1.833×10^{-57}

Cluster 12

version e111_eg58_p18_f463989d
date 8/26/2024, 6:33:49 PM
organism hsapiens

g:Profiler

Figure S9j

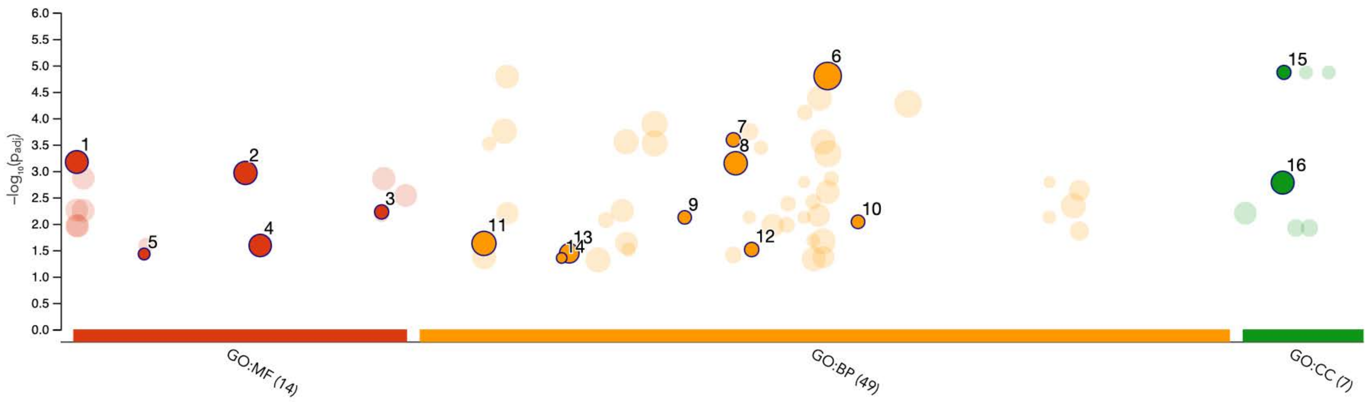

ID	Source	Term ID	Term Name	p_{adj} (query_1)
1	GO:MF	GO:0000981	DNA-binding transcription factor activity, RNA polymerase II-specific	6.750×10^{-4}
2	GO:MF	GO:0043565	sequence-specific DNA binding	1.086×10^{-3}
3	GO:MF	GO:0120020	cholesterol transfer activity	5.955×10^{-3}
4	GO:MF	GO:0046983	protein dimerization activity	2.574×10^{-2}
5	GO:MF	GO:0015168	glycerol transmembrane transporter activity	3.740×10^{-2}
6	GO:BP	GO:0050789	regulation of biological process	1.585×10^{-5}
7	GO:BP	GO:0042438	melanin biosynthetic process	2.568×10^{-4}
8	GO:BP	GO:0042592	homeostatic process	7.129×10^{-4}
9	GO:BP	GO:0034375	high-density lipoprotein particle remodeling	7.568×10^{-3}
10	GO:BP	GO:0055091	phospholipid homeostasis	9.177×10^{-3}
11	GO:BP	GO:0006357	regulation of transcription by RNA polymerase II	2.354×10^{-2}
12	GO:BP	GO:0043576	regulation of respiratory gaseous exchange	3.070×10^{-2}
13	GO:BP	GO:0015850	organic hydroxy compound transport	3.618×10^{-2}
14	GO:BP	GO:0014826	vein smooth muscle contraction	4.439×10^{-2}
15	GO:CC	GO:0033162	melanosome membrane	1.356×10^{-5}
16	GO:CC	GO:0032993	protein-DNA complex	1.661×10^{-3}

Cluster 13

version e111_eg58_p18_f463989d

date 8/26/2024, 6:34:23 PM

organism hsapiens

g:Profiler

Figure S9k

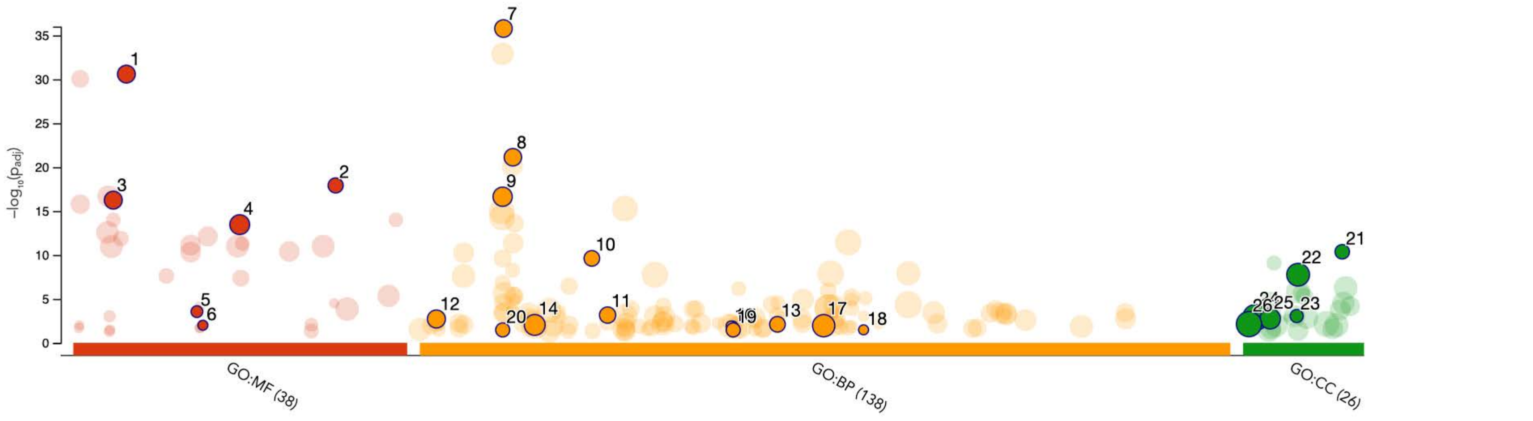

ID	Source	Term ID	Term Name	p_{adj} (query_1)
1	GO:MF	GO:0008528	G protein-coupled peptide receptor activity	2.569×10^{-31}
2	GO:MF	GO:0071855	neuropeptide receptor binding	1.210×10^{-18}
3	GO:MF	GO:0005179	hormone activity	5.682×10^{-17}
4	GO:MF	GO:0042277	peptide binding	3.494×10^{-14}
5	GO:MF	GO:0031628	opioid receptor binding	2.798×10^{-4}
6	GO:MF	GO:0031893	vasopressin receptor binding	9.945×10^{-3}
7	GO:BP	GO:0007218	neuropeptide signaling pathway	1.666×10^{-36}
8	GO:BP	GO:0007631	feeding behavior	7.475×10^{-22}
9	GO:BP	GO:0007188	adenylate cyclase-modulating G protein-coupled receptor signaling pathway	2.323×10^{-17}
10	GO:BP	GO:0019098	reproductive behavior	2.437×10^{-10}
11	GO:BP	GO:0019933	cAMP-mediated signaling	6.923×10^{-4}
12	GO:BP	GO:0001936	regulation of endothelial cell proliferation	1.933×10^{-3}
13	GO:BP	GO:0045761	regulation of adenylate cyclase activity	7.763×10^{-3}
14	GO:BP	GO:0009991	response to extracellular stimulus	9.213×10^{-3}
15	GO:BP	GO:0042418	epinephrine biosynthetic process	1.110×10^{-2}
16	GO:BP	GO:0042309	homoiothermy	1.110×10^{-2}
17	GO:BP	GO:0048878	chemical homeostasis	1.120×10^{-2}
18	GO:BP	GO:0060183	apelin receptor signaling pathway	3.326×10^{-2}
19	GO:BP	GO:0042423	catecholamine biosynthetic process	3.403×10^{-2}
20	GO:BP	GO:0007190	activation of adenylate cyclase activity	3.403×10^{-2}
21	GO:CC	GO:0098992	neuronal dense core vesicle	4.300×10^{-11}
22	GO:CC	GO:0043005	neuron projection	1.754×10^{-8}
23	GO:CC	GO:0042583	chromaffin granule	8.911×10^{-4}
24	GO:CC	GO:0005886	plasma membrane	1.260×10^{-3}
25	GO:CC	GO:0030133	transport vesicle	1.826×10^{-3}
26	GO:CC	GO:0005576	extracellular region	7.429×10^{-3}

Cluster 14

version e111_eg58_p18_f463989d
date 8/26/2024, 6:34:55 PM
organism hsapiens

g:Profiler

Figure S9I

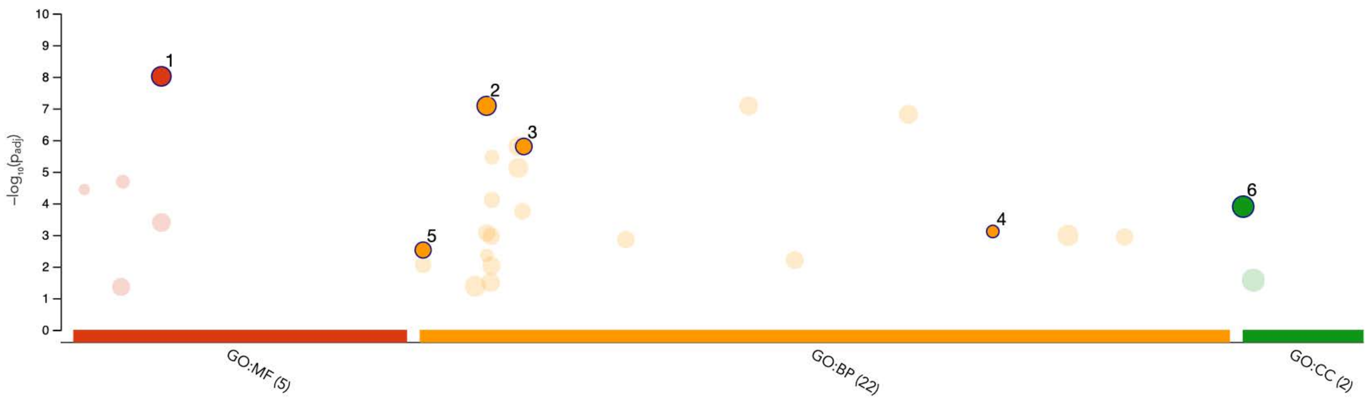

ID	Source	Term ID	Term Name	p_{adj} (query_1)
1	GO:MF	GO:0016757	glycosyltransferase activity	9.660×10^{-9}
2	GO:BP	GO:0006486	protein glycosylation	8.254×10^{-8}
3	GO:BP	GO:0009311	oligosaccharide metabolic process	1.594×10^{-6}
4	GO:BP	GO:0097503	sialylation	7.763×10^{-4}
5	GO:BP	GO:0000381	regulation of alternative mRNA splicing, via spliceosome	2.998×10^{-3}
6	GO:CC	GO:0000139	Golgi membrane	1.274×10^{-4}

Cluster 16

**version**	e111_eg58_p18_f463989d
**date**	8/26/2024, 6:36:06 PM
**organism**	hsapiens

g:Profiler

Figure S9m

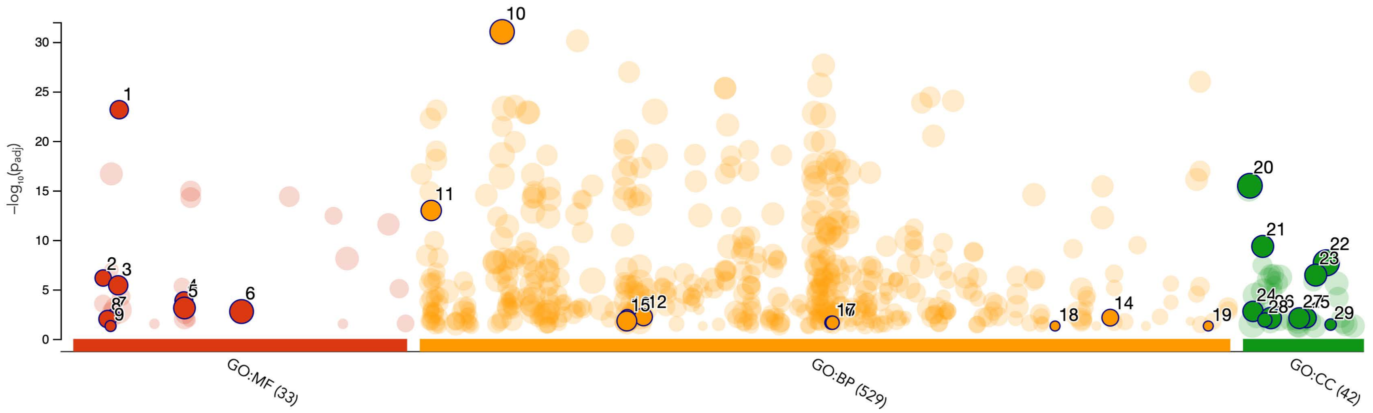

ID	Source	Term ID	Term Name	p_{adj} (query_...
1	GO:MF	GO:0008083	growth factor activity	6.958×10^{-24}
2	GO:MF	GO:0004714	transmembrane receptor protein tyrosine kinase activity	7.050×10^{-7}
3	GO:MF	GO:0005539	glycosaminoglycan binding	3.860×10^{-6}
4	GO:MF	GO:0019838	growth factor binding	1.390×10^{-4}
5	GO:MF	GO:0019900	kinase binding	7.505×10^{-4}
6	GO:MF	GO:0042802	identical protein binding	1.715×10^{-3}
7	GO:MF	GO:0005172	vascular endothelial growth factor receptor binding	4.502×10^{-3}
8	GO:MF	GO:0004896	cytokine receptor activity	9.862×10^{-3}
9	GO:MF	GO:0005030	neurotrophin receptor activity	4.979×10^{-2}
10	GO:BP	GO:0007166	cell surface receptor signaling pathway	9.377×10^{-32}
11	GO:BP	GO:0001667	ameboidal-type cell migration	1.062×10^{-13}
12	GO:BP	GO:0031623	receptor internalization	5.856×10^{-3}
13	GO:BP	GO:0030212	hyaluronan metabolic process	6.146×10^{-3}
14	GO:BP	GO:1902893	regulation of miRNA transcription	7.110×10^{-3}
15	GO:BP	GO:0030198	extracellular matrix organization	1.554×10^{-2}
16	GO:BP	GO:0050930	induction of positive chemotaxis	2.307×10^{-2}
17	GO:BP	GO:0050966	detection of mechanical stimulus involved in sensory perception of pain	2.307×10^{-2}
18	GO:BP	GO:1900625	positive regulation of monocyte aggregation	4.974×10^{-2}
19	GO:BP	GO:2000446	regulation of macrophage migration inhibitory factor signaling pathway	4.974×10^{-2}
20	GO:CC	GO:0005615	extracellular space	3.354×10^{-16}
21	GO:CC	GO:0009986	cell surface	4.504×10^{-10}
22	GO:CC	GO:0071944	cell periphery	2.080×10^{-8}
23	GO:CC	GO:0070161	anchoring junction	3.567×10^{-7}
24	GO:CC	GO:0005769	early endosome	1.653×10^{-3}
25	GO:CC	GO:0045121	membrane raft	7.652×10^{-3}
26	GO:CC	GO:0030424	axon	7.840×10^{-3}
27	GO:CC	GO:0043235	receptor complex	7.951×10^{-3}
28	GO:CC	GO:0016327	apicolateral plasma membrane	1.211×10^{-2}
29	GO:CC	GO:0097180	serine protease inhibitor complex	3.560×10^{-2}

Cluster 17

version e111_eg58_p18_f463989d
date 9/6/2024, 6:37:45 PM
organism hsapiens

g:Profiler

Figure S9n

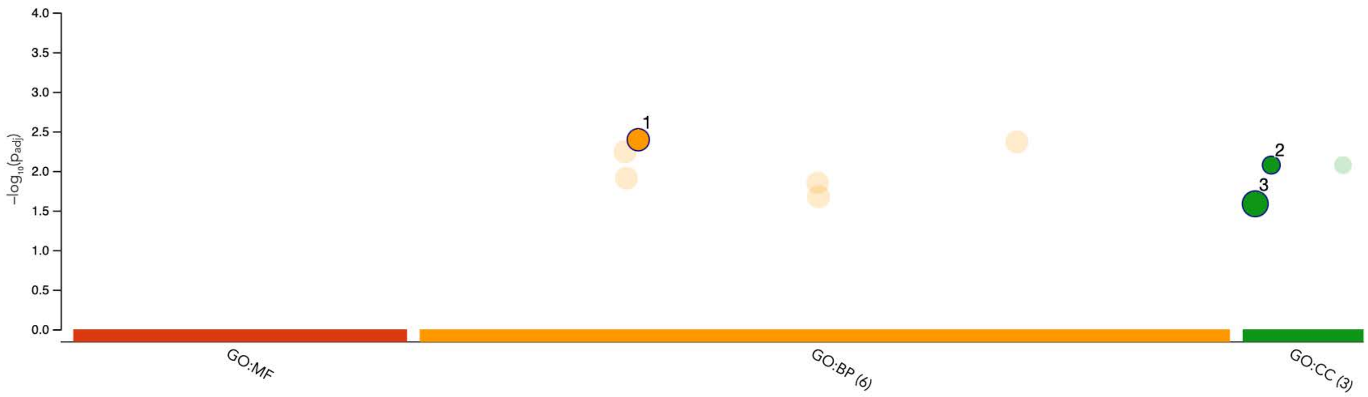

ID	Source	Term ID	Term Name	p_{adj} (query_1)
1	GO:BP	GO:0031175	neuron projection development	4.034×10^{-3}
2	GO:CC	GO:0030672	synaptic vesicle membrane	8.425×10^{-3}
3	GO:CC	GO:0005886	plasma membrane	2.600×10^{-2}

Cluster 18

version e111_eg58_p18_f463989d
date 8/26/2024, 6:36:40 PM
organism hsapiens

g:Profiler

Figure S9o

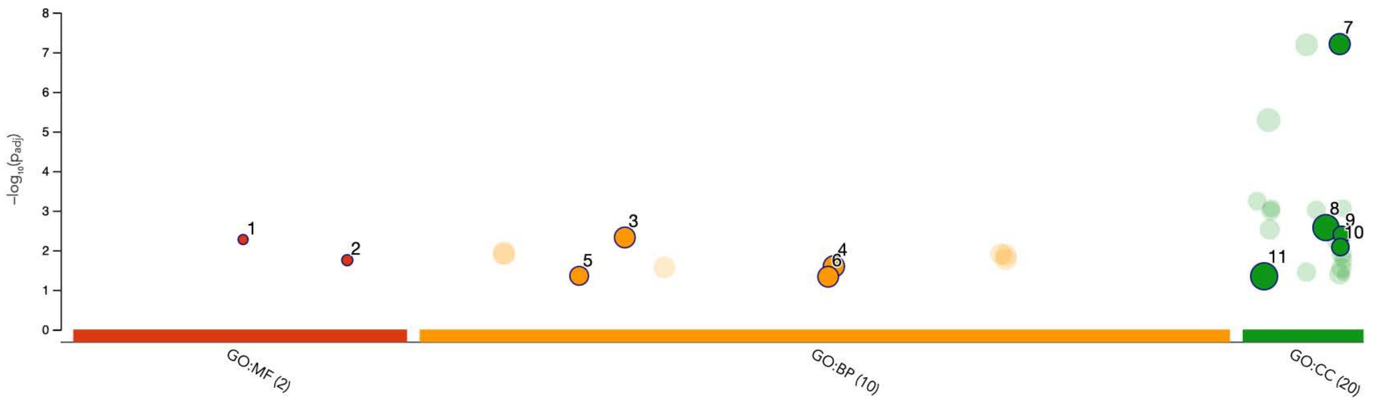

ID	Source	Term ID	Term Name	p_{adj} (query_1)
1	GO:MF	GO:0042988	X11-like protein binding	5.351×10^{-3}
2	GO:MF	GO:0098882	structural constituent of presynaptic active zone	1.779×10^{-2}
3	GO:BP	GO:0023061	signal release	4.751×10^{-3}
4	GO:BP	GO:0051046	regulation of secretion	2.549×10^{-2}
5	GO:BP	GO:0017157	regulation of exocytosis	4.417×10^{-2}
6	GO:BP	GO:0050808	synapse organization	4.648×10^{-2}
7	GO:CC	GO:0098793	presynapse	6.223×10^{-8}
8	GO:CC	GO:0071944	cell periphery	2.674×10^{-3}
9	GO:CC	GO:0098982	GABA-ergic synapse	4.017×10^{-3}
10	GO:CC	GO:0098839	postsynaptic density membrane	8.309×10^{-3}
11	GO:CC	GO:0016020	membrane	4.537×10^{-2}

Cluster 19

**version**	e111_eg58_p18_f463989d
**date**	8/26/2024, 6:37:06 PM
**organism**	hsapiens

g:Profiler

Figure S9p

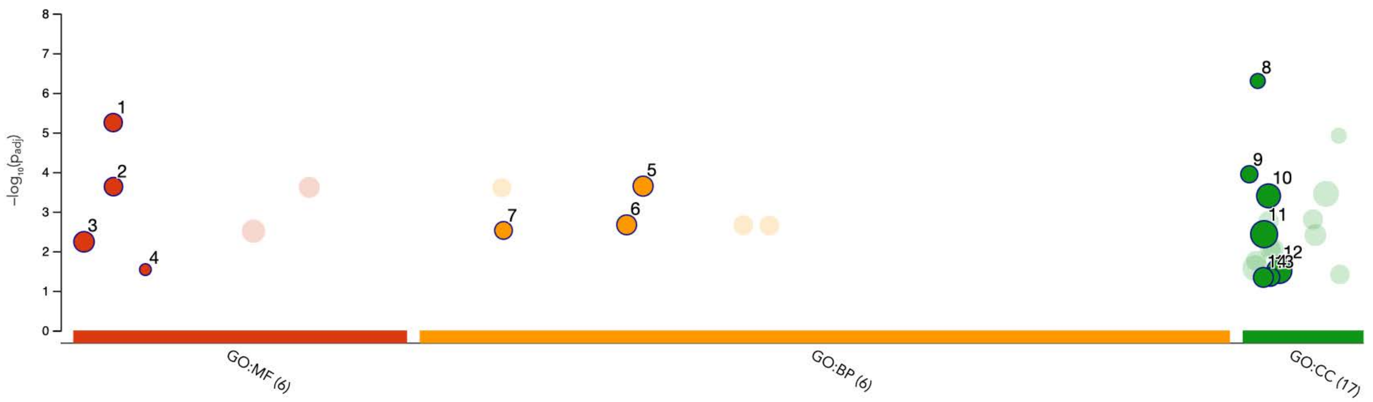

ID	Source	Term ID	Term Name	p_{adj} (query_1)
1	GO:MF	GO:0005178	integrin binding	5.640×10^{-6}
2	GO:MF	GO:0005201	extracellular matrix structural constituent	2.337×10^{-4}
3	GO:MF	GO:0003779	actin binding	5.773×10^{-3}
4	GO:MF	GO:0015220	choline transmembrane transporter activity	2.909×10^{-2}
5	GO:BP	GO:0031589	cell-substrate adhesion	2.270×10^{-4}
6	GO:BP	GO:0030198	extracellular matrix organization	2.156×10^{-3}
7	GO:BP	GO:0007229	integrin-mediated signaling pathway	2.982×10^{-3}
8	GO:CC	GO:0008305	integrin complex	5.016×10^{-7}
9	GO:CC	GO:0005604	basement membrane	1.137×10^{-4}
10	GO:CC	GO:0030054	cell junction	3.993×10^{-4}
11	GO:CC	GO:0016020	membrane	3.713×10^{-3}
12	GO:CC	GO:0031982	vesicle	3.132×10^{-2}
13	GO:CC	GO:0030426	growth cone	4.481×10^{-2}
14	GO:CC	GO:0014069	postsynaptic density	4.580×10^{-2}

Cluster 20

**version**	e111_eg58_p18_f463989d
**date**	8/26/2024, 6:37:35 PM
**organism**	hsapiens

g:Profiler

Figure S9q

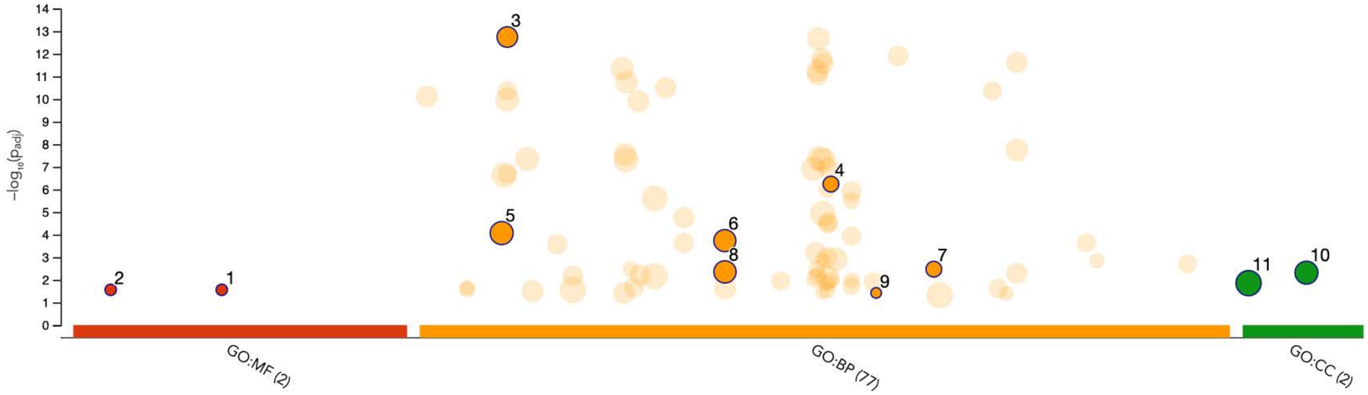

ID	Source	Term ID	Term Name	p_{adj} (query_1)
1	GO:MF	GO:0034481	chondroitin sulfotransferase activity	2.748×10^{-2}
2	GO:MF	GO:0005042	netrin receptor activity	2.748×10^{-2}
3	GO:BP	GO:0007409	axonogenesis	1.802×10^{-13}
4	GO:BP	GO:0050919	negative chemotaxis	5.790×10^{-7}
5	GO:BP	GO:0007155	cell adhesion	8.523×10^{-5}
6	GO:BP	GO:0040007	growth	1.834×10^{-4}
7	GO:BP	GO:0071526	semaphorin-plexin signaling pathway	3.368×10^{-3}
8	GO:BP	GO:0040012	regulation of locomotion	4.425×10^{-3}
9	GO:BP	GO:0060666	dichotomous subdivision of terminal units involved in salivary gland branching	3.770×10^{-2}
10	GO:CC	GO:0045202	synapse	4.807×10^{-3}
11	GO:CC	GO:0005576	extracellular region	1.394×10^{-2}

Cluster 21

**version**	e111_eg58_p18_f463989d	g:Profiler
**date**	8/26/2024, 6:38:02 PM	
**organism**	hsapiens	

Figure S9r

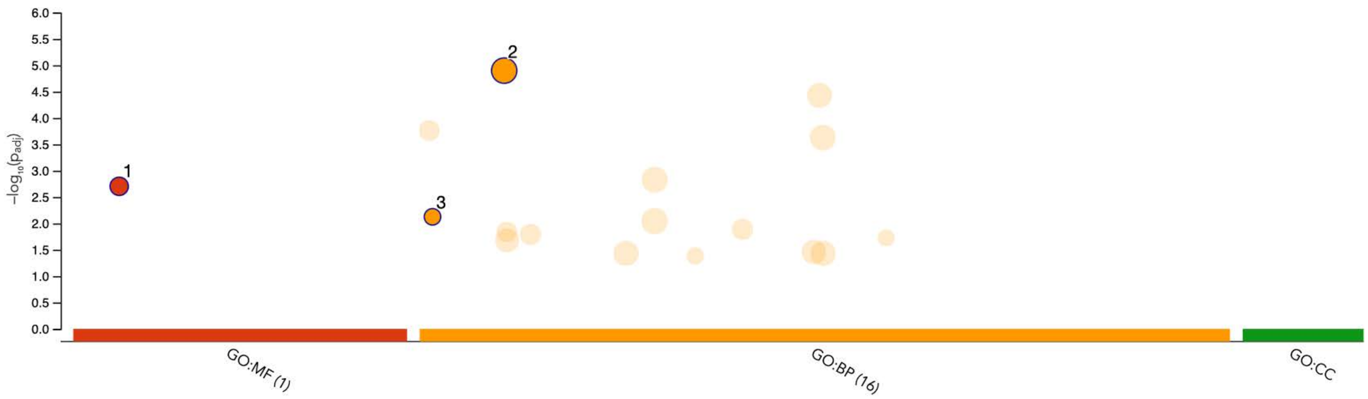

ID	Source	Term ID	Term Name	p_{adj} (query_1)
1	GO:MF	GO:0008083	growth factor activity	1.976×10^{-3}
2	GO:BP	GO:0007275	multicellular organism development	1.261×10^{-5}
3	GO:BP	GO:0001756	somitogenesis	7.482×10^{-3}

Cluster 22

**version**	e111_eg58_p18_f463989d	g:Profiler
**date**	8/26/2024, 6:38:35 PM	
**organism**	hsapiens	

Figure S9s

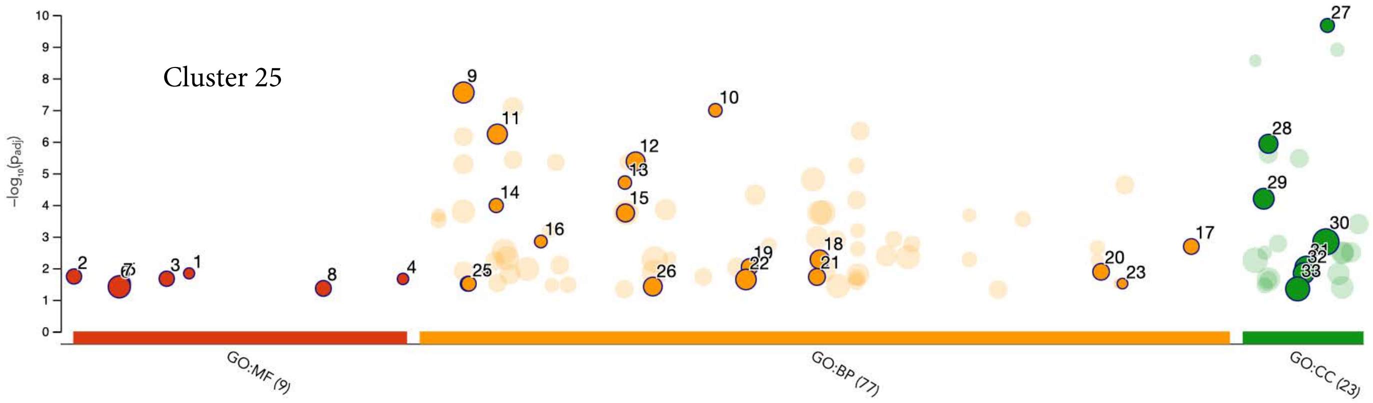

ID	Source	Term ID	Term Name	p_{adj} (query_1)
1	GO:MF	GO:0030298	receptor signaling protein tyrosine kinase activator activity	1.457×10^{-2}
2	GO:MF	GO:0000146	microfilament motor activity	1.830×10^{-2}
3	GO:MF	GO:0017080	sodium channel regulator activity	2.156×10^{-2}
4	GO:MF	GO:1990239	steroid hormone binding	2.184×10^{-2}
5	GO:MF	GO:0008307	structural constituent of muscle	2.918×10^{-2}
6	GO:MF	GO:0005391	P-type sodium:potassium-exchanging transporter activity	3.053×10^{-2}
7	GO:MF	GO:0008092	cytoskeletal protein binding	3.878×10^{-2}
8	GO:MF	GO:0060590	ATPase regulator activity	4.362×10^{-2}
9	GO:BP	GO:0003013	circulatory system process	2.800×10^{-8}
10	GO:BP	GO:0036376	sodium ion export across plasma membrane	1.017×10^{-7}
11	GO:BP	GO:0006936	muscle contraction	5.774×10^{-7}
12	GO:BP	GO:0031032	actomyosin structure organization	4.198×10^{-6}
13	GO:BP	GO:0030007	intracellular potassium ion homeostasis	1.966×10^{-5}
14	GO:BP	GO:0006883	intracellular sodium ion homeostasis	1.044×10^{-4}
15	GO:BP	GO:0030048	actin filament-based movement	1.796×10^{-4}
16	GO:BP	GO:0010248	establishment or maintenance of transmembrane electrochemical gradient	1.434×10^{-3}
17	GO:BP	GO:1990573	potassium ion import across plasma membrane	2.064×10^{-3}
18	GO:BP	GO:0048738	cardiac muscle tissue development	5.326×10^{-3}
19	GO:BP	GO:0043462	regulation of ATP-dependent activity	9.141×10^{-3}
20	GO:BP	GO:1902475	L-alpha-amino acid transmembrane transport	1.302×10^{-2}
21	GO:BP	GO:0048644	muscle organ morphogenesis	1.894×10^{-2}
22	GO:BP	GO:0043269	regulation of monoatomic ion transport	2.280×10^{-2}
23	GO:BP	GO:1903416	response to glycoside	3.069×10^{-2}
24	GO:BP	GO:0003171	atrioventricular valve development	3.105×10^{-2}
25	GO:BP	GO:0003209	cardiac atrium morphogenesis	3.105×10^{-2}
26	GO:BP	GO:0032412	regulation of monoatomic ion transmembrane transporter activity	3.816×10^{-2}
27	GO:CC	GO:0090533	cation-transporting ATPase complex	2.122×10^{-10}
28	GO:CC	GO:0030017	sarcomere	1.167×10^{-6}
29	GO:CC	GO:0015629	actin cytoskeleton	6.385×10^{-5}
30	GO:CC	GO:0071944	cell periphery	1.472×10^{-3}
31	GO:CC	GO:0045202	synapse	9.662×10^{-3}
32	GO:CC	GO:0044297	cell body	1.448×10^{-2}
33	GO:CC	GO:0042995	cell projection	4.515×10^{-2}

Custer 24

version e111_eg58_p18_f463989d

date 8/26/2024, 6:39:30 PM

organism hsapiens

g:Profiler

Figure S9t

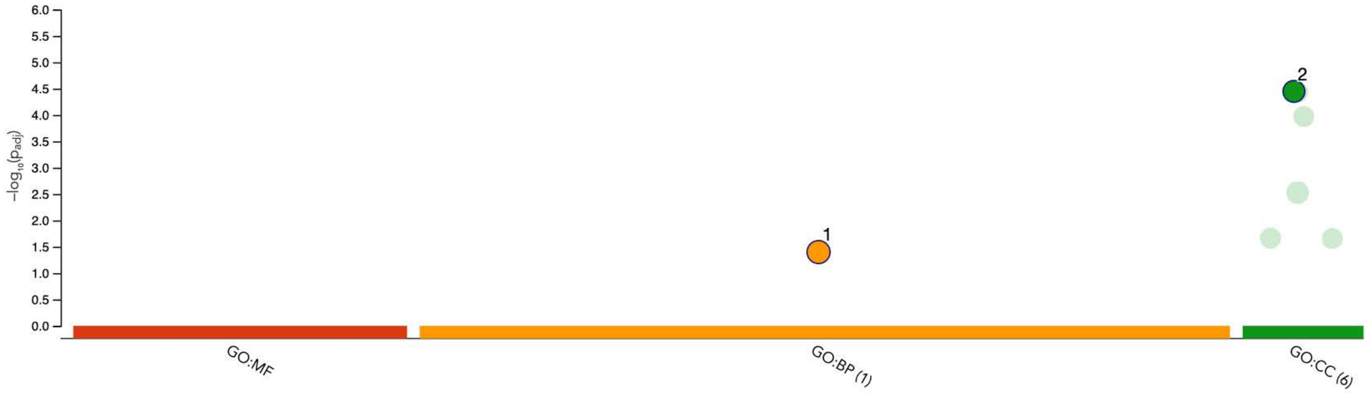

ID	Source	Term ID	Term Name	p_{adj} (query_1)
1	GO:BP	GO:0048699	generation of neurons	3.987×10^{-2}
2	GO:CC	GO:0036477	somatodendritic compartment	3.562×10^{-5}

Cluster 25

**version**	e111_eg58_p18_f463989d	g:Profiler
**date**	8/26/2024, 6:39:55 PM	
**organism**	hsapiens	

Figure S9t

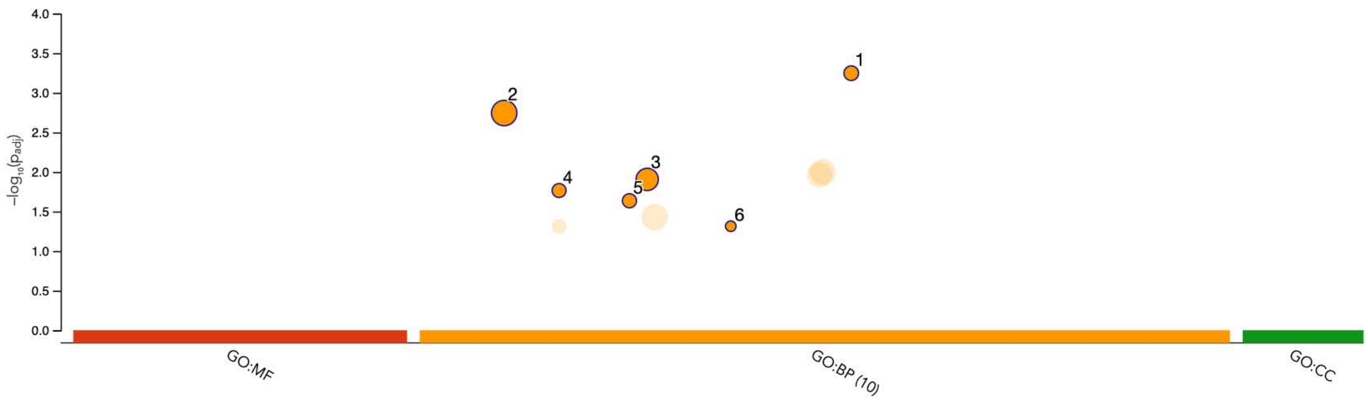

ID	Source	Term ID	Term Name	p_{adj} (query_1)
1	GO:BP	GO:0051953	negative regulation of amine transport	5.659×10^{-4}
2	GO:BP	GO:0007275	multicellular organism development	1.803×10^{-3}
3	GO:BP	GO:0032101	regulation of response to external stimulus	1.243×10^{-2}
4	GO:BP	GO:0014048	regulation of glutamate secretion	1.718×10^{-2}
5	GO:BP	GO:0030431	sleep	2.314×10^{-2}
6	GO:BP	GO:0042321	negative regulation of circadian sleep/wake cycle, sleep	4.854×10^{-2}

Cluster 1

**version**	e111_eg58_p18_f463989d	g:Profiler
**date**	8/26/2024, 6:40:31 PM	
**organism**	hsapiens	

Figure S10a

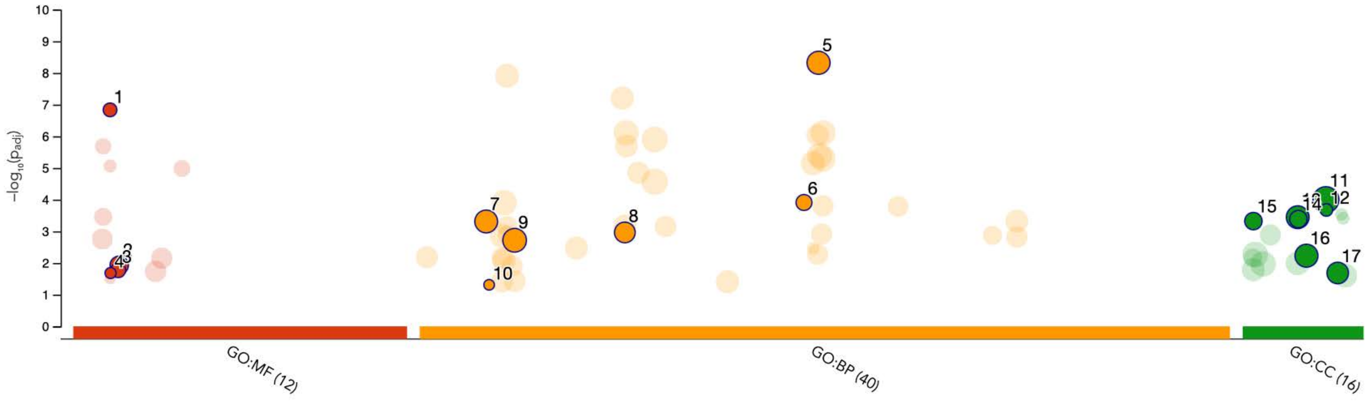

ID	Source	Term ID	Term Name	p_{adj} (query_1)
1	GO:MF	GO:0005003	ephrin receptor activity	1.461×10^{-7}
2	GO:MF	GO:0008083	growth factor activity	1.182×10^{-2}
3	GO:MF	GO:0005540	hyaluronic acid binding	1.689×10^{-2}
4	GO:MF	GO:0005030	neurotrophin receptor activity	2.078×10^{-2}
5	GO:BP	GO:0048699	generation of neurons	4.771×10^{-9}
6	GO:BP	GO:0048013	ephrin receptor signaling pathway	1.231×10^{-4}
7	GO:BP	GO:0006468	protein phosphorylation	4.888×10^{-4}
8	GO:BP	GO:0023061	signal release	1.085×10^{-3}
9	GO:BP	GO:0008283	cell population proliferation	1.918×10^{-3}
10	GO:BP	GO:0006583	melanin biosynthetic process from tyrosine	4.887×10^{-2}
11	GO:CC	GO:0071944	cell periphery	1.004×10^{-4}
12	GO:CC	GO:0072534	perineuronal net	2.121×10^{-4}
13	GO:CC	GO:0043005	neuron projection	3.582×10^{-4}
14	GO:CC	GO:0043202	lysosomal lumen	4.109×10^{-4}
15	GO:CC	GO:0005796	Golgi lumen	4.756×10^{-4}
16	GO:CC	GO:0045202	synapse	5.883×10^{-3}
17	GO:CC	GO:0098552	side of membrane	2.069×10^{-2}

Cluster 2

version e111_eg58_p18_f463989d
date 8/26/2024, 6:40:59 PM
organism hsapiens

g:Profiler

Figure S10b

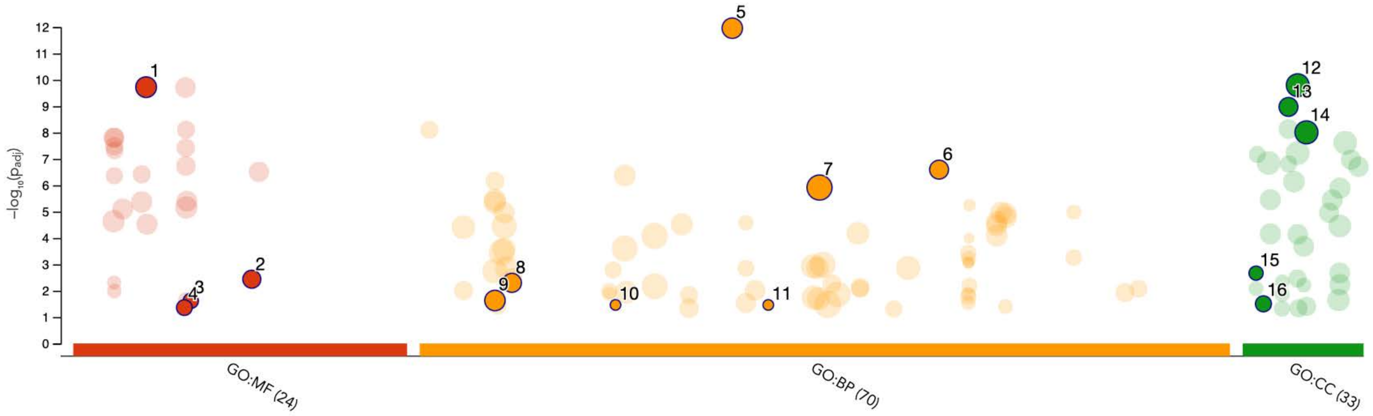

ID	Source	Term ID	Term Name	p_{adj} (query_1)
1	GO:MF	GO:0015267	channel activity	1.897×10^{-10}
2	GO:MF	GO:0044325	transmembrane transporter binding	3.652×10^{-3}
3	GO:MF	GO:0030551	cyclic nucleotide binding	2.420×10^{-2}
4	GO:MF	GO:0019894	kinesin binding	4.372×10^{-2}
5	GO:BP	GO:0042391	regulation of membrane potential	1.099×10^{-12}
6	GO:BP	GO:0071805	potassium ion transmembrane transport	2.549×10^{-7}
7	GO:BP	GO:0048731	system development	1.218×10^{-6}
8	GO:BP	GO:0007601	visual perception	5.026×10^{-3}
9	GO:BP	GO:0006816	calcium ion transport	2.344×10^{-2}
10	GO:BP	GO:0021759	globus pallidus development	3.458×10^{-2}
11	GO:BP	GO:0045163	clustering of voltage-gated potassium channels	3.458×10^{-2}
12	GO:CC	GO:0043005	neuron projection	1.595×10^{-10}
13	GO:CC	GO:0034703	cation channel complex	1.063×10^{-9}
14	GO:CC	GO:0045202	synapse	9.808×10^{-9}
15	GO:CC	GO:0005922	connexin complex	2.150×10^{-3}
16	GO:CC	GO:0014704	intercalated disc	3.139×10^{-2}

Cluster 3

**version**	e111_eg58_p18_f463989d
**date**	8/26/2024, 6:41:29 PM
**organism**	hsapiens

g:Profiler

Figure S10c

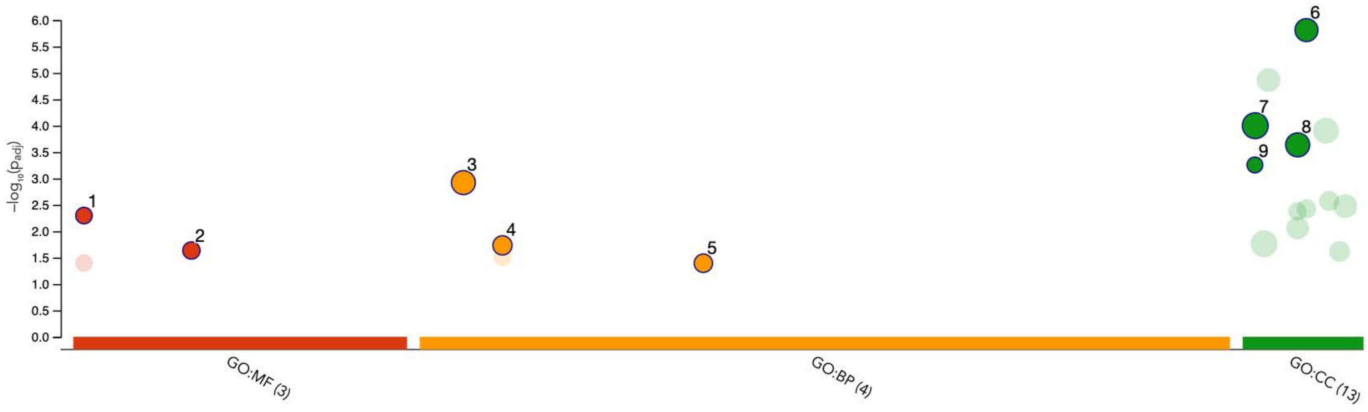

ID	Source	Term ID	Term Name	p_{adj} (query_1)
1	GO:MF	GO:0003777	microtubule motor activity	5.046×10^{-3}
2	GO:MF	GO:0030594	neurotransmitter receptor activity	2.316×10^{-2}
3	GO:BP	GO:0003008	system process	1.197×10^{-3}
4	GO:BP	GO:0007188	adenylate cyclase-modulating G protein-coupled receptor signaling pathway	1.851×10^{-2}
5	GO:BP	GO:0035725	sodium ion transmembrane transport	4.036×10^{-2}
6	GO:CC	GO:0045202	synapse	1.544×10^{-6}
7	GO:CC	GO:0005886	plasma membrane	9.999×10^{-5}
8	GO:CC	GO:0042995	cell projection	2.307×10^{-4}
9	GO:CC	GO:0005871	kinesin complex	5.542×10^{-4}

Cluster 4

**version**	e111_eg58_p18_f463989d
**date**	8/26/2024, 6:42:02 PM
**organism**	hsapiens

g:Profiler

Figure S10d

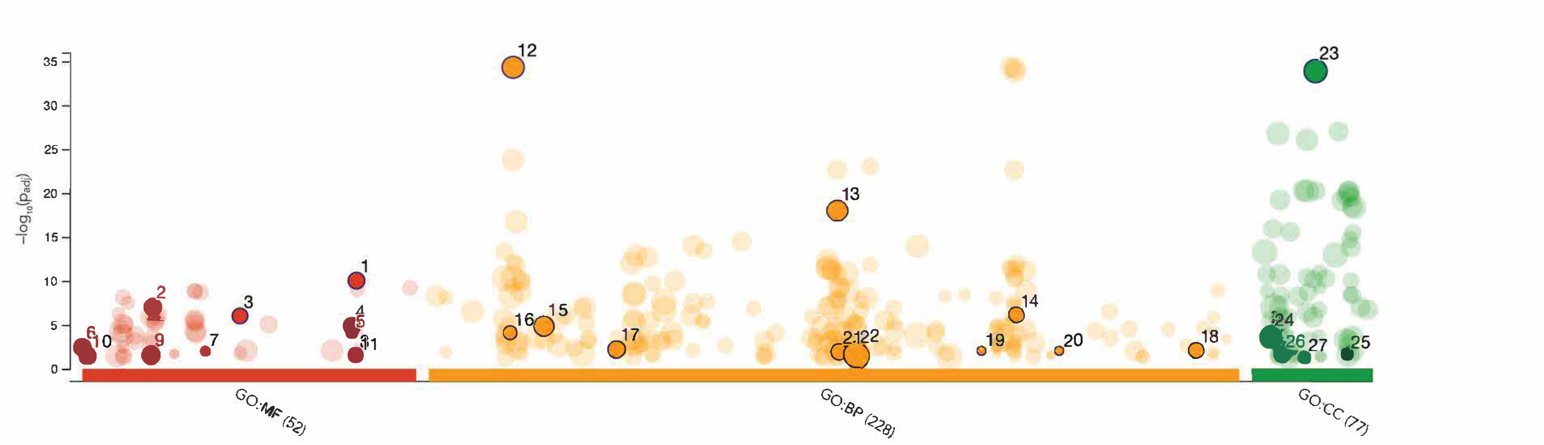

ID	Source	Term ID	Term Name	p_{adj} (query_1)
1	GO:MF	GO:0098960	postsynaptic neurotransmitter receptor activity	9.236×10^{-11}
2	GO:MF	GO:0015171	amino acid transmembrane transporter activity	9.488×10^{-8}
3	GO:MF	GO:0035254	glutamate receptor binding	9.607×10^{-7}
4	GO:MF	GO:0097110	scaffold protein binding	1.283×10^{-5}
5	GO:MF	GO:0097109	neuroligin family protein binding	8.249×10^{-5}
6	GO:MF	GO:0000149	SNARE binding	3.793×10^{-3}
7	GO:MF	GO:0031687	A2A adenosine receptor binding	1.002×10^{-2}
8	GO:MF	GO:0098918	structural constituent of synapse	2.592×10^{-2}
9	GO:MF	GO:0015079	potassium ion transmembrane transporter activity	2.984×10^{-2}
10	GO:MF	GO:0001540	amyloid-beta binding	3.327×10^{-2}
11	GO:MF	GO:0098919	structural constituent of postsynaptic density	3.330×10^{-2}
12	GO:BP	GO:0007268	chemical synaptic transmission	4.534×10^{-35}
13	GO:BP	GO:0050808	synapse organization	9.738×10^{-19}
14	GO:BP	GO:0099601	regulation of neurotransmitter receptor activity	7.018×10^{-7}
15	GO:BP	GO:0010038	response to metal ion	1.443×10^{-5}
16	GO:BP	GO:0007158	neuron cell-cell adhesion	7.726×10^{-5}
17	GO:BP	GO:0021510	spinal cord development	6.213×10^{-3}
18	GO:BP	GO:1990090	cellular response to nerve growth factor stimulus	7.820×10^{-3}
19	GO:BP	GO:0090125	cell-cell adhesion involved in synapse maturation	8.863×10^{-3}
20	GO:BP	GO:1900451	positive regulation of glutamate receptor signaling pathway	8.863×10^{-3}
21	GO:BP	GO:0050885	neuromuscular process controlling balance	1.354×10^{-2}
22	GO:BP	GO:0051716	cellular response to stimulus	2.732×10^{-2}
23	GO:CC	GO:0045202	synapse	1.159×10^{-34}
24	GO:CC	GO:0009986	cell surface	2.371×10^{-4}
25	GO:CC	GO:0098635	protein complex involved in cell-cell adhesion	2.087×10^{-2}
26	GO:CC	GO:0031045	dense core granule	2.521×10^{-2}
27	GO:CC	GO:0042584	chromaffin granule membrane	4.987×10^{-2}

Cluster 5

**version**	e111_eg58_p18_f463989d
**date**	8/26/2024, 6:42:28 PM
**organism**	hsapiens

g:Profiler

Figure S10e

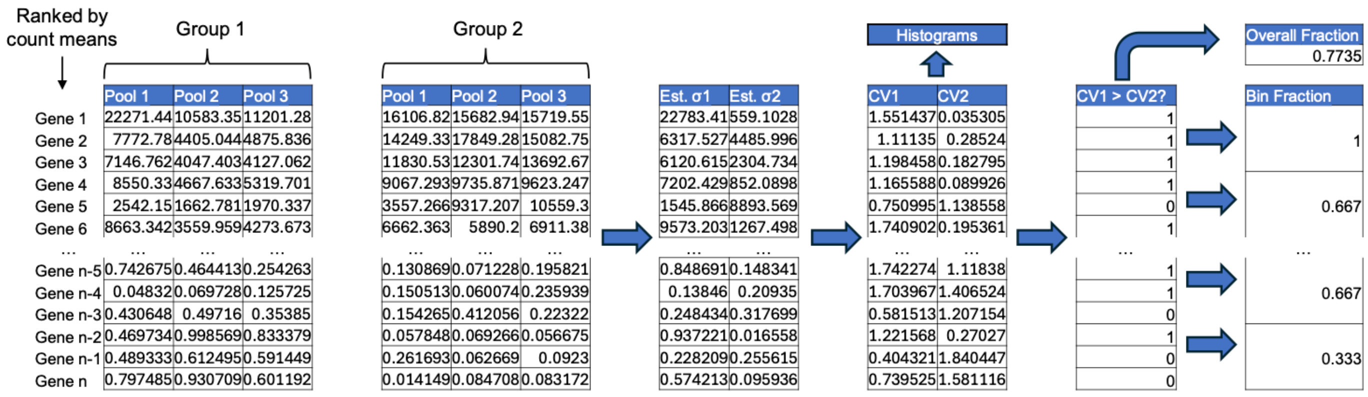

Ranked by count means
Group 1
Pool 1 Pool 2 Pool 3
Gene 1 22271.44 10583.35 11201.28
Gene 2 7772.78 4405.044 4875.836
Gene 3 7146.762 4047.403 4127.062
Gene 4 8550.33 4667.633 5319.701
Gene 5 2542.15 1662.781 1970.337
Gene 6 8663.342 3559.959 4273.673
... ...
Gene n-5 0.742675 0.464413 0.254263
Gene n-4 0.04832 0.069728 0.125725
Gene n-3 0.430648 0.49716 0.35385
Gene n-2 0.469734 0.998569 0.833379
Gene n-1 0.489333 0.612495 0.591449
Gene n 0.797485 0.930709 0.601192
Group 2
Pool 1 Pool 2 Pool 3
16106.82 15682.94 15719.55
14249.33 17849.28 15082.75
11830.53 12301.74 13692.67
9067.293 9735.871 9623.247
3557.266 9317.207 10559.3
6662.363 5890.2 6911.38
... ...
0.130869 0.071228 0.195821
0.150513 0.060074 0.235939
0.154265 0.412056 0.22322
0.057848 0.069266 0.056675
0.261693 0.062669 0.0923
0.014149 0.084708 0.083172
Est. σ1 Est. σ2
22783.41 559.1028
6317.527 4485.996
6120.615 2304.734
7202.429 852.0898
1545.866 8893.569
9573.203 1267.498
... ...
0.848691 0.148341
0.13846 0.20935
0.248434 0.317699
0.937221 0.016558
0.228209 0.255615
0.574213 0.095936
Histograms
CV1 CV2
1.551437 0.035305
1.11135 0.28524
1.198458 0.182795
1.165588 0.089926
0.750995 1.138558
1.740902 0.195361
... ...
1.742274 1.11838
1.703967 1.406524
0.581513 1.207154
1.221568 0.27027
0.404321 1.840447
0.739525 1.581116
CV1 > CV2?
1
1
1
1
0
1
...
1
1
0
1
0
0
Overall Fraction
0.7735
Bin Fraction
1
0.667
...
0.667
0.333

Figure S11
