# Supplementary material for: Engineered Living Systems With Self‐Organizing Neural Networks: From Anatomy to Behavior and Gene Expression
Source: Adv Sci (Weinh). 2026 Feb 20;13(28):e08967. doi: 10.1002/advs.202508967 (PMC13185861; doi:10.1002/advs.202508967)
Supplement: Supplementary file 2 — Supporting File 2: advs74389‐sup‐0002‐SuppMat.docx. [file ADVS-13-e08967-s002.docx]

**Figure S1.** Comparison between physical and kinematic parameters between biobots and sham neurobots. There was no significant difference between roundness index (**a**), area (**b**), complexity index (**c**) and Minimum Speed (**d**) between biobots and sham neurobots (Kruskal- Wallis test, p=0.89, 0.943,0.11 and 0.35, respectively).

**Figure S2.** Anti-synapsin-1 antibody labeling of putative synapses in neurobots. **a.** Confocal image from the center of a neurobot stained with anti-synapsin-1 primary antibody and Alexa Fluor 488–conjugated secondary antibody, showing punctate labeling consistent with putative synapses. **b.** Equivalent optical plane from a different neurobot stained with secondary antibody only, showing no detectable signal (secondary-only control). Scale bars, 40 µm.

**Figure S3.** Examples of Z-projected confocal fluorescent images of neurobots. The first two columns show staining of acetylated alpha tubulin, which labels multiciliated cells and neurons (color code represents depth in the confocal stack). The last column shows the nuclear stain in the same neurobot shown on that row, with the same set of plane shown in the middle panel. The first column shows the Z-projection of the full stack, where as the next two columns show partial stacks to reveal the interior of the neurobot. All neurobots contain processes within the bot and those that extend towards the surface. They also contain a central region with seemingly no nuclei present, which we hypothesize might be filled with extracellular matrix materials.

**Figure S4.** Structural composition of the neurobot cavity revealed by multiphoton imaging and immunostaining. Representative image of a collapsed z-stack (30 µm total thickness) of a neurobot showing: (**a**) nuclei stained with Hoechst 33342, (**b**) second harmonic generation (SHG) signal representing extracellular matrix collagen, (**c**) multi-ciliated cells and neurons labeled with acetylated α-tubulin, and (**d**) Alexa Fluor 647–conjugated phalloidin staining of filamentous actin in all cells. (**e**) Enlarged view showing all four channels overlaid. Collagen fibers and puncta-like structures (green arrows), as well as neuronal processes labeled by phalloidin alone or by both phalloidin and acetylated α-tubulin (blue and purple arrows, respectively), are visible within the cavity; however, most of the cavity appears free of nuclei, cell bodies, fibers, and collagen. Scale bars, 100 µm.

**Figure S5.** Comparison of various kinematic parameters between biobots and neurobots. There was no significant difference in total distance traveled (**a**), average speed (**b**), average acceleration (**c**) and the percentage of the well area visited (**d**) between biobots (n=22) and neurobots (n=47). (Kruskal- Wallis test, p=0.5, 0.5,0.28 and 0.43, respectively)

**Figure S6.** Neurobots had a significantly smaller density of multiciliated cells. Kruskal- Wallis test was used to obtain the p-value (p=0.027).

**Figure S7.** Impact of treatment with zolmitriptan on neural expression patterns in neurobots. **a-c** Treatment with zolmitriptan increased number of terminals, total length of neurites and neurite density. There was no significant change in complexity index (Kruskal- Wallis test, p=0.05, 0.04,0.1 and 0.03, respectively) (**d**). **e.** Pairwise correlation between structural parameters of zolmitriptan-treated neurobots and Complexity Index, N_terminals_ = total number of endings, L_Neurite_=total length of neurites, L_Neuritenorm_=total length of neurites normalized to area, N_MCC_=total number of multiciliated cells on the top surface, N_MCCnorm_= N_MCC_ normalized to area, RI= Roundness Index, Neu/Ect= ratio of the areas of neural implant to ectoderm shell. Pearson correlation coefficients are depicted for each pair. Values in red correspond to statistically significant correlations (two-tailed Student’s t-test, P<0.05).

**Figure S8.** Enrichment analysis performed using Gene Ontology annotations **a**. sham neurobots vs biobots (4-fold upregulated pathways) and **b.** neurobots with biobots (4- fold downregulated pathways)

**Figure S9.** Enriched pathways in neurobots when compared to biobots, across clusters identified based on network analysis.

**Figure S10.** Enriched pathways in sham neurobots when compared to biobots, across clusters identified based on network analysis.

**Figure S11.** Method for quantifying variability in gene expression. For a chosen pair of groups, genes were ranked by the mean count value across all pools of both groups, and the CV of each gene’s counts across the pools of each group was calculated. The CV list was split into 100 bins (percentiles) containing equal numbers of genes, and the fraction of genes in the bin for which the CV of the first group was greater than that of the second group was found and plotted.
