# Supplementary material for: Engineered Living Systems With Self‐Organizing Neural Networks: From Anatomy to Behavior and Gene Expression
Source: Adv Sci (Weinh). 2026 Feb 20;13(28):e08967. doi: 10.1002/advs.202508967 (PMC13185861; doi:10.1002/advs.202508967)
Supplement: Supplementary file 7 — Supporting File 7: advs74389‐sup‐0007‐SuppMat.docx. [file ADVS-13-e08967-s001.docx]

Supplementary Video Legends

Supp. Vid. 1. Example of calcium activity measured in a freely moving neurobot implanted with GCaMP labeled neural precursor cells. Video is sped up 120 times.

Supp. Vid. 2. Same video as in Supp. Vid. 1, after motion correction. Video is sped up 120 times.

Supp, Vid. 3. A neurobot moving within a well of an 8-well plate. Video is sped up 10 times.

Supp. Vid. 4. Exemplar bots moving in 8 well plates. The left plate contains Biobots and the right one neurobots. Video is sped up 30 times.
